# The Role of *Eif6* in Skeletal Muscle Homeostasis Revealed by Endurance Training Co-expression Networks

**DOI:** 10.1016/j.celrep.2017.10.040

**Published:** 2017-11-07

**Authors:** Kim Clarke, Sara Ricciardi, Tim Pearson, Izwan Bharudin, Peter K. Davidsen, Michela Bonomo, Daniela Brina, Alessandra Scagliola, Deborah M. Simpson, Robert J. Beynon, Farhat Khanim, John Ankers, Mark A. Sarzynski, Sujoy Ghosh, Addolorata Pisconti, Jan Rozman, Martin Hrabe de Angelis, Chris Bunce, Claire Stewart, Stuart Egginton, Mark Caddick, Malcolm Jackson, Claude Bouchard, Stefano Biffo, Francesco Falciani

**Affiliations:** 1 Institute of Integrative Biology, University of Liverpool, Liverpool L69 7ZB, UK; 2 Molecular Histology and Cell Growth Unit, INGM - Fondazione Istituto Nazionale Genetica Molecolare, 20122 Milan, Italy; 3 Department of Medicine, University of East Anglia, Norwich Research Park, Norwich NR4 7TJ, UK; 4 School of Biosciences and Biotechnology, Universiti Kebangsaan Malaysia, 43600 Bangi, Selangor, Malaysia; 5 Centre for Proteome Research, Institute of Integrative Biology, University of Liverpool, Liverpool L69 7ZB, UK; 6 School of Biosciences, University of Birmingham, Birmingham B15 2TT, UK; 7 Pennington Biomedical Research Center, Baton Rouge, LA 70808, USA; 8 German Mouse Clinic, Institute of Experimental Genetics, Helmholtz Zentrum München, German Research Center for Environmental Health, Ingolstädter Landstr. 1, 85764 Neuherberg, Germany; 9 Sport and Exercise Sciences, Liverpool John Moores University, Liverpool L3 3AF, UK; 10 School of Biomedical Sciences, University of Leeds, Leeds LS2 9JT, UK; 11 Institute of Ageing and Chronic Disease, University of Liverpool, Liverpool L7 8TX, UK; 12 Dipartimento di Bioscienze, Università degli Studi di Milano, 20133 Milan, Italy

**Keywords:** skeletal muscle, exercise, *Eif6*, systems biology, metabolism, mitochondria, network biology

## Abstract

Regular endurance training improves muscle oxidative capacity and reduces the risk of age-related disorders. Understanding the molecular networks underlying this phenomenon is crucial. Here, by exploiting the power of computational modeling, we show that endurance training induces profound changes in gene regulatory networks linking signaling and selective control of translation to energy metabolism and tissue remodeling. We discovered that knockdown of the mTOR-independent factor *Eif6*, which we predicted to be a key regulator of this process, affects mitochondrial respiration efficiency, ROS production, and exercise performance. Our work demonstrates the validity of a data-driven approach to understanding muscle homeostasis.

## Highlights

• Endurance exercise profoundly affects the structure of gene networks
• *Eif6* is a hub in gene networks responsible for muscle metabolism and protein synthesis
• Mitochondrial metabolic capacity altered in muscle from *Eif6*^+/−^ mice
• Eif6 haploinsufficiency increased ROS generation and reduced exercise performance

## Graphical Abstract

**Figure:**
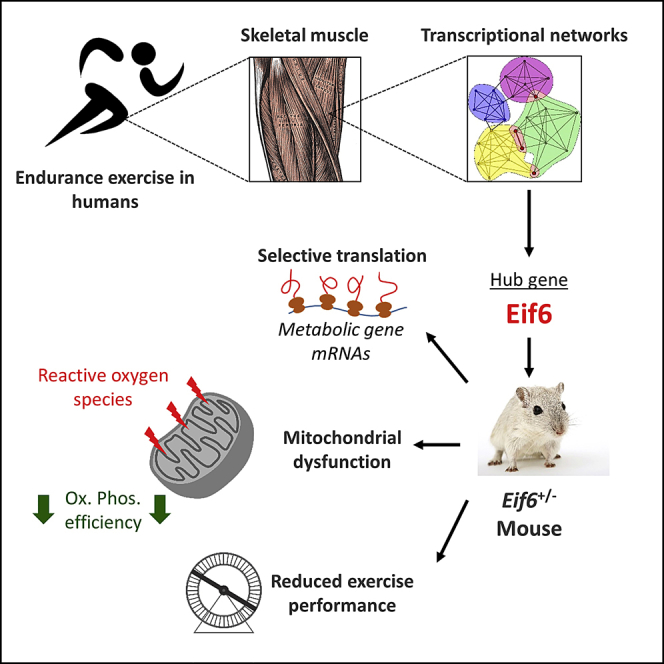

## Teaser

Clarke et al. use data-driven reverse engineering to uncover the role of *Eif6* in controlling skeletal muscle homeostasis. They achieve this by analyzing the complex network of genes that controls skeletal muscle adaptation to endurance exercise, together with *in vivo* studies of *eif6*^+/−^ mice that show decreased respiration efficiency, increased ROS production, and reduced exercise performance.

## Introduction

Physical activity or regular exercise is essential for homeostasis of the musculoskeletal system. The benefits of regular exercise are such that physically active individuals have significantly lower mortality rates, regardless of age and lifestyle (Kokkinos, 2012). Exercise is recognized as an effective strategy for the management of chronic conditions such as obesity (Blair and Brodney, 1999), type 2 diabetes (Colberg et al., 2010), and chronic obstructive pulmonary disease (COPD) (Turan et al., 2011).

In the last decade, transcriptomics studies have succeeded in revealing genome-wide transcriptional signatures linked to the endurance training response (Phillips et al., 2013, Timmons et al., 2010, Teran-Garcia et al., 2005, Schmutz et al., 2006). However, most of these studies are also limited to a description of the differentially expressed genes and the functions that they represent. Perhaps the most advanced approach in a recent study is demonstrated by Phillips et al. (2013), who use a correlation-based approach to identify genes linked to gains in muscle mass or age. By utilizing pathway databases representing knowledge-driven gene-to-gene interactions, they identify molecular networks underlying both aging and muscle hypertrophy. While their study highlighted that training induces a molecular reprogramming of the muscle, including showing that the differential expression of mTOR-related genes is linked to muscle hypertrophy, it still relies on existing knowledge for pathway identification.

We reasoned that a data-driven approach may successfully identify truly novel regulatory networks. Encouraged by previous approaches (Turan et al., 2011, Davidsen et al., 2014), we selected one of the most comprehensive endurance training studies in humans, the HERITAGE Family Study (Bouchard et al., 1995), and applied a reverse-engineering strategy to develop a genome-wide network model of muscle homeostasis.

The analysis of the model revealed that co-expression of genes found within core metabolic and translational pathways, such as oxidative phosphorylation and ribosome assembly, is a predominant feature of these networks and that the eukaryotic translation initiation factor *Eif6* (Ceci et al., 2003, Sanvito et al., 1999) is the most connected ribosome associated factor in the network. Analysis of the *Eif6*^*+/−*^ haploinsufficient mouse recapitulated the transcriptional signatures predicted by the HERITAGE study. Furthermore, an assessment of mitochondrial functionality and muscle performance in *Eif6* heterozygote mice revealed alterations in electron transport chain dynamics, superoxide generation, and reduced exercise capacity, further supporting the role of this factor in regulating muscle energy homeostasis.

## Results

### Endurance Training Affects Gene Regulatory Networks in Human Skeletal Muscle

We first identified gene networks whose structure is different between pre- and post-training skeletal muscles. We did this by applying the bioinformatics method DiffCoEx (Tesson et al., 2010) to the HERITAGE study, which includes transcriptome and physiological measurements (Table S1) for 41 human participants at baseline and after 20 weeks of supervised endurance training.

**Figure 1:**
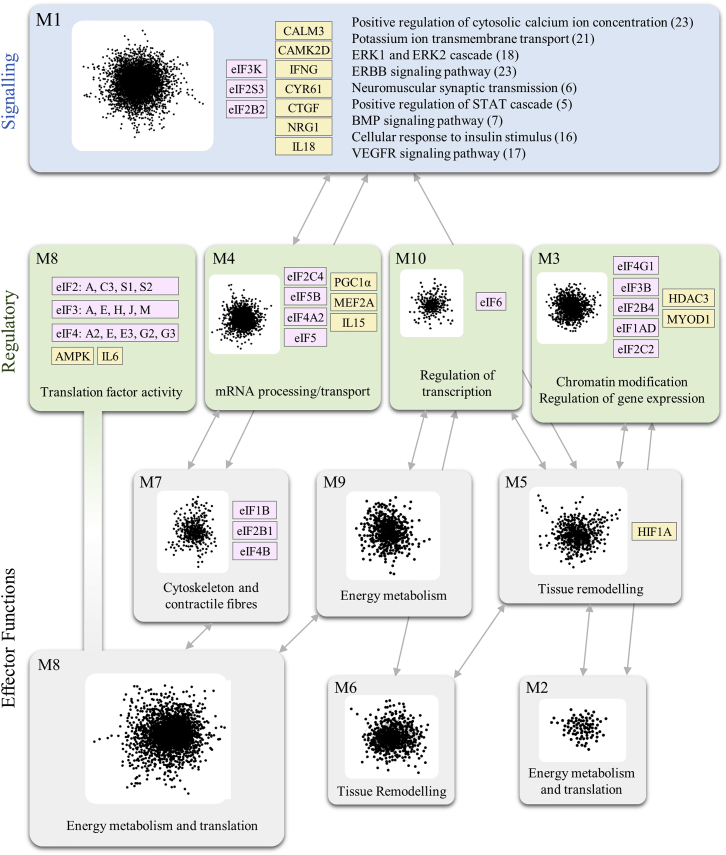
Effects of Endurance Training on Gene Co-expression in Skeletal Muscle This network represents the 10 largest gene modules identified by the gene co-expression analysis (module size is proportional to the number of genes). Genes within each module showed highly significant alteration in gene-gene pairwise correlation between trained and untrained states (FDR < 1%). Modules connected by arrows showed significant inter-module alterations in correlation between trained and untrained states (FDR < 10%). Each module has been annotated with genes known to play a key role in muscle biology (gold) and in the eukaryotic initiation factors (pink) found within that module. M1 is labeled with enriched gene ontology terms reflecting signaling pathways. Every other module has been summarized using a functional term representative of the enriched gene ontology terms.

The inferred network (Figure 1) confirmed the hypothesis that endurance training promotes a large-scale rewiring of transcriptional networks. First, we identified differential co-expression networks involving 8,893 unique genes, which the algorithm organized into 25 interconnected gene clusters (network modules) (Figure S1). Of these 8,893 genes, 981 (11%) were detected as differentially expressed in response to training, and only 3 of the 25 modules were enriched with genes responding to training (Figure S1). Network rewiring via differential co-expression therefore involves a larger set of genes and functions than identified by changes in mRNA expression levels alone. Larger modules (more than 10 genes in size) were tested for functional enrichment. Indeed, their functional profile was consistent with skeletal muscle homeostasis and included biological processes related to energy metabolism, muscle fiber contractile elements, and tissue remodeling (Figure 1).

We found that the network reflects several aspects of skeletal muscle homeostasis, including neuromuscular junction signaling, modulation of regulatory factors, and, in turn, the effector functions responsible for the endurance training response. Module 1 (M1) was enriched in cell signaling pathways including genes controlling neuromuscular synaptic transmission. M3, M4, M8, and M10 represented a set of modules characterized by regulatory factors controlling processes such as chromatin modification, transcription, mRNA processing, mRNA transport, and regulation of translation. Finally, M2 and M5–M9 represented three different types of effector functions, namely energy metabolism, tissue remodeling, and muscle fiber structural components.

The linkage between signaling and effector functions was consistent with existing literature. However, we also observed that the correlation patterns between genes in energy metabolism (oxidative phosphorylation, the tricarboxylic acid [TCA] cycle, and glycolysis) and translation were different in pre- and post-training individuals (Figure 1). Interestingly, most of the enriched translation related genes (in M8 and M2) were not ribosomal structural components. Instead, we could identify regulatory factors such as the eukaryotic initiation factors (eIFs) (14/59 translation-related genes) (Figure 1), which are known to control translation of specific proteins in the rate-limiting phase of initiation and have previously been shown to participate in signaling events such as hypoxia (*Eif2a*) (Liu et al., 2006), mTOR-dependent regulation of energy metabolism (*Eif4e*) (Morita et al., 2013), neoplastic transformation (*Eif6*) (Sanvito et al., 1999, Gandin et al., 2008), and lipid metabolism (*Eif6*) (Brina et al., 2015).

### Analysis of the Ribosome Assembly Sub-network Identifies *Eif6* as a Potential Regulator of Energy Metabolism in Skeletal Muscles

**Figure 2:**
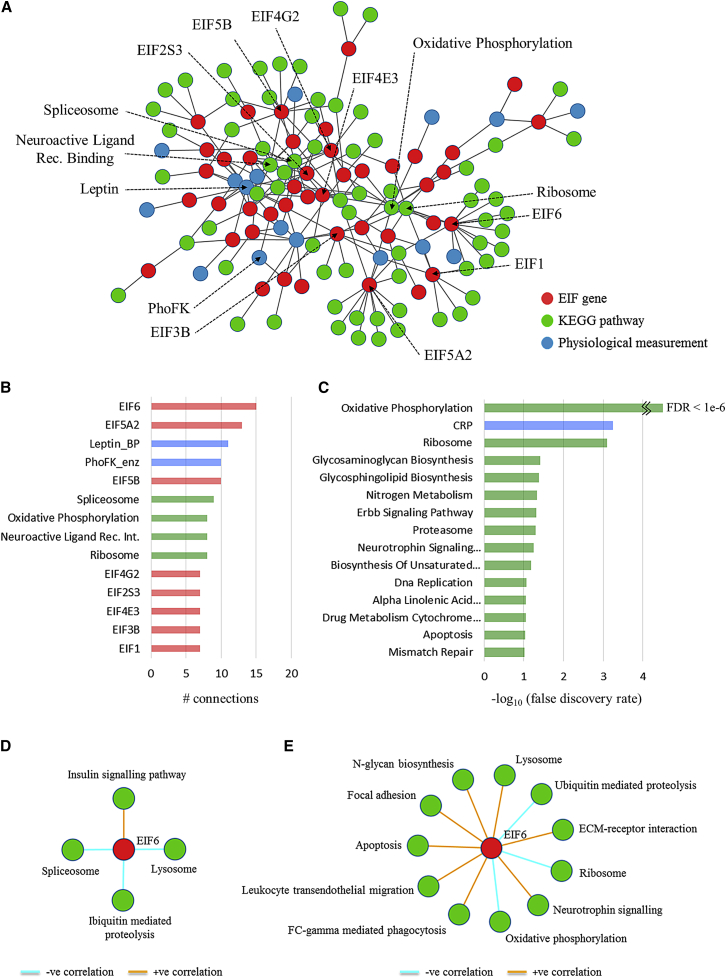
Network-Based Integration of EIF Genes, Physiological Measurements, and Pathway Activity Reveals *Eif6* as the Most Connected Factor (A) This network represents the integration of multiple analyses. Connections between eIF genes and physiological measurements represent regression analysis using baseline eIF gene expression as a predictor variable (FDR < 10%). Connections between eIF genes and KEGG pathways represent significant enrichment of genes from a KEGG pathway within genes highly differentially co-expressed with the eIF. (B) The total number of connections (degree) of the top 14 most highly connected components of the network (degree > 6). (C) Significance of enrichment of KEGG pathways and phenotypic measurements connected to *Eif6* in the network. (D and E) Network visualization of KEGG pathways enriched (FDR < 10%) within genes significantly correlated (FDR < 5%) with *Eif6* expression in untrained (n = 2,177 genes) (D) and trained (n = 2,022 genes) (E) individuals separately.

The co-localization of eIF genes and genes related to energy metabolism and other important functions involved in muscle homeostasis suggests that eIF proteins may have a broad role in the response to endurance training. To further investigate this hypothesis, we analyzed the neighborhood of eIFs in the differential co-expression network. We focused on visualizing the linkage between relevant canonical pathways (Kyoto Encyclopedia of Genes and Genomes [KEGG] pathways) and a set of 20 relevant physiological measurements (Table S1) included in the HERITAGE study (Figure 2A).

Consistent with the previous observation, we found that oxidative phosphorylation, ribosome, spliceosome, and neuroactive ligand receptor interaction were the most connected KEGG pathways among the eIF sub-network (Figure 2B). The network also represented several highly connected physiological measurements (leptin, phosphofructokinase). Interestingly, *Eif6* was the network node with the highest degree (Figure 2B), which prompted us to explore its neighborhood in detail (Figure 2C). We found that its most significant links were with genes involved in oxidative phosphorylation (false discovery rate [FDR] < 10^−6^ for pathway enrichment), followed by an index of systemic inflammation (C-reactive protein, FDR < 0.001) and, as expected, the ribosome KEGG pathway (FDR < 0.001).

Inspection of separate baseline and post-training correlation networks built around *Eif6* revealed an increased connectivity (degree of 4 increased to degree of 11) between *Eif6* and KEGG pathways following endurance training (Figures 2D and 2E).

### The Haploinsufficient *Eif6*^*+/−*^ Mouse Mimics the Predicted *Eif6* Transcriptional Signature

The correlation analysis described above predicts that changes in the activity of *Eif6* may be causally linked to changes in the expression of genes involved in several important pathways, among which oxidative phosphorylation was the strongest candidate (Figure 2C).

We then decided to test this hypothesis by assessing whether modulation of *Eif6* in skeletal muscle of mice would result in the same *Eif6* transcriptional signature that we predicted from the HERITAGE dataset. While human *vastus lateralis* muscle is considered a mixed fiber type (Staron et al., 2000), it is also highly variable (Simoneau and Bouchard, 1989), and different fiber types induce different transcriptional programs (Wang et al., 2004). To account for this and to assess the effect of *Eif6* modulation in conditions of different metabolic pathway activity we analyzed three different skeletal muscles (*soleus*, *gastrocnemius*, and *tibialis anterior*) in wild-type (WT) and mutant *Eif6*^*+/−*^ mice. These represent a preference for slow-twitch oxidative, mixed fiber type and fast-twitch, glycolytic fibers, respectively. The *Eif6*^*+/−*^ mouse model shows a 50% reduction in eIF6 expression (Gandin et al., 2008), but this does not affect basal translational rates and does not induce any changes in fiber-type composition (Figure S2A; Table S2) or in the number of mitochondria (measured as mtDNA-to-nuclear DNA [nDNA] ratio) (Figure S2B). Therefore, these animals are a good model for studying the specific role of *Eif6* in skeletal muscles.

**Figure 3:**
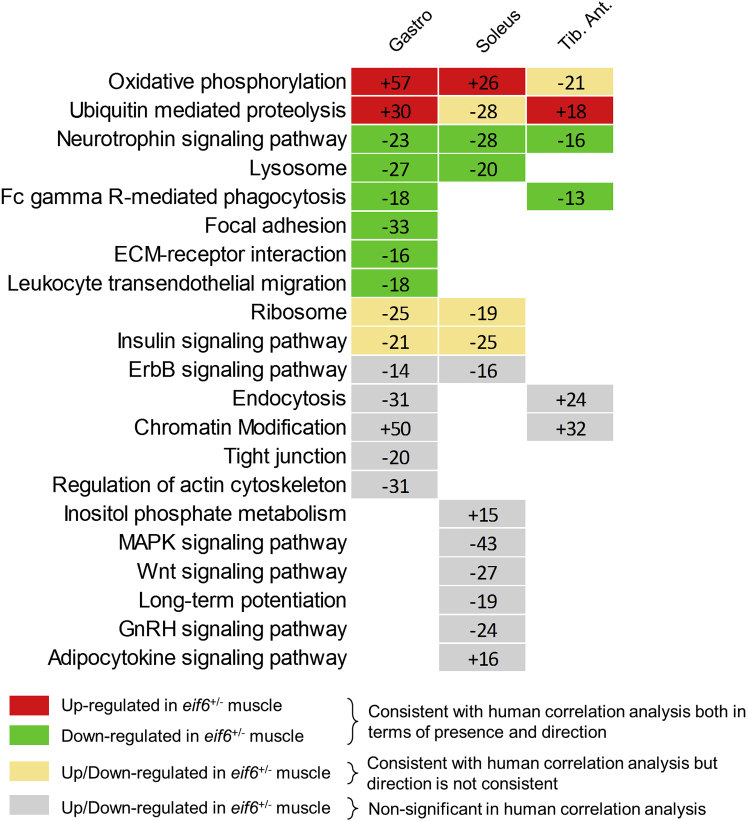
*Eif6* Haploinsufficiency in Mouse Skeletal Muscle Recapitulates the Predictions from the Human Model The figure shows the KEGG pathways enriched in genes up- or downregulated in the three *Eif6*^*+/−*^ skeletal muscle types compared to WT of the same muscle type (FDR < 10%). The number and the direction of change (positive, upregulated; negative, downregulated) of the enriched genes are indicated. Pathways colored red and green represent those matching the human *Eif6* correlation analysis (shown in Figure 3D and 3E) of consistency between direction of correlation and change in expression. Pathways highlighted in yellow represent those terms matching the human correlation analysis but without consistency between direction of correlation and change in expression.

Indeed, we discovered that the transcriptional states of WT and *Eif6*^*+/−*^ mice were different (genes differentially expressed in the *gastrocnemius*: 2,651 upregulated, 829 downregulated; soleus: 2,482 upregulated, 1,846 downregulated; *tibialis anterior* (TA): 1,361 upregulated, 958 downregulated; FDR < 10%) and that the profile of the *gastrocnemius* muscle mirrored quite accurately the predicted *Eif6* signature both in functional profile and in direction of change. More precisely, we found 20 KEGG pathways significantly enriched within the differentially expressed genes from at least 1 muscle type (Figure 3). *Gastrocnemius* muscle had the largest overlap between pathways identified in both mouse and human analysis, with 8/14 (57%) KEGG pathways altered in a direction that was consistent with the correlation analysis using the human data (Figure 3). For these reasons, we decided to focus on *gastrocnemius* muscle in further analysis. Genes both differentially expressed in the *Eif6*^*+/−*^ *gastrocnemius* muscle and correlated to the expression of *Eif6* in the HERITAGE dataset were enriched with genes related to energy metabolism, including oxidative phosphorylation complexes (e.g., *Atp5l*, *Ndufs4*), mitochondrial function (e.g., *Sod2*, *Gfm2*), and glucose metabolism (e.g., *Pdhb*) (Figure 3). Enriched functions unrelated to energy metabolism included extracellular matrix (ECM)-receptor interaction (e.g., *Thbs3*, *Tnxb*), focal adhesion (e.g., *Itgb1*), ubiquitin-mediated proteolysis (e.g., *Stub1*, *Tceb2*), ribosome, ErbB signaling (e.g., *Akt2*, *Mapk3*), and regulation of actin cytoskeleton (e.g., *Fgfr1*, *Wasf2*) (Figure 3). Interestingly, a group of genes related to chromatin modification were enriched within genes regulated by *Eif6*, particularly histone deacetylases (e.g., *Sirt6*, *Hdac6*) (Table S3), suggesting activity at both the transcriptional and the post-transcriptional levels.

These results further support the hypothesis that *Eif6* is an important regulator of the transcriptional response to endurance exercise and identify a set of core functions whose transcription is affected by *Eif6* haploinsufficiency.

### *Eif6* Inactivation Induces Complex Alterations in the Mitochondria Proteome

**Figure 4:**
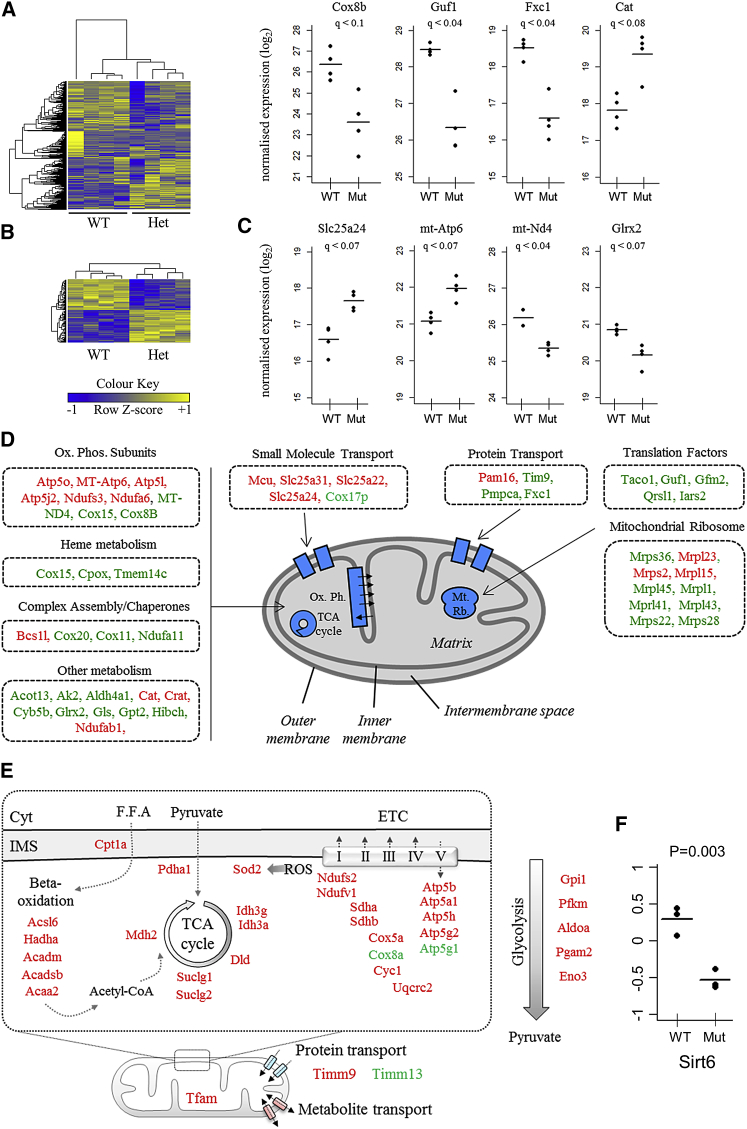
Functional Genomics Profiling of *Eif6*^*+/−*^ Muscle Reveals Substantial Mitochondrial Reprogramming (A and B) Representation of the proteomics data using a heatmap. Row data are standardized for visualization purposes. Shown are all proteins for which a measurement was obtained for all 8 samples (918 proteins) (A). Shown are the differentially expressed proteins with a FDR < 10% (120 proteins) (B). (C) Vertical dot plots representing a selection of the differentially expressed mitochondrial factors revealed by the proteomics analysis (FDR < 10%). (D) Diagrammatic representation of proteomics data showing differentially expressed mitochondrial proteins (green, downregulated; red, upregulated; FDR < 10%). (E) Diagrammatic representation of polysomal mRNA analysis of *Eif6*^*+/−*^ and WT skeletal muscle showing altered mRNA loading of mitochondria-related genes; green, downregulated; red, upregulated; absolute log_2_-fold change > 2, p < 0.05. (F) Ratio of polysomal versus total *Sirt6* mRNA in WT and *Eif6*^*+/−*^ muscle, p = 0.003.

To assess the functional consequences of *Eif6* haploinsufficiency, we used nanoflow liquid chromatography coupled to tandem mass spectrometry to characterize the mitochondrial proteome from *gastrocnemius* muscle of WT and *Eif6*^*+/−*^ mice (Figure 4A). This revealed 120 differentially expressed proteins (< 10% FDR) (Figure 4B; Table S7) including factors involved in oxidative phosphorylation and in diverse mitochondrial processes (Figures 4C and 4D). Components of the oxidative phosphorylation pathway, mitochondrial ribosome, and mitochondrial inner membrane (such as calcium channels) were both up- and downregulated (Figure 4D). Notable upregulated proteins included small-molecule transporters such as the calcium uniporter MCU, the phosphate carrier SCMC1, the cytoprotective enzyme catalase (CAT), and the translocation factors PAM16 and PAM9. Conversely, downregulated proteins included translocation factors TIM9, TIMM10B, and mitochondrial-processing peptidase subunit alpha; mitochondrial translation factors TACO1, GUF1, RRF2mt, and Glu-AdT subunit A; the oxidoreductase GLRX2; and mitochondrial protein complex assembly chaperones COX20, COX11, and NDUFA11.

In summary, we show that *Eif6* knockdown results in profound changes in mitochondrial protein composition, likely affecting the control of energy metabolism *in vivo*.

### *Eif6* Regulates the Translation of a Specific Set of Transcripts Encoding for Energy Metabolism-Related Proteins

Having observed changes at both mRNA and protein levels and given the known role of eIF6 in translation initiation, we tested whether *Eif6* haploinsufficiency leads to preferential translation of a subset of mRNAs in mouse *gastrocnemius* muscle using polysomal microarray analysis (Figures 4E and S3). We found that 346 unique mRNAs were significantly enriched in ribosomes from *Eif6*^*+/−*^ or WT mice (p *<* 0.05 and >2-fold) (Table S8). These were dominated by genes related to metabolism and mitochondrial function such as oxidative phosphorylation, acetyl-coenzyme A (CoA) metabolism, TCA cycle, glycolysis, fatty acid metabolism, and pyruvate metabolism (Figure 4E). Further analysis revealed that a key epigenetic regulatory factor linked to metabolic homeostasis, *Sirt6* (Zhong et al., 2010), that was also correlated to and regulated by *Eif6* in human and mouse muscle, respectively, was differentially translated (Figure 4F).

Highly translated mRNAs may be among the most abundant proteins in the cell. Consequently, we may expect that, despite the influence of mitochondrial protein transport and degradation, differences in the mitochondrial proteome may at least partially match the results of the polysome profiling analysis. We therefore tested whether differentially expressed proteins in the mitochondria were also differentially translated. We discovered that 41 proteins (∼30% of the total number of differentially expressed proteins in the mitochondria) were enriched in the polysomal fraction of highly translated mRNAs (Table S4). This percentage is significantly higher than expected by random chance (FDR < 10^−3^).

These results reveal that alterations in abundance of transcripts observed in *Eif6*^*+/−*^ muscle are accompanied by complex changes at the mRNA loading and protein level and suggest that these could be accompanied by changes in epigenetic status.

### Acetylation Status of Metabolic Enzymes Is Altered in *Eif6*^*+/−*^ Muscle

Several genes involved in the control of protein acetylation were differentially regulated at the mRNA level (including *Sirt1*, *Sirt6*, *Hdac1*, *Hdac6*, *Hdac7*, and *Hdac9*) and differentially translated (including *Sirt4*, *Sirt6*) (Table S3). Since changes in protein acetylation is a well-known mechanism to regulate enzymatic activity (Zhao et al., 2010), it is possible that changes in protein acetylation may be one of the mechanisms by which *Eif6* controls muscle physiology. Because of the diversity of genes involved in the control of protein acetylation, we couldn’t make any specific hypothesis on the proteins that may be affected or the direction of change.

**Figure 5:**
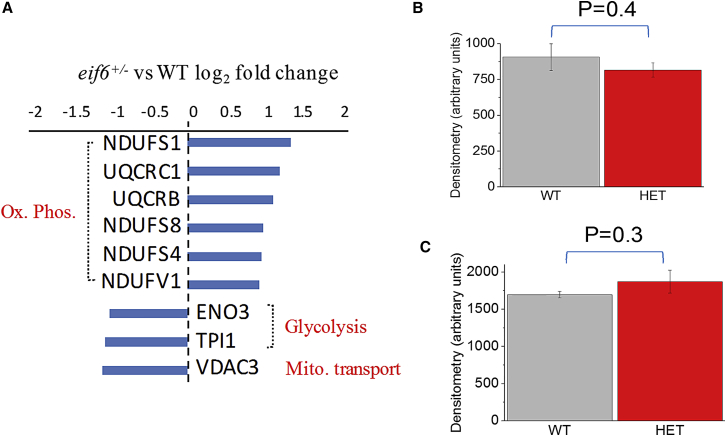
*Eif6* Haploinsufficiency Induces Alterations in Protein Acetylation (A) Differential protein acetylation in whole-skeletal-muscle lysates determined by MS (p < 0.05, absolute log_2_-fold change > 1). (B and C) Protein expression of NDUFS4 (B) and ENO3 (C) determined by western blotting in wild-type (WT) and *Eif6*^*+/−*^ (HET) skeletal muscle. Data are represented as mean ± SEM.

We therefore tested this hypothesis by using an open-ended approach based on mass spectrometry of muscle tissue protein extracts immunoprecipitated using an anti-lysine antibody. The mass spectrometry analysis on whole-*gastrocnemius*-muscle lysates revealed 19 differentially acetylated proteins (p < 0.05) (Table S5) including a coordinate hyper-acetylation of oxidative phosphorylation components, including four complex I and two complex III subunits, and reduced acetylation of two subunits of glycolytic enzymes ENO3 and TPI1 and the mitochondrial metabolite transporter VDAC3 (Figure 5A). Since acetylation can change activity without changing protein levels, we performed a western blot analysis of two of the differentially acetylated proteins (ENO3 and NDUSF4) and showed that no changes in protein levels were detected (Figures 5B, 5C, and S4).

### Skeletal Muscle of *Eif6*^*+/−*^ Mice Is Less Efficient in Utilizing Oxygen for Respiration and Develops High Levels of Reactive Oxygen Species

**Figure 6:**
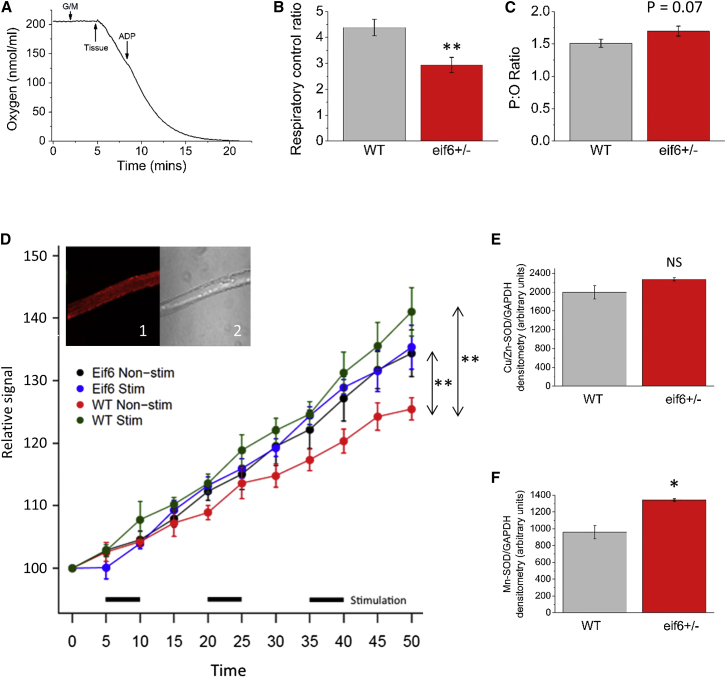
Respiration and ROS Generation in *Eif6* Heterozygote Muscle (A) Typical oxygen utilization dynamics of muscle tissue before and after addition of ADP to the medium. (B) Respiratory control ratio (ratio of respiration state 3 to state 4) is significantly lower in *Eif6*^*+/−*^ (n = 8) muscle compared with WT muscle (n = 8). (C) P:O ratio is not significantly altered in *Eif6* heterozygous muscle. (D) Mitosox (405-nm) signal (superoxide abundance) over time in response to electrically stimulated contraction in WT and *Eif6*^*+/−*^ FDB muscle fibers. Black bars indicate periods of stimulation. Repeated-measures p value: WT versus WT stimulated, p = 0.0016, *Eif6*^*+/−*^ versus *Eif6*^*+/−*^ stimulated, p = 0.38, WT versus *Eif6*^*+/−*^ non-stimulated, p < 0.01. (E and F) SOD1 (E) and SOD2 (F) protein expression measured by western blot in *Eif6*^*+/−*^ muscle. Data are represented as mean ± SEM. ^∗^p < 0.05, ^∗∗^p < 0.01.

Our functional genomics analyses suggested a significant alteration in mitochondrial energy metabolism. The nature of the downstream effects was investigated further by measuring oxygen utilization by live isolated muscle fibers from WT and *Eif6*^*+/−*^ mice, following the addition of ADP as a substrate (Figure 6A). There was a significant decrease in respiratory control ratio (RCR) of *Eif6*^*+/−*^ fibers compared with control, indicating impaired mitochondrial respiration efficiency in complexes III and IV of the electron transport chain (Figure 6B, p = 0.02), together with a trend toward an increase in the ADP:oxygen (P:O) ratio (Figure 6C, p = 0.07). These observations are consistent with a comparative reduction in oxygen utilization by the electron transport chain in mitochondria from *Eif6*^*+/−*^ muscle, while a reduced efficiency of respiration suggests an increase in reactive oxygen species (ROS) generation. Electrically stimulated contraction of cultured WT fibers increased superoxide ROS generation as expected (p < 0.01), whereas contracting *Eif6*^*+/−*^ fibers showed no detectable increase in superoxide generation (Figure 6D). However, this was accompanied by a significant increase in baseline generation of superoxide ROS in *Eif6*^*+/−*^ fibers compared to WT, such that unstimulated *Eif6*^*+/−*^ fibers were more similar to stimulated WT fibers (p *<* 0.01) (Figure 6D).

Investigating the causes of mitochondrial impairment in haploinsufficient mice, we found that while *Eif6*^*+/−*^ fibers have no change in copper-zinc superoxide dismutase (CuZn-SOD, SOD1) (Figure 6E), they have significantly higher level of manganese-superoxide dismutase (Mn-SOD, SOD2) compared to WT fibers (p < 0.04) (Figures 6F and S5). SOD2 catalyzes the conversion of superoxide generated by “proton leaks” from the electron transport chain into hydrogen peroxide and is essential for cellular health, as it reduces the oxidative damage resulting from ROS production. Hence, the observation of elevated levels of SOD2 in the mitochondria is consistent with the lack of detection of increased levels of superoxide and ROS observed following electrically stimulated contractions in *Eif6*^*+/−*^ muscle fibers.

### Haploinsufficient *Eif6*^*+/−*^ Mice Perform Less Efficiently than WT Mice in Exercise Tests

**Figure 7:**
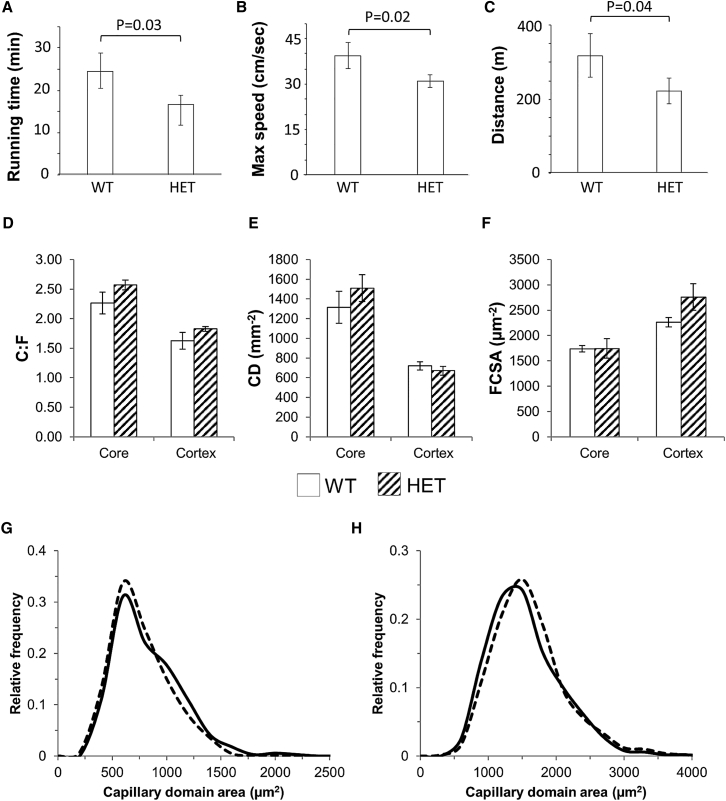
Exercise Capacity of *Eif6* Heterozygote Mice (A) Running time in minutes to exhaustion of WT and *Eif6*^*+/−*^ mice. (B) Maximum speed obtained (cm/s) by WT and *Eif6*^*+/−*^ mice. (C) Total distance traveled by WT and *Eif6*^*+/−*^ mice. (D–F) Capillary-to-fiber ratio (D), capillary density (E), and fiber-cross sectional area (FCSA) (F) in the oxidative core and glycolytic cortex of WT and *Eif6*^*+/−*^ skeletal muscle. Data are represented as mean ± SEM. (G and H) The spatial distribution of capillaries as an index of diffusive limitation for gaseous exchange is shown by the distribution of capillary supply area (domains) in the oxidative core (G) and cortex (H) for WT and *Eif6*^*+/−*^ skeletal muscle.

The molecular and imaging analysis of muscle fibers isolated from haploinsufficient *Eif6*^*+/−*^ mice shows that these are less efficient in utilizing oxygen for respiration and are accumulating ROS at a higher rate than WT mice. These observations, together with the prediction of our computational analysis of the HERITAGE gene expression profiling data suggest a causal link between *Eif6* and exercise performance. To test this hypothesis, we performed an exhaustion treadmill test. This demonstrated that indeed *Eif6*^*+/−*^ mice experience a reduction in exercise performance measured as running time (Figure 7A), maximum speed (Figure 7B) and distance (Figure 7C). Meanwhile, there was no difference in basal maximal oxygen uptake (VO_2_ max), respiratory exchange ratio (RER), or calorie intake monitored by indirect calorimetry in an independent cohort of mice. (Table S6).

Since it is still possible that muscle fiber efficiency may be influenced by changes in capillary density, we measured capillary-to-fiber ratio, capillary density, fiber cross-sectional area and distribution of capillary domain per area in WT and *Eif6*^*+/−*^ mice post-training. We discovered that none of these parameters are significantly different between WT and heterozygous mice (Figures 7D–7H). This demonstrates that the reduce efficiency of training is not the result of a difference in muscle blood supply but indeed a difference in fiber efficiency supporting the hypothesis of a causal link between *Eif6* and exercise performance.

## Discussion

Here, we show that endurance training involves a profound rewiring of gene regulatory networks, a phenomenon that is at least in part dependent on *Eif6*, an mTor-independent translation factor.

### Data-Driven Learning of Biological Networks Shows that a Wide Range of Known Homeostasis Pathways Are Rewired following Exercise Training

The network we developed represents a comprehensive view of signaling pathways, regulatory mechanisms, and their connection to effector functions known to be important in muscle plasticity. The signaling component of the model includes pathways that recapitulate known signaling events following the activation of the neuromuscular junction. These include calcium sensors (e.g., CALM3, CAMK2D), calcium-activated potassium channels (e.g., KCNN1, KCNN4), and other voltage-gated ion channels (e.g., KCNA1, KCNH2). The model also captures the connection between the calcium-activated kinase CAMK2D with the transcription factor MEF2A (Zhang et al., 2007), which is known to activate a program of gene expression in skeletal muscle controlling several metabolic and tissue remodeling pathways (Wu et al., 2000), including the structural components of the muscle fiber represented in the MEF2A-connected M7.

The ERBB signaling pathway, including neuregulin (NRG1), is also enriched in the signaling component of the model. The pathway is known to regulate differentiation and metabolic adaptation in response to contractile activity in skeletal muscle (Lebrasseur et al., 2003, Gumà et al., 2010). NRG1 has been shown to induce an oxidative phenotype and improve insulin sensitivity in muscle cells in a similar manner to endurance training (Cantó et al., 2007). Our network reveals a linkage between this pathway and modules representing mRNA processing, muscle fiber constituents and tissue-remodeling factors, supporting the hypothesis that ERBB signaling may control expression of effector function genes in the response to endurance exercise in humans.

The regulatory component of the model includes epigenetic and post-translational modulators (e.g., HDAC3 in M3). Protein deacetylases have been identified as key regulators of skeletal muscle metabolism, controlling mitochondrial function, fatty acid oxidation and ROS production (Gerhart-Hines et al., 2007, Jing et al., 2011, Sun et al., 2011).

We have shown that putative relationships between pathways can be revealed using a data-driven approach to infer and compartmentalize molecular networks in skeletal muscle. While our strategy is effective, it does not allow the inference of true directionality between individual molecules and pathways. Techniques such as dynamic Bayesian networks can overcome this challenge. However, owing to the complexity of large-scale probabilistic models, the sample sizes and computational resources required are currently prohibitive for networks containing large numbers of genes. The number of transcriptomics samples in the HERITAGE study is sufficient to support correlative approaches that produce indirect networks, but not methods that infer directionality.

### *Eif6*, Metabolic Remodeling, and Exercise Performance

The network analysis revealed *Eif6* as the most connected translation factor linked to energy metabolism. Further experimental validation supported the hypothesis that this linkage may be a causal one. Previous studies exploring the relationship between translation and energy metabolism have predominantly focused on the activity of the master regulator and nutrient sensor mTOR. Indeed, one of the most critical factors responsible for metabolic adaptation, PGC1α, has been shown to be downstream of mTOR (Cunningham et al., 2007). Importantly, since *Eif6* can act independently of mTOR (Ceci et al., 2003, Brina et al., 2015) and considering our results, we conclude that this may represent an alternative mTOR-independent pathway for metabolic adaptation.

We have shown that in liver and fat tissue *Eif6* controls lipid synthesis and glycolysis by stimulating translation of an upstream open reading frame (uORF) and G/C-rich-containing mRNAs (Brina et al., 2015). Here, we have confirmed the striking capability of *Eif6* to regulate metabolism in muscle. Similarities and differences with previous studies in liver, fat (Brina et al., 2015), and bone marrow (Ricciardi et al., 2015) have emerged. Briefly, *Eif6* depletion in muscle now confirms that in all insulin-responsive organs, the partial loss of *Eif6* is associated with a signature involved in modulated insulin sensitivity (Brina et al., 2015). In addition, *Eif6* levels seem to affect the general acetylation profile of metabolic proteins. However, two tissue-specific differences emerge: in muscle, and contrary to other tissue, lipid synthesis is not a primary target of *Eif6* regulation in muscle cells.

The alterations in metabolism, including decreased oxygen utilization efficiency and increased ROS production, may be responsible for the observed reduced performance in a treadmill exercise test in the *Eif6*^*+/−*^ mice. However, treadmill performance is not dictated solely by muscle performance. The effects of *Eif6* reduction in other tissues responsible for respiration and cardiovascular function, such as cardiac and lung, remain to be elucidated. The fact that mutant mice do not display any change in the fiber-type composition and blood supply strongly support the hypothesis that *Eif6* is causally linked to muscle fiber efficiency, both in energy production and in performance training.

### Relevance of *Eif6* in Pathogenesis

In conclusion, *Eif6* could have an important role to play in endurance training. *Eif6* is already considered a rate limiting step in transformation and has been shown to regulate entry to the cell cycle (Gandin et al., 2008). This raises the possibility that *Eif6* may play an important role in other contexts.

Interestingly, our network analysis revealed a correlation between *Eif6* expression and a marker of systemic inflammation, CRP. Alterations in the level of ROS, which we observe in *Eif6*^*+/−*^ muscle fibers, can upregulate expression of inflammatory genes including CRP (Wei et al., 2008). This raises the possibility that *Eif6* is important in chronic inflammatory diseases. We found evidence from several mouse and human studies that *Eif6* mRNA levels were significantly altered in diverse pathological and physiological conditions. This included diseases such as Alzheimer’s disease, which exhibited a significant decrease in *Eif6* expression in neuronal cells (Figure S6A). Alzheimer’s disease is known to involve extensive metabolic changes and ROS induced damage (Dumont and Beal, 2011). In mice, *Eif6* expression was significantly upregulated in response to a combination of a high-fat diet and metformin, a drug commonly used to treat diabetes (Setter et al., 2003) (Figure S6B). Skeletal muscle denervation and innervation, which strongly affect mitochondrial function (O’Leary et al., 2012), including ROS production (Muller et al., 2007), also profoundly altered the expression of *Eif6* in mice (Figure S6C), strongly supporting an important role of *Eif6* in muscle plasticity. Together, these support an interesting possibility that *Eif6* may be involved in a wide spectrum of diseases.

## Experimental Procedures

### HERITAGE Dataset Gene Expression Analysis

The experimental design and exercise training protocol of the HERITAGE Family Study have been described previously (Bouchard et al., 1995). Participants were sedentary at baseline and normotensive. Each participant exercised three times per week for 20 weeks on cycle ergometers controlled by direct heart rate (HR) monitoring. Briefly, participants exercised at the HR associated with 55% of baseline VO_2_ max for 30 min per session for the first 2 weeks. The duration and intensity were gradually increased every 2 weeks, until reaching 50 min and 75% of the HR associated with baseline VO_2_ max. This level was maintained for the final 6 weeks of training.

Muscle biopsies of *vastus lateralis* were obtained at baseline and post-training using the percutaneous needle biopsy technique. Total RNA was isolated from frozen muscle biopsies preserved in Tissue-Tek using Trizol, and mRNA was amplified with Ambion MessageAmp Premier following the manufacturer’s instructions as previously described (Phillips et al., 2013). Affymetrix HG-U133+2 arrays were used to measure global gene expression levels in baseline and in post-training samples. After removing participants for whom either pre- or post-training arrays failed quality control procedures or were not generated, 41 participants remained. The biopsies were performed between 1994 and 1997. The Affymetrix microarrays we performed in 2012 and all samples were profiled at the same time. No sign of bias or batch effects were detected using cluster analysis, principal component analysis, and other data exploration approaches. Genes differentially expressed in response to training were identified using a paired-design two-class significance analysis of microarrays (SAM) (Tusher et al., 2001) with a FDR threshold of 5%.

### Differential Co-expression Networks

To identify genes that showed alterations in correlation patterns (differential co-expression) between trained and untrained subjects, we applied a slightly modified pipeline based on the DiffCoEx procedure (Tesson et al., 2010). DiffCoEx is designed to identify correlation pattern changes using the weighted gene co-expression network analysis (WGCNA) (Langfelder and Horvath, 2008) framework. Briefly, two datasets were defined (pre and post-training), followed by gene cluster identification based on the topological overlap or similarity of differential co-expression between the datasets (Langfelder et al., 2008). We then applied a filter to each gene cluster to remove non-significant changes in gene-gene correlation. A resampling procedure was used to generate two datasets of randomly permuted data. This was then used to calculate a null distribution of co-expression changes, which, in turn, was used to calculate a p value for each gene-gene interaction. Differential co-expression changes with an FDR > 1% were then removed. The standard DiffCoEx resampling procedure was then performed using the filtered clusters. This involved constructing 1,000 sets of random clusters of the same distribution of sizes to build a null distribution of module-module dispersion statistics (a measure of the module-module differential co-expression). Module-module connections with an FDR < 10% were selected as edges within the network. All modules were significant for within-module changes in co-expression.

### Data Integration

The HERITAGE gene expression data were used to infer a network-integrating eIF gene expression profiles, KEGG pathways, and physiological measurements (Table S1). Genes differentially correlated to eIF genes between trained and untrained individuals were linked to KEGG pathways using a gene set enrichment analysis (GSEA) approach (Subramanian et al., 2005). Over-represented KEGG pathways (FDR < 10%) were visualized in the network as an edge between the eIF gene and the KEGG pathway.

EIF genes were linked to physiological measurements using linear regression. A simple model of the effect of training on the physiological variable as a function of pre-training gene expression levels was used to generate p values for each relationship. Significant relationships (FDR < 10%) were visualized in the same manner as KEGG pathways.

### Gene Ontology Analysis

The R library clusterProfiler (Yu et al., 2012) was used to test for functional enrichment of gene ontology and KEGG pathway terms within gene lists. A term was considered significantly enriched at a FDR less than 10%.

### Animal Studies

All animal work was conducted according to relevant national and international guidelines and approved by the University of Birmingham, UK, Medical School and University of Liverpool, UK, Medical Services Unit ethics committee and by the responsible authority of the Regierung von Oberbayern, Germany. Young (8- to 16-week old) male *Eif6*^*+/−*^ or *Eif6*^*+/+*^ C57BL6 littermates were sacrificed by cervical dislocation, and skeletal muscle was immediately removed, cleaned of any excess fat or connective tissue, and either flash frozen in liquid nitrogen or placed in the relevant medium for immediate use. Tissue placed in storage was held at −80°C until further use.

### Statistical Analysis

We used unpaired Student’s t tests for two comparison groups unless stated otherwise, where N represents the number of replicates in each group. Where multiple tests were carried out, p values were corrected using the FDR of Benjamini and Hochberg (1995).

### Indirect Calorimetry

To evaluate energy metabolism under baseline conditions, a 21-hr indirect calorimetry trial was conducted in single caged mice at the age of 12 weeks having free access to food and water (32-cage PhenoMaster with activity and drinking/feeding monitoring, TSE Systems, Bad Homburg, Germany). Data were collected in 20-min intervals, resulting in a total of 63 time points per 21 hr. The following variables were assessed: oxygen consumption (VO_2_), carbon dioxide production (VCO_2_), the respiratory exchange ratio (VCO_2_/VO_2_), food consumption, locomotor activity (distance traveled in cm / 20 min) and rearing behavior (counts/20 min), body weight, and rectal body temperature. All VO_2_ data were used to calculate mean oxygen consumption, and lowest and highest single readings were identified as minimum and maximum VO_2_ under baseline conditions. Oxygen consumption data were analyzed using linear regression modeling, including body mass as a co-variate to normalize for body mass differences.

### Treadmill Test

Two groups of mice (WT, n = 9; Eif6 het, n = 8; age- and sex-matched) were subjected to an exhaustion treadmill test. Each mouse was placed on the belt of a 6-lane motorized treadmill supplied with shocker plates. The treadmill was run at an inclination of +5°. The speed was initially 15 cm/s and then increased by 2 cm/s every 2 min using a sight electric stimulation of 0.3 mA. The test was stopped when the mouse remained on the shocker plate for more than 5 s without attempting to re-engage the treadmill, and the time to exhaustion was determined.

### Immunochemistry

Cross-sections (10–12 μm) were cut from the mid-belly of the quadriceps, *gastrocnemius*, and *soleus* muscles on a Bright OTF5000 cryostat (Bright Instrument, England) at −22°C, air-dried, and stored at −80°C prior to further processing. Following thawing at room temperature and to distinguish type 1, 2A, and 2B/X muscle fibers, cross-sections were stained for ATPase after pre-incubation at pH 7.0 as previously described (Tunell and Hart, 1977). From a region of the cross-section containing more than 1 fiber type, an average of 270 and 210 fibers were counted for each WT and *Eif6*^*+/−*^, respectively. Data are expressed as mean ± SD for n = 4 WT and n = 4 *Eif6*^*+/−*^ animals.

Capillaries were labeled with fluorescently tagged lectin I (GSL I, Vector Labs). Digitized images of stained sections were used to determine the x and y coordinates for muscle fiber boundaries and the associated capillary coordinates; in-house software was used to calculate the capillary-to-fiber ratio (C:F), capillary density (CD, mm^2^), and mean fiber cross-sectional area (FCSA, μm^2^). The area of tissue supplied by individual capillaries (the domain of influence) was calculated as a tessellation of non-overlapping polygons, representing the area of tissue closer to one capillary than any other. Under conditions of maximal flow, assuming supply capacity to be similar for all capillaries, the domain size will be inversely proportional to the metabolic demand. The distribution of capillary domain area provides an index of the heterogeneity of capillary supply. The log-normal distribution is best expressed as the standard deviation of the log-transformed area (logSD) (Al-Shammari et al., 2014).

### Western Blotting

For whole-muscle western blot analysis, approximately 50 mg *gastrocnemius* muscle was ground to a powder using a mortar and pestle in the presence of liquid nitrogen. Thereafter, the powder was resuspended in an amount of RIPA buffer (Sigma), homogenized for 60 s, and then centrifuged at 7,000 × *g* for 5 min. The supernatant was collected and protein concentration determined using the BradfordUltra method.

Samples were diluted in Laemmli buffer to yield between 20–60 μg of protein per sample that were separated by a 12% SDS-PAGE with proteins subsequently being transferred to nitrocellulose and thereafter probed against Cu/Zn SOD (SOD1) (Enzo), MnSOD (SOD2) (Abcam), NDUFS4 (Abcam), ENO3 (Abcam), and anti-eIF6 (produced in-house).

### qPCR

Mitochondrial number was estimated using a real-time PCR-based method. Briefly, total DNA was isolated from a muscle sample (2–5 mg) using the DNeasy Kit (QIAGEN, USA). Nuclear DNA (nuclear DNA) was quantified using primers for glyceraldehyde 3-phosphate dehydrogenase (*Gapdh*); forward 5′CCCACTAACATCAAATGGGG3′ and reverse 5′TCTCCATGGTGGTGAAGACA3′ (amplicon 76). mtDNA was quantified using primers for the mitochondrial encoded cytochrome b; forward 5′CCACTTCATCTTACCATTTATTATCGC3′ and reverse 5′TTTTATCTGCATCTGAGTTTGATCCTGT3′ (amplicon 110). Separate reactions for *Gapdh* and cytochrome b were set up using approximately 12.5 ng of total DNA using the SensiMix Sybr Green Master Mix (Bioline, UK). Real-time PCR was performed using a Bio-Rad iCycler with an iCycler iQ multicolor real-time PCR detection system (Bio-Rad, USA) over 26 cycles and a 62°C annealing temperature. The ratio of nuclear DNA:mtDNA was calculated from the cycle threshold (CT) values and compared between WT and *Eif6*^*+/−*^ mice.

### Microarray Analysis of *Eif6*^*+/−*^ Muscle

Frozen *soleus*, *gastrocnemius*, and *tibialis anterior* muscle was homogenized in RLT buffer (QIAGEN) with β-mercaptoethanol (1% v/v) using a Precellys-24 tissue homogenizer system (Precellys, UK). Each tissue lysate was centrifuged at 13,000 rpm for 10 min, and the supernatant was removed for RNA extraction. RNA was isolated using QIAGEN RNeasy columns following the manufacturer’s instructions. Sample purity was assessed by measuring absorbance at 260 and 280 nm using a NanoDrop spectrophotometer, and all absorbance ratios were between 1.8 and 2.0. Cy3-labeled cRNA was generated using the Agilent Low-Input QuickAmp Kit following the manufacturer’s instructions. 600 ng of labeled cRNA was hybridized to Agilent Sureprint G3 Mouse whole-genome microarrays, which were then washed and scanned in an Agilent SureScan microarray scanner. Microarray data were log_2_ transformed and normalized using quantile normalization. Differentially expressed genes were detected using SAM with a 5% FDR cutoff.

### Polysome Purification and Polysomal mRNA analysis

The polysome analysis followed a modified protocol from Kubica et al. (2005). For a full description of the protocol, see the Supplemental Experimental Procedures. Briefly, 180 μg of muscle lysate was layered on a 15%–50% linear sucrose gradient and centrifuged at 37,000 rpm for 170 min. The sucrose gradient was fractionated and UV absorption at 260 nm was recorded. RNA was precipitated by adding 2 volumes of 100% ethanol, redissolved in RNase-free water, and treated with phenol-chloroform. Sucrose fractions containing ≥2 polysomes were pooled for subsequent analysis. Total RNA was extracted from muscle using the methods of Caddick et al. (2006). The mRNA was then processed for microarray analysis as described above. Statistically significant genes were selected using an absolute log_2_-fold change threshold greater than 1 and p < 0.05.

### Mitochondrial Proteomics

For a full description of the protocol, see the Supplemental Experimental Procedures. Briefly, mitochondria were isolated from fresh *gastrocnemius* muscle of WT and *Eif6*^*+/−*^ mice as previously described (Wittig et al., 2007). Following digestion and liquid chromatography (LC) separation, samples were analyzed on a Quadrupole-Orbitrap instrument in data-dependent positive (ESI+) mode to automatically switch between full-scan mass spectrometry (MS) and MS/MS acquisition. Raw data files were uploaded into Proteome Discoverer (v.1.3) and searched against the mouse UniProt database using the Mascot search engine (v.2.4.1). The threshold for detected proteins was adjusted to a FDR of less than 5% and imported into Progenesis. Differentially expressed proteins were detected using ANOVA, followed by adjustment to a FDR of 10%.

The enrichment of differentially expressed proteins within the polysomal fraction of highly translated mRNAs in *Eif6*^*+/−*^ muscle was determined using GSEA. Proteins within the “core enriched” fraction of differentially expressed proteins were considered overlapping between the two experiments (Table S4).

### Acetylome Analysis

For a full description of the method, see the Supplemental Experimental Procedures. Total protein from *gastrocnemius* muscle was extracted (3 WT and 3 *Eif6*^*+/−*^ mice) following the method of Lambertucci et al. (2012). Pre-cleaned protein lysates were rotated overnight at 4°C with protein-G-conjugated anti-acetyl lysine antibody. Anti-acetyl lysine immunoprecipitated proteins from WT or *Eif6*^*+/−*^ mice were labeled using the TMT Sixplex Isobaric Mass Tagging Kit (Thermo Scientific) following the manufacturer’s instructions. Samples were analyzed using an Orbitrap Velos ETD mass spectrometer (Thermo Scientific) and Proteome Discoverer (v.1.3) software.

### Oxytherm Analysis

*Gastrocnemius* muscle from WT and *Eif6*^*+/−*^ mice was dissected and immediately placed in tissue culture media on ice. Oxygen consumption was measured using a Clark-type oxygen electrode (Hansatech, UK). All measurements were completed at 30°C using 20-mg muscle fiber bundles permeabilized using saponin (50 μg/ml) in ROS buffer (pH 7, in millimolars: 1 MgCl_2_, 10 imidazole, 2 EGTA, 100 KCl, and 10 KH_2_PO_4_). Muscle bundles were then washed three times to remove saponin. Respiration was initiated in fresh ROS buffer by the addition of 5 μM glutamate/malate (GM) in the absence (state 4) and in the presence (state 3) of 500 μM ADP. Respiratory control ratios were calculated as state 3 divided by state 4 respiration rates. P:O ratio was calculated as the ratio of the amount of nucleotide added and molecular oxygen consumed during state 3 respiration.

### Muscle Fiber Extraction

Single fibers were isolated from the *flexor digitorum brevis* (FDB) muscle of mice as previously described (Pearson et al., 2014). Muscles were incubated for 1.5 hr at 37°C in 0.4% (w/v) sterile type 1 collagenase (EC 3.4.24.3, Sigma Chemical, UK) in minimum essential medium eagle (MEM) containing 2 mM glutamine, 50 IU penicillin, 50 μg/ml streptomycin, and 10% fetal bovine serum (FBS) (Sigma Chemical). The muscles were agitated every 30 min during the digestion period to release single fibers and thereafter were washed three times in MEM containing 10% FBS. Fibers were plated onto pre-cooled 35-mm glass-bottomed cell culture dishes (MatTek, MA, USA) pre-coated with Matrigel (BD Biosciences, Oxford, UK) and were allowed to attach.

### ROS Imaging

For a full description of the method, see the Supplemental Experimental Procedures. Briefly, fibers were incubated in 2 mL D-PBS containing 250 nM MitoSox Red or 10 μM DAF-FM DA for 30 min at 37°C. FDB fiber contraction was induced using platinum electrodes using field stimulations at 5–10, 20–25, and 35–40 min over a 50-min protocol. Images were collected at 5-min intervals using a Nikon E-Ti inverted microscope (TI-S-EJOY, Nikon). Data were tested by general linear models with repeated measures (examining stimulation and genotype) or Student’s t test as indicated.

## Author Contributions

Conceptualization, K.C. and F.F.; Methodology, K.C.; Formal Analysis, K.C., S.R., T.P., P.K.D., D.M.S., F.K., J.A., C.S., and F.F.; Investigation, K.C., S.R., T.P., I.B., M.B., J.R., D.B., A.S., D.M.S., F.K., J.A., M.A.S., S.G., A.P., C.S., and S.E.; Resources, S.B., D.B., and M.H.d.A.; Writing – Original Draft, K.C., J.A., and F.F.; Writing – Review & Editing, K.C., S.R., T.P., P.K.D., D.M.S., R.J.B., M.A.S., S.J., A.P., S.E., M.C., S.B., and F.F.; Visualization, K.C., S.R., T.P., C.S., and F.F.; Supervision, R.J.B., C. Bunce, C.S., S.E., M.C., M.J., C. Bouchard, S.B., and F.F.

## Data and Software Availability

The accession number for the gene expression data reported in this study is GEO: GSE47874.

## Supplemental Information

Document S1. Supplemental Experimental Procedures, Figures S1–S6, and Tables S1–S6

Table S7. Differentially Expressed Proteins in the Eif6 Mitochondrial Proteome, Related to Figure 4

Table S8. Statistical Analysis of Eif6 Polysomal Microarray Data Compared to WT, Related to Figure 4

Document S2. Article plus Supplemental Information
