# Supplementary material for: The Role of Eif6 in Skeletal Muscle Homeostasis Revealed by Endurance Training Co-expression Networks
Source: Cell Rep. 2017 Nov 7;21(6):1507–20. doi: 10.1016/j.celrep.2017.10.040 (PMC5695912; doi:10.1016/j.celrep.2017.10.040)
Supplement: Document S1. Supplemental Experimental Procedures, Figures S1–S6, and Tables S1–S6 [file mmc1.pdf]

Cell Reports, Volume *21*

Supplemental Information

The Role of *Eif6* in Skeletal Muscle Homeostasis Revealed by Endurance Training Co-expression Networks

Kim Clarke, Sara Ricciardi, Tim Pearson, Izwan Bharudin, Peter K. Davidsen, Michela Bonomo, Daniela Brina, Alessandra Scagliola, Deborah M. Simpson, Robert J. Beynon, Farhat Khanim, John Ankers, Mark A. Sarzynski, Sujoy Ghosh, Addolorata Pisconti, Jan Rozman, Martin Hrabe de Angelis, Chris Bunce, Claire Stewart, Stuart Egginton, Mark Caddick, Malcolm Jackson, Claude Bouchard, Stefano Biffo, and Francesco Falciani

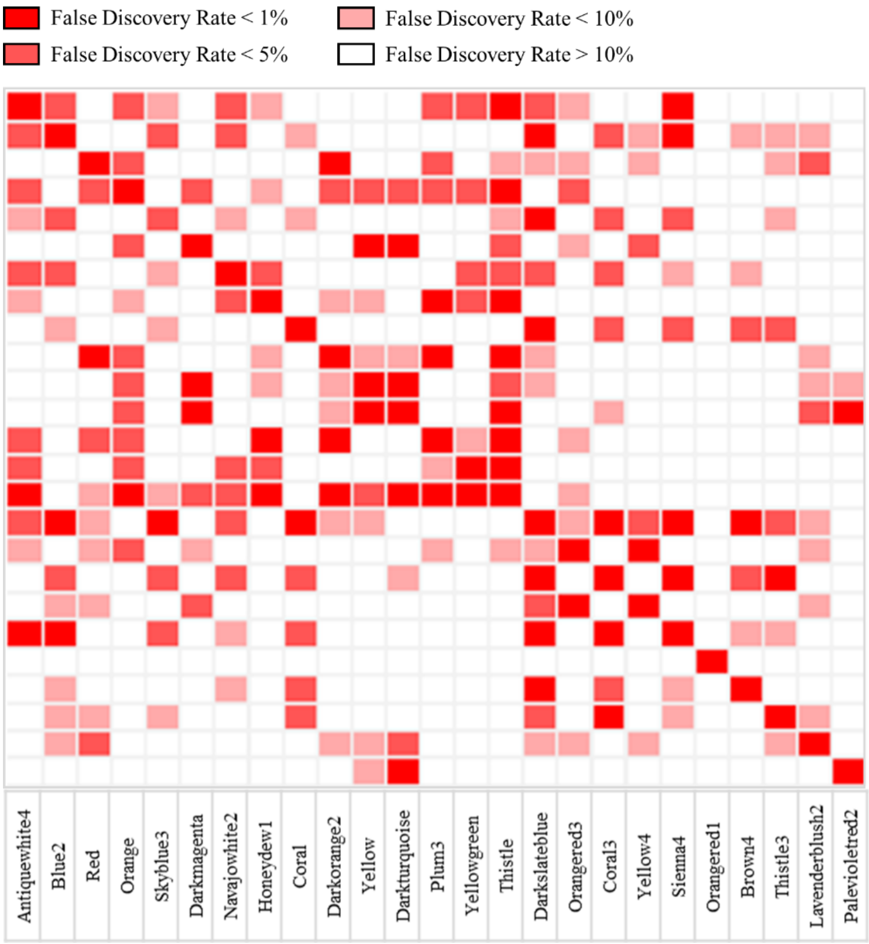

Module Label (DiffCoEx)	Module Size	Module Label (Fig 1)	Enrichment of D.E.G (endurancetraining)
Antiquewhite4	2989	M1	
Blue2	302	M7	
Red	545	M6	
Orange	424	M5	
Skyblue3	1955	M8	
Darkmagenta	660	M3	↓
Navajowhite2	898	M4	
Honeydew1	31		
Coral	447	M9	↓
Darkorange2	175	M10	↓
Yellow	101	M2	
Darkturquoise	68		
Plum3	18		
Yellowgreen	40		
Thistle	18		
Darkslateblue	40		
Orangered3	22		
Coral3	13		
Yellow4	28		
Sienna4	19		
Orangered1	7		
Brown4	42		
Thistle3	21		
Lavenderblush2	16		
Palevioletred2	17		

Fig S1 – Results of the DiffCoEx module-to-module resampling procedure. Related to Figure 1.
The DiffCoEx procedure includes a resampling procedure to ascertain the significance of module-wise changes in co-expression between two conditions, assessed using the dispersion statistic. This heatmap represents the results of that resampling procedure, represented as a false discovery rate for each pairwise module-module test, as well as the module sizes (number of genes), module labels and enrichment of genes differentially expressed in response to training.

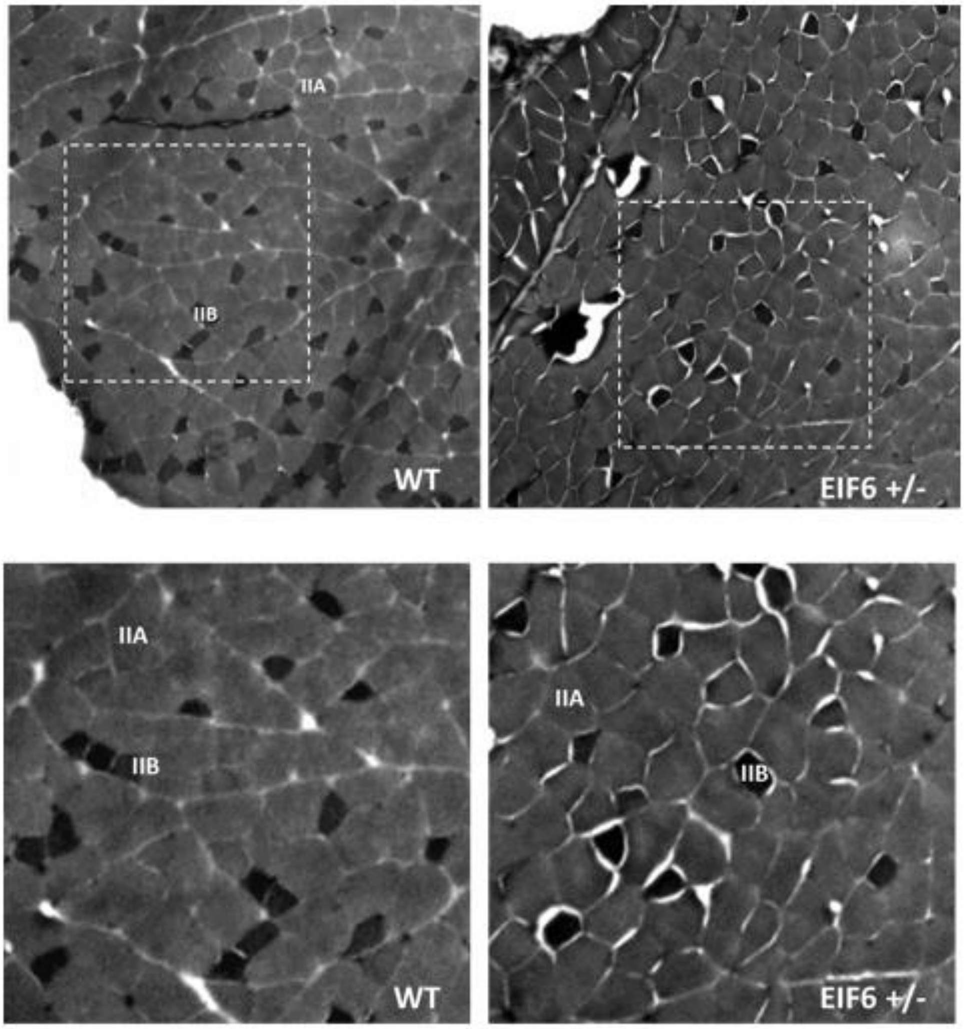

A

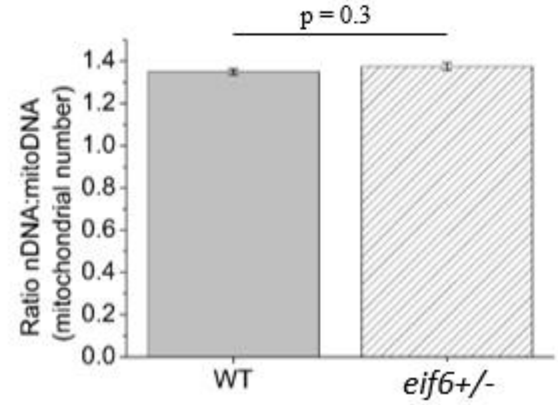

B

Fig S2 – Fibre type staining and mtDNA/nDNA ratio in *eif6*$^{+/-}$ skeletal muscle. Related to Figure 3.
(A) Representative images of type IIA and IIB fibre type staining in skeletal muscle sections from wild-type and *eif6*$^{+/-}$ mice. (B) Ratio of mitochondrial to nuclear DNA (cytB/Gapdh) in wild-type and haploinsufficient skeletal muscle. Data shown is mean +/- SEM. P-value calculated using the Students T-test. Related to Figure 4.

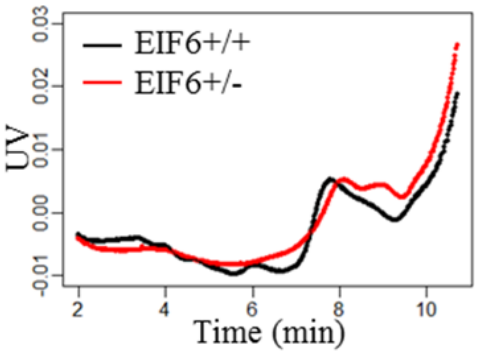

Fig S3 - Representative polysome trace from eIF6 heterozygote and wild type skeletal muscle. Relates to Figure 4.

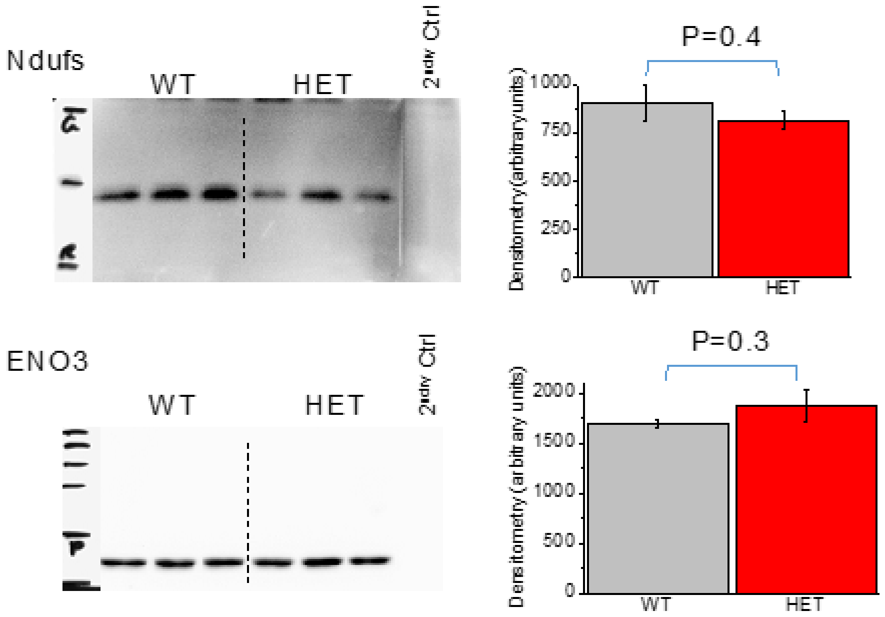

Fig S4 - Western blot analysis of NDUFS4 and ENO3 protein in wild type and *eif6*$^{+/-}$ skeletal muscle. Relates to Figure 5.

Barplots represent mean signal +/- SEM. P-values were calculated using the Students T-test.

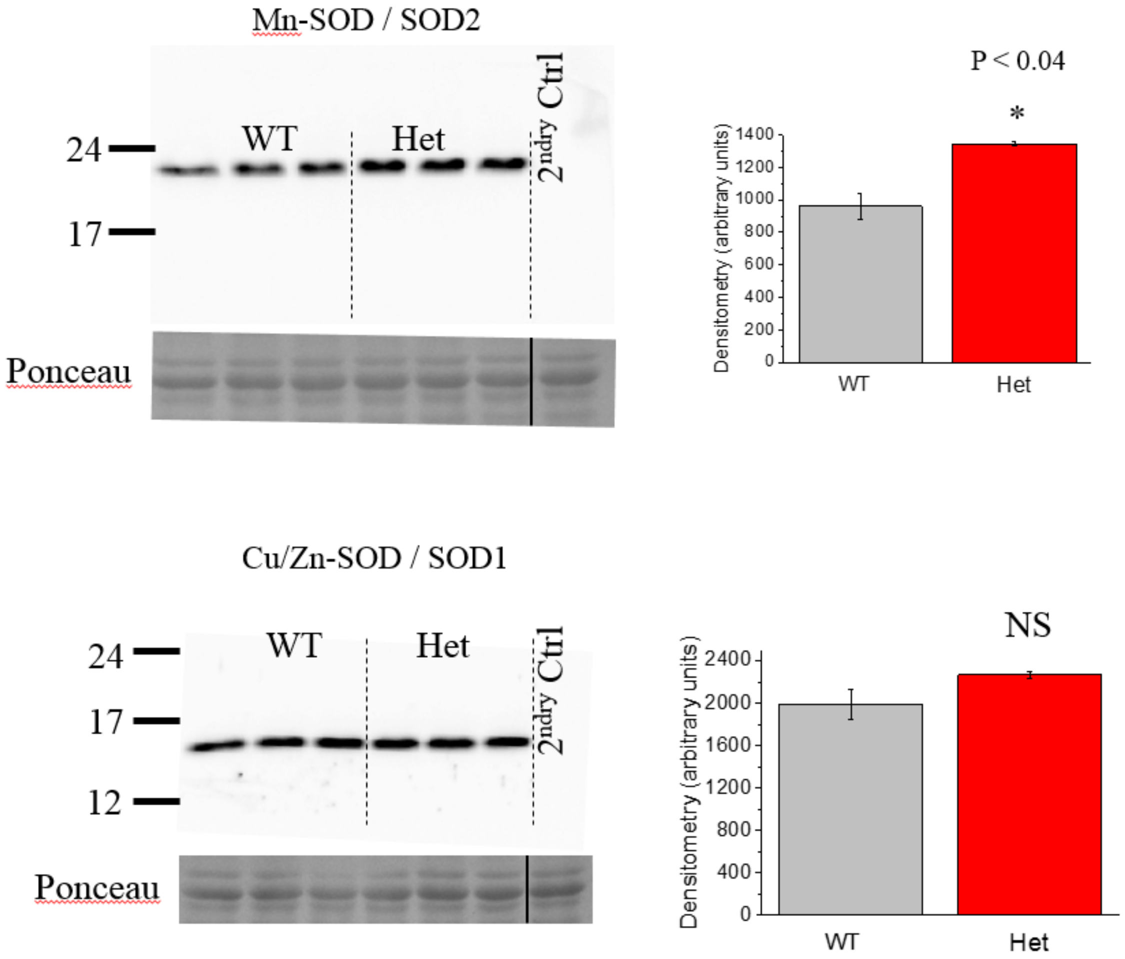

Fig S5 – Western blot analysis of SOD1 and SOD2 protein in wild type and *eif6*$^{+/-}$ skeletal muscle. Relates to Figure 6.

Barplots represent mean signal +/- SEM. P-values were calculated using the Students T-test.

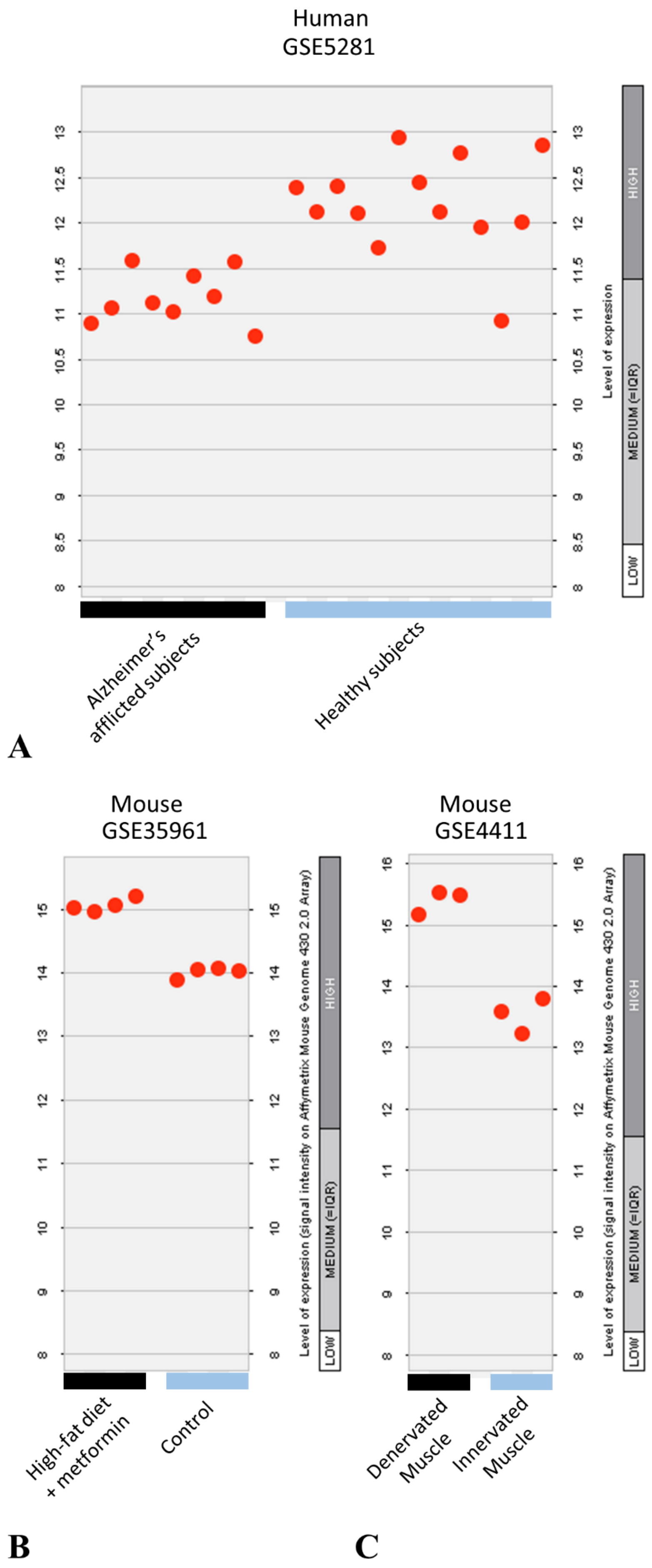
Human
GSE5281
Level of expression
HIGH
MEDIUM (=IQR)
LOW
Alzheimer's afflicted subjects
Healthy subjects
A
Mouse
GSE35961
Level of expression (signal intensity on Affymetrix Mouse Genome 430 2.0 Array)
High-fat diet + metformin
Control
B
Mouse
GSE4411
Denervated Muscle
Innervated Muscle
C

Fig S6 - Differential expression of eIF6 in existing datasets from GeneVestigator, relates to Figure 2.
(A) Cortical layer III pyramidal neurons of the posterior cingulate of clinically and neuropathologically classified late-onset Alzheimer´s disease afflicted individuals or neurologically normal individuals (healthy subjects). Log_2 ratio -1.04. $P < 0.001$
(B) Liver tissue samples obtained from C57BL/6 8 weeks old mice fed with high fat (HF) diet which was methionine- and choline-deficient (MCD) and contained 0.1% metformin (37.5mg/kg) vs regular chow. Log_2 ratio 1.06. $P < 0.001$
(C) Denervated vs innervated tibialis anterior muscles derived from runx f/f control mice. Log_2 Ratio 1.85. $P < 0.001$

Table S1 – Physiological and biochemical measurements accompanying the HERITAGE gene expression cohort, related to Figure 2

Measurement	Description	Units	Mean Pre-training	Standard Deviation Pre-training	Mean Post-training	Standard Deviation Post-training	Trained vs untrained t-test P value
AIRg	Acute insulin response to glucose	pmol/L	680.94	513.59	613.50	386.41	4.87E-01
CK_enz	Creatine kinase	U/g	396.25	69.42	422.25	65.59	1.83E-03
COX_enz	Cyclooxygenase	U/g	7.00	1.63	10.11	2.25	3.72E-11
CPT_enz	Carnitine palmitoyltransferase	U/g	0.12	0.02	0.16	0.02	2.88E-16
CRP	C-reactive protein	mg/L	0.17	0.22	0.23	0.25	4.31E-02
CS_enz	Citrate synthase	U/g	11.51	2.18	17.25	3.55	9.33E-18
fastGlu_BP	Fasting blood glucose	mmol/L	5.10	0.56	5.16	0.58	2.78E-01
fastIns_BP	Fasting blood insulin		62.59	32.73	59.18	25.04	4.06E-01
GAPDH_enz	Glyceraldehyde 3-phosphate dehydrogenase	U/g	429.05	100.21	431.23	85.00	1.35E-01
HADH_enz	3-hydroxyacyl-CoA dehydrogenase	U/g	16.57	2.97	20.95	3.85	4.22E-13
HDL.chol_BP	HDL cholesterol	mmol/L	1.06	0.24	1.15	0.25	3.10E-04
HK_enz	Hexokinase	U/g	2.59	0.50	3.08	0.59	6.39E-07
LDL.chol_BP	LDL cholesterol	mmol/L	2.83	0.81	2.91	0.83	2.90E-01
Leptin_BP	Blood leptin	ng/mL	11.04	8.41	10.09	9.65	2.72E-01
lipoprot_lipase_IM	Lipoprotein lipase (intramuscular)	umol FFA/g/h	0.41	0.17	0.42	0.18	8.60E-01
lipoprot_lipase_released	Lipoprotein lipase	umol FFA/g/h	0.48	0.24	0.45	0.18	3.53E-01
logFFMI	Logged fat free mass index	kg/m2	1.28	0.11	1.28	0.10	9.85E-02
logISI	Logged insulin sensitivity index	mU/L/min	0.56	0.47	0.60	0.25	7.58E-01
PHO_enz	Glycogen phosphorylase	U/g	20.97	4.64	22.60	4.51	5.44E-06
PhoFK_enz	Phosphofructokinase	U/g	58.45	13.25	62.56	14.27	1.05E-03
TG_BP	Blood triglycerides	mmol/L	1.32	0.77	1.25	0.64	1.55E-01
UCP2_IM	Mitochondrial uncoupling protein 2	A.U	36.42	7.78	39.49	6.90	1.42E-03
VO2max	Peak oxygen uptake or maximal aerobic capacity	L O2/min	2.73	0.81	3.11	0.86	5.57E-15

Table S2 – Skeletal muscle fibre type counts in *eif6*$^{+/-}$ and wild type mice, relates to Figure 3
Data representive of over 1,700 fibres per muscle type, n=4 mice per group

**WT SOLEUS muscle**	**%TI**	**%TIIA**	**%TIIB**
**Average**	29.60	68.62	1.78
**STD DEV**	4.70	4.31	0.40
**HET SOLEUS muscle**	**%TI**	**%TIIA**	**%TIIB**
**Average**	33.44	64.54	2.03
**STD DEV**	2.40	3.48	1.48
**P-value**	0.2232	0.2252	0.806
**WT QUAD muscle**	**%TI**	**%TIIA**	**%TIIB**
**Average**	0.00	16.45	83.55
**STD DEV**	0.00	4.18	4.18
**HET QUAD muscle**	**%TI**	**%TIIA**	**%TIIB**
**Average**	0.00	16.51	83.49
**STD DEV**	0.00	1.60	1.60
**P-value**	1	0.98	0.98

Table S3 - Histone deacetylase genes linked to Eif6 modulation in human correlation networks, gene expression studies and polysomal loading studies, related to Figure 3

	Positive correlation with Eif6 expression	Negative correlation with Eif6 expression
Eif6 correlation network (human)	SIRT6, HDAC1, HDAC10	none

	Up-regulated	Down-regulated
Gene expression (Gastrocnemius)	Hdac1, Hdac6, Hdac7, Hdac9, Sirt1, Sirt6	Sirt2

	Increased loading in Eif6+/-	Increased loading in WT
Polysome loading	Sirt4, Sirt5	Sirt6

Table S4 - Differentialy expressed proteins enriched within the highly translated mRNAs in Eif6 heterozygote muscle, related to Figure 4

Gene	Gene Name
Ampd1	adenosine monophosphate deaminase 1(Ampd1)
Apoo	apolipoprotein O(Apoo)
Asph	aspartate-beta-hydroxylase(Asph)
Atp2a1	ATPase, Ca++ transporting, cardiac muscle, fast twitch 1(Atp2a1)
Bsg	basigin(Bsg)
Casq1	calsequestrin 1(Casq1)
Cat	catalase(Cat)
Cbr2	carbonyl reductase 2(Cbr2)
Eef1a2	eukaryotic translation elongation factor 1 alpha 2(Eef1a2)
Epdr1	ependymin related protein 1 (zebrafish)(Epdr1)
Gm5451	predicted gene 5451(Gm5451)
Mrps2	mitochondrial ribosomal protein S2(Mrps2)
Obscn	obscurin, cytoskeletal calmodulin and titin-interacting RhoGEF(Obscn)
Rac1	RAS-related C3 botulinum substrate 1(Rac1)
Rpl18a	ribosomal protein L18A(Rpl18a)
Rpl3l	ribosomal protein L3-like(Rpl3l)
Sypl2	synaptophysin-like 2(Sypl2)
Tmem109	transmembrane protein 109(Tmem109)
Tpp1	tripeptidyl peptidase I(Tpp1)
Acot13	acyl-CoA thioesterase 13(Acot13)
Acp6	acid phosphatase 6, lysophosphatidic(Acp6)
Aldh4a1	aldehyde dehydrogenase 4 family, member A1(Aldh4a1)
Coq7	demethyl-Q 7(Coq7)
Cox15	cytochrome c oxidase assembly protein 15(Cox15)
Cpox	coproporphyrinogen oxidase(Cpox)
Dap3	death associated protein 3(Dap3)
Fundc2	FUN14 domain containing 2(Fundc2)
Gfm2	G elongation factor, mitochondrial 2(Gfm2)
Gls	glutaminase(Gls)
Guf1	GUF1 homolog, GTPase(Guf1)
Hibch	3-hydroxyisobutyryl-Coenzyme A hydrolase(Hibch)
Iars2	isoleucine-tRNA synthetase 2, mitochondrial(Iars2)
Mrps22	mitochondrial ribosomal protein S22(Mrps22)
Mylpf	myosin light chain, phosphorylatable, fast skeletal muscle(Mylpf)
Oat	ornithine aminotransferase(Oat)
Ociad2	OCIA domain containing 2(Ociad2)
Pmpca	peptidase (mitochondrial processing) alpha(Pmpca)
Qrsl1	glutaminyl-tRNA synthase (glutamine-hydrolyzing)-like 1(Qrsl1)
Sdhd	succinate dehydrogenase complex, subunit D, integral membrane protein(Sdhd)
Sucla2	succinate-Coenzyme A ligase, ADP-forming, beta subunit(Sucla2)
Tnnt3	troponin T3, skeletal, fast(Tnnt3)

Table S5 – Significant changes in protein acetylation status in eif6+/- skeletal muscle, related to Figure 5

Gene Symbol	Description	Ratio WT/Het	Replicates
Electron transport chain - Complex 1			
NDUFS1	NADH-ubiquinone oxidoreductase 75 kDa subunit, mitochondrial	2.634	2
NDUFS8	NADH dehydrogenase [ubiquinone] iron-sulfur protein 8, mitochondrial	2.036	2
NDUFS4	NADH dehydrogenase [ubiquinone] iron-sulfur protein 4, mitochondrial	2.007	3
NDUFV1	NADH dehydrogenase [ubiquinone] flavoprotein 1, mitochondrial	1.965	2
Electron transport chain - Complex 3			
UQCRB	Cytochrome b-c1 complex subunit 7	2.243	2
UQCRC1	Cytochrome b-c1 complex subunit 1, mitochondrial	2.382	2
TCA Cycle			
MDH2	Malate dehydrogenase, mitochondrial	0.5	2
Pyruvate Metabolism			
LDHA	L-lactate dehydrogenase A chain	0.579	3
Glycolysis			
ENO3	Beta-enolase 3	0.486	3
GAPDH	Glyceraldehyde-3-phosphate dehydrogenase	0.735	2
TPI1	Triosephosphate isomerase	0.464	3
ALDOA	Fructose-bisphosphate aldolase A	0.512	3
Mitochondrial transport			
VDAC3	Voltage-dependent anion-selective channel protein 3	0.452	3
Amino acid metabolism			
GOT1	Aspartate aminotransferase, cytoplasmic	0.388	3
Muscle energy homeostasis			
CKM	Creatine kinase M-type	0.52	2
Other			
PDLIM5	PDZ and LIM domain protein 5 (Fragment)	0.695	2
MYLPF	Myosin regulatory light chain 2, skeletal muscle isoform	0.569	3
PVALB	Parvalbumin alpha	0.403	2
CYCS	Cytochrome c	0.643	3

Table S6 - Indirect calorimetry and basal respiration measurements of wild-type and Eif6 heterozygote mice, related to Figure 7

	Female				Male						
	Control		Mutant		Control		Mutant		Linear model -Sex	Linear Model - Genotype	Linear Model - Sex:Genotype Interaction
Category	Mean	SD	Mean	SD	Mean	SD	Mean	SD	P Value	P Value	P Value
Body Temp [°C]	36.94	0.68	37.1	0.64	36.769	0.37	36.71	0.43	0.16	**0.764**	0.6
Food Intake [g]	3.9	0.4	3.8	0.5	4.1	0.6	3.9	0.2	0.777	**0.476**	0.538
Mean VO2 [ml/(h animal)]	92.6	8.6	94	6	97.9	5.1	98.7	5.5	0.184	**0.382**	0.631
Minimum VO2 [ml/(h animal)]	66.6	5.2	67.1	6.9	69.2	4.5	71.4	1.6	0.004	**0.125**	0.983
Maximum VO2 [ml/(h animal)]	118.2	8.4	120.1	11.9	125.8	6.7	124.1	8.7	0.114	**0.699**	0.352
Mean RER [VCO2/VO2]	0.99	0.03	0.98	0.04	0.99	0.04	0.99	0.04	0.874	**0.779**	0.716

SUPPLEMENTAL METHODS

Correlation Analysis

To calculate the significance of gene-gene correlations a randomly permuted dataset was used to generate a null-distribution of correlation values. This null-distribution was used to calculate P values for each gene-gene correlation value. P values were adjusted for multiple testing using the Benjamini-Hochberg approach (Benjamini and Hochberg, 1995).

Overlap between DiffCoEx modules and genes differentially expressed in response to training

In order to discern the overlap between genes differentially expressed (**File S4**) and genes differentially correlated between pre and post-exercise individuals we tested each network module for enrichment of genes whose transcription is exercise dependant using Gene Set Enrichment Analysis. Genes within three modules were found to significantly overlap with genes differentially expressed after training. Modules M3, M9 and M10 were enriched with genes down-regulated after training (FDR < 10%).

Polysome Purification and Polysomal mRNA analysis

Muscle tissue (~0.18g) was powdered in a mortar and pestle with liquid nitrogen, and resuspended in 1ml resuspension buffer (10 mM Tris (pH 8.0), 250 mM KCl, 10 mM MgCl2, 0.5% Triton X-100, 2 mM dithiothreitol, 100 µg/ml cycloheximide, 100 units/ml SUPERase In™ RNase inhibitor (Ambion), and Protease Inhibitor Cocktail (Sigma)). The mixture was then homogenized using PowerLyzer® 24 Bench Top Bead-Based Homogenizer (MO-Bio). Homogenates were incubated on ice for 5 min, and then 150 µl of Tween-deoxycholate mix (1.34 ml of Tween 20, 0.66g of deoxycholate, 18ml of sterile water) was added per 1ml of resuspension buffer, and the samples were briefly vortexed. Samples were incubated on ice for 15 min and then centrifuged at 10,000 g for 10 min at 4°C. The resulting supernatant was layered on a 15–50% linear sucrose gradient (20 mM Tris (pH 8.0), 250 mM KCl, and 10 mM MgCl2) and centrifuged in a RPS40T rotor at 37,000 rpm for 170 min at 4°C. Following centrifugation, the sucrose gradient was fractionated and UV absorption at 260 nm was recorded using BioLogic LP (Bio-Rad). RNA was precipitated by adding 2 volumes of 100% ethanol, re-dissolved in RNase-free water, and treated with phenol-chloroform. The RNA was precipitated by addition of 10% 3 M sodium acetate (pH 5.2) and 2.5 volumes of 100% ethanol and re-dissolved in RNase-free water. Sucrose fractions containing ≥2 polysomes were pooled for subsequent analysis. Total RNA was extracted from muscle using the methods of Caddick et al. (Caddick et al., 2006). The mRNA was then processed for microarray analysis as described above. Statistically significant genes were selected using an absolute $\log_2$ fold change threshold greater than 1 and $p<0.05$.

Mitochondrial Proteomics

Mitochondria were isolated from fresh gastrocnemius muscle of WT and *eif6*$^{+/-}$ mice as previously described (Wittig et al., 2007).

Sample digestion: 50µl of 25mM ammonium bicarbonate (ambic) was added to each mitochondrial pellet. 2µl of a 1%(w/v) Rapigest solution in 25mM ambic was added giving a final concentration of ~0.04%(w/v). For digestion, a volume of sample equivalent to 50µg of protein was made up to 80µl with 25mM ambic and 5µl of 1%(w/v) Rapigest added and the samples heated at 80°C for 10min. Samples were reduced by the addition of 5µl of DTT (9.2mg/mL in 25mM ambic) and heated at 60°C for 10min. Samples were cooled and 5µl of iodoacetamide (33mg/ml in 25mM ambic) was added and samples incubated at room temp for 30min in the dark. Trypsin (Sigma: Porcine trypsin sequencing grade) (1µg) was added and the sample was incubated at 37°C overnight.

The digests were acidified by the addition of 1µl of TFA and incubated at 37°C for 45min. Samples were then centrifuged at 17,000 x g for 30min and supernatants transferred to 0.5mL low-bind tubes. Samples were centrifuged for a further 30min and 10µl transferred to total recovery vials for LC-MS analysis. Pre and post-acidification digest (10µl) were analysed by SDS-PAGE to check for the absence of protein and hence complete digestion.

LC Separation- All peptide separations were carried out using an Ultimate 3000 nano system (Dionex/Thermo Fisher Scientific). For each analysis the sample was loaded onto a trap column (Acclaim PepMap 100, 2cm x 75mm inner diameter, C_{18}, 3mm, 100Å) at 5µl/min with an aqueous solution containing 0.1%(v/v) TFA and 2%(v/v) acetonitrile. After 3min, the trap column was set on-line with an analytical column (Easy-Spray PepMap® RSLC 50cm x 75mm inner diameter, C_{18}, 2mm, 100Å) (Dionex). Peptide elution was performed by applying a mixture of solvents A and B. Solvent A was HPLC grade water with 0.1%(v/v) formic acid, and solvent B was HPLC grade acetonitrile 80%(v/v) with 0.1%(v/v) formic. Separations were performed by applying a linear

gradient of 3.8% to 50% solvent B over 95 min at 300nL/min followed by a washing step (5min at 99% solvent B) and an equilibration step (15 min at 3.8% solvent B). 2µl of each sample was injected.
Analyses on a Quadrupole-Orbitrap instrument- The Q Exactive instrument was operated in data dependent positive (ESI+) mode to automatically switch between full scan MS and MS/MS acquisition. Survey full scan MS spectra (*m/z* 300-2000) were acquired in the Orbitrap with 70,000 resolution (*m/*z 200) after accumulation of ions to $1x10^6$ target value based on predictive automatic gain control (AGC) values from the previous full scan. Dynamic exclusion was set to 20s. The 10 most intense multiply charged ions ($z \geq 2$) were sequentially isolated and fragmented in the octopole collision cell by higher energy collisional dissociation (HCD) with a fixed injection time of 100ms and 35,000 resolution. Typical mass spectrometric conditions were as follows: spray voltage, 1.9kV, no sheath or auxiliary gas flow; heated capillary temperature, 275°C; normalised HCD collision energy 30%. The MS/MS ion selection threshold was set to 1×10^4 counts. A 2Da isolation width was set.

Database search and Protein identification- Raw data files were uploaded into Proteome Discoverer 1.3 and searched against the mouse UniProt database using the Mascot search engine (version 2.4.1). A precursor ion tolerance of 10ppm and a fragment ion tolerance of 0.01Da were used with carbamidomethyl cysteine set as a fixed modification and oxidation of methionine as a variable modification. The false discovery rate (FDR) against a decoy database was 1-5%. The data set was analysed using Progenesis 4.1 LCMS label-free quantification software. The raw profile data was uploaded into the software selecting ESI-FTICR. The top 5 MS/MS spectra were exported from the Progenesis software as a Mascot generic file (mgf) and used for peptide identification using the above settings.

Acetylome Analysis
Total protein from gastrocnemius muscle was extracted from muscle tissue from 3 WT and 3 *eif6*$^{+/-}$ mice according to (Lambertucci et al., 2012). Briefly, muscle tissue was homogenized in extraction buffer (100 mM Tris, pH 7.5; 10 mM EDTA; 100 mM NaF; 10 mM sodium pyrophosphate; 10 mM sodium orthovanadate; 2 mM phenylmethanesulfonyl fluoride; and 0.01 mg/mL aprotinin) at 4°C for 30 sec. After homogenization, Triton X-100 was added to a final concentration of 1%, the samples were incubated for 30 min at 4°C and were centrifuged at 13,000× *g* for 20 min at 4°C. The total protein content was determined using bovine serum albumin as the standard.
Anti-acetyl lysine immunoprecipitations- Anti-acetyl lysine antibody (Abcam) or rabbit IgG control antibody (150mg) were dialysed overnight into coupling buffer (100mM $NaHCO_3$, 500mM NaCl, pH8.5) before conjugating to cyanogen bromide-activated Protein G sepharose (Sigma-Aldrich) according to manufacturer's instructions. 5mg of protein lysate from each sample was made up to 400µl in lysis buffer and the samples pre-cleared using 50µl washed Protein G-Sepharose (Sigma-Aldrich). After rotation for 1 hour at 4°C, samples were centrifuged for 5 minutes at 4000rpm and the supernatant transferred to sterile eppendorfs. Pre-cleared protein lysates were rotated overnight at 4°C with Protein G conjugated anti-acetyl lysine antibody. Samples were centrifuged for 10 minutes at 4000rpm 4°C and the unbound fraction (supernatant) frozen at -20˚C. The pellet was washed in 200µl PBS three times, resuspended in 30µl elution buffer (8M urea, 20mM Tris pH 7.5, 100mM NaCl) and incubated for 30 minutes at room temperature with rotation. The samples were then gently centrifuged and the supernatant collected. The elution step was repeated three times and on the third and final incubation the samples were incubated at 55˚C with gentle agitation for 30 minutes before pooling all elution fractions and storing at -20˚C.
Tandem Mass Tagging- Anti-acetyl lysine immunoprecipitated proteins from wt or eIF6 knock down mice were trypsin digested and then labelled using TMT sixplex Isobaric Mass Tagging Kit (Thermo Scientific) according to manufacturer's instructions. Briefly, proteins were precipitated by the addition of 90µl 100mM triethyl ammonium bicarbonate (TEAB) dissolution buffer, the volume adjusted to 200µl with ultrapure water, 10µl 200mM tris(2-carboxyethyl)phosphine (TCEP) reducing reagent added and the mixture incubated at 55˚C for 1 hour. 10µl 375mM iodoacetamide (in TEAB) was added to each sample and incubated for 30 minutes in the dark. 1320µl of acetone pre-chilled to -20˚C was added to each sample and proteins precipitated overnight at -20˚C. Samples were centrifuged at 8000g and the pellets of precipitated protein air dried before resuspending in 100µl 100mM TEAB and 2.5µg trypsin protease. Proteins were digested overnight at 37°C. 41µl of treatment-specific TMT label reagents were incubated with samples for 2 hours at room temperature. The following labels were used for each treatment arm: WT#3-#4-#5 were labelled with 126, 127, 128 and *eif6*$^{+/-}$ #4-#5-#6 were labelled with 129, 130 and 131. The labeling reaction was quenched by incubating the samples with 8µl 5% hydroxylamine for 15 minutes.

Mass spectrometry- All 6 samples were pooled, dried down to 500µl using a Savant SPD111V SpeedVac Concentrator (Thermo Scientific) at 45°C and made up to 1ml with mobile phase A solution (10mM KH_2PO_4, pH3 with phosphoric acid then add 20% CH_3CN). Samples were fractionated by strong cation exchange high

performance liquid chromatography (SXC-HPLC) using an Ettan LC HPLC machine (Amersham Pharmacia Biotech) and a 100x2.1mm polysulfoethyl aspartamide SCX column with UV detection at 214nm. Elution at a flow of 1 ml/min was performed with a linear gradient between mobile phase A and mobile phase B (10mM KH_2PO_4, 500mM KCl, pH3 with phosphoric acid then add 20% CH_3CN) over a 90 minute run. 16 fractions were collected, dried completely using a SpeedVac (as above) before resuspending in 0.1% trifluoroacetic acid and desalted using C_{18} ZipTips according to manufacturer's instructions (Millipore). Desalted samples were dried completely, resuspended in 10µl 0.1% formic acid and analysed using an Orbitrap Velos ETD mass spectrometer (Thermo Scientific) and Thermo Proteome Discoverer 1.3 software. Each fraction was run in triplicate and peptides identified using Swissprot. The raw data files were processed and quantified using Proteome Discoverer software v1. (Thermo Scientific) and searched against the UniProt/SwissProt Human database release version 57.3 (20326 entries) using the SEQUEST HT algorithm. Peptide precursor mass tolerance was set at 10 ppm, and MS/MS tolerance was set at 0.6 Da. Search criteria included the TMT N-Term and lysine as static modifications, carbamidomethylation of cysteine, oxidation of methionine and deamidation of Asn and Gln and lysine as fixed modifications. Searches were performed with full tryptic digestion and a maximum of 3 missed cleavage sites was allowed. The reverse database search option was enabled and all peptide data was filtered to satisfy false discovery rate (FDR) of 5%. The Proteome Discoverer software generates a reverse "decoy" database from the same protein database and any peptides passing the initial filtering parameters that were derived from this decoy database are defined as false positive identifications. The minimum cross-correlation factor (Xcorr) filter was readjusted for each individual charge state separately to optimally meet the predetermined target FDR of 5% based on the number of random false positive matches from the reverse decoy database. Thus each data set has its own passing parameters. Quantitation was performed using a peak integration window tolerance of 0.05 Da with the integration method set as the most confident centroid. Protein ratios represent the median of the raw measured peptide ratios for each protein. Each protein included in our study was identified from at least 2 peptides with high/medium confidence. Proteins recorded as uncharacterized by the software were returned with a gene ID.

ROS imaging

A Nikon E-Ti inverted microscope with a motorised stage (TI-S-EJOY, Nikon) for a 35mm petri dish was used. The C1 confocal (Nikon Instruments Europe BV, Surrey, UK) comprised a diode (UV) 405nm and argon laser with 488nm excitation. Acquisition software was EZC1 V.3.9 (12 bit). MitoSox Red was excited sequentially at 405nm using a diode laser and 488nm using an argon laser, each passing through a main dichroic and secondary beam splitter with the emission collected through a 605/15 filter to a detector. DAF-FM was excited at 488nm and emission recorded between 515/30nm, bright field images were acquired using the 488nm laser to a CCD. The objective was a PlanApo VC x60A/1.2NA/0.27 mm working distance water immersion. Pinhole size was 150 µm with a 1.68 µsec pixel dwell time in all cases. Regions of interest for determination of fluorescence/area were selected and quantified as previously described (Pearson et al., 2014). All experiments were performed at approximately 28°C. Images were captured every 5 minutes of the 30 minute protocol.

FDB fibre contraction was induced using platinum electrodes using field stimulations with a stimulation at 5-10 and 20-25 minutes over a 30 minute protocol. The stimulation comprised a bipolar pulse of 2ms duration for 0.5 sec repeated every 5 secs at 50Hz. Fibres were loaded by incubation in 2ml D-PBS containing 250 nM MitoSox Red or 10µM DAF-FM DA for 30 minutes at 37 °C in a tissue culture incubator. Cells were then washed and maintained in 2ml MEM-solution during the experimental protocol. DAF-FM DA readily diffuses into cells and within the cytoplasm releases DAF-FM by the action of intracellular esterases. DAF-FM is essentially non fluorescent until it is nitrosylated by products of oxidation of NO, resulting in DAF-FM triazole that exhibits about a 160-fold greater fluorescence efficiency (Kojima et al., 1999).

References

Benjamini, Y., Hochberg, Y., 1995. Controlling the false discovery rate: a practical and powerful approach to multiple testing. J. R. Stat. Soc. Ser. B Methodol. 57, 289–300. doi:10.2307/2346101

Caddick, M.X., Jones, M.G., Van Tonder, J.M., Le Cordier, H., Narendja, F., Strauss, J., Morozov, I.Y., 2006. Opposing signals differentially regulate transcript stability in Aspergillus nidulans. Mol. Microbiol. 62, 509–519. doi:10.1111/j.1365-2958.2006.05383.x

Kojima, H., Urano, Y., Kikuchi, K., Higuchi, T., Hirata, Y., Nagano, T., 1999. Fluorescent indicators for imaging nitric oxide production. Angew. Chemie - Int. Ed. 38, 3209–3212. doi:10.1002/(SICI)1521-3773(19991102)38:21<3209::AID-ANIE3209>3.0.CO;2-6

Lambertucci, A.C., Lambertucci, R.H., Hirabara, S.M., Curi, R., Moriscot, A.S., Alba-Loureiro, T.C., Guimarães-Ferreira, L., Levada-Pires, A.C., Vasconcelos, D.A.A., Sellitti, D.F., Pithon-Curi, T.C., 2012. Glutamine Supplementation Stimulates Protein-Synthetic and Inhibits Protein-Degradative Signaling Pathways in Skeletal Muscle of Diabetic Rats. PLoS One 7. doi:10.1371/journal.pone.0050390

Pearson, T., Kabayo, T., Ng, R., Chamberlain, J., McArdle, A., Jackson, M.J., 2014. Skeletal muscle contractions induce acute changes in cytosolic superoxide, but slower responses in mitochondrial superoxide and cellular hydrogen peroxide. PLoS One 9. doi:10.1371/journal.pone.0096378

Wittig, I., Carrozzo, R., Santorelli, F.M., Schägger, H., 2007. Functional assays in high-resolution clear native gels to quantify mitochondrial complexes in human biopsies and cell lines. Electrophoresis 28, 3811–20. doi:10.1002/elps.200700367
