# Supplementary material for: The Role of Eif6 in Skeletal Muscle Homeostasis Revealed by Endurance Training Co-expression Networks
Source: Cell Rep. 2017 Nov 7;21(6):1507–20. doi: 10.1016/j.celrep.2017.10.040 (PMC5695912; doi:10.1016/j.celrep.2017.10.040)
Supplement: Document S2. Article plus Supplemental Information [file mmc4.pdf]

Cell Reports

Article

The Role of *Eif6* in Skeletal Muscle Homeostasis Revealed by Endurance Training Co-expression Networks

Graphical Abstract

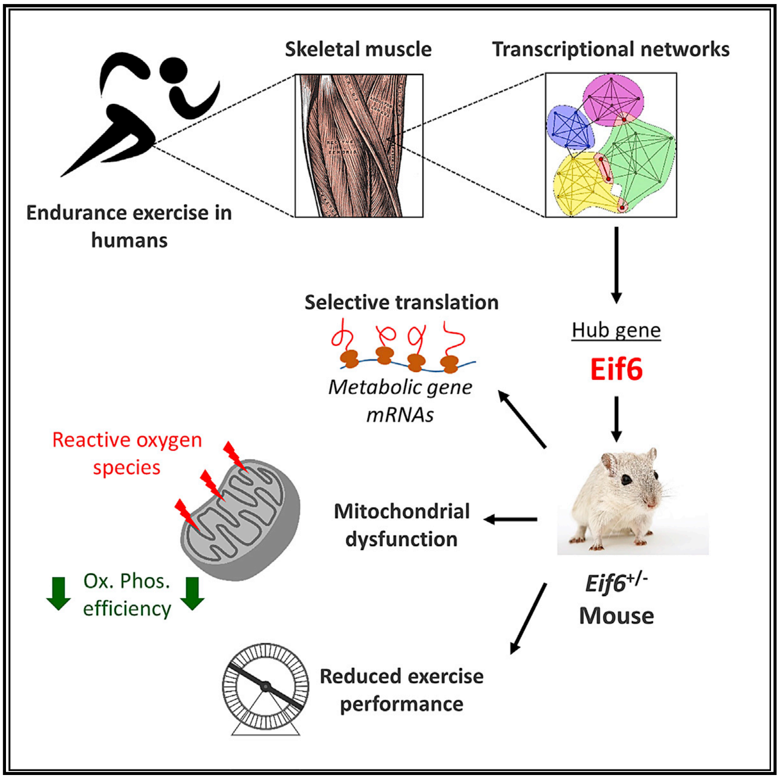

Authors

Kim Clarke, Sara Ricciardi, Tim Pearson, ..., Claude Bouchard, Stefano Biffo, Francesco Falciani

Correspondence

f.falciani@liverpool.ac.uk

In Brief

Clarke et al. use data-driven reverse engineering to uncover the role of *Eif6* in controlling skeletal muscle homeostasis. They achieve this by analyzing the complex network of genes that controls skeletal muscle adaptation to endurance exercise, together with *in vivo* studies of *eif6*$^{+/-}$ mice that show decreased respiration efficiency, increased ROS production, and reduced exercise performance.

Highlights

- Endurance exercise profoundly affects the structure of gene networks
- *Eif6* is a hub in gene networks responsible for muscle metabolism and protein synthesis
- Mitochondrial metabolic capacity altered in muscle from *Eif6*$^{+/-}$ mice
- Eif6 haploinsufficiency increased ROS generation and reduced exercise performance

Data and Software Availability

GSE47874

Clarke et al., 2017, Cell Reports *21*, 1507–1520
November 7, 2017 © 2017 The Authors.
https://doi.org/10.1016/j.celrep.2017.10.040

CellPress

Cell Reports
Article

OPEN ACCESS
CellPress

The Role of *Eif6* in Skeletal Muscle Homeostasis Revealed by Endurance Training Co-expression Networks

Kim Clarke,[1] Sara Ricciardi,[2] Tim Pearson,[3] Izwan Bharudin,[1,4] Peter K. Davidsen,[1] Michela Bonomo,[1] Daniela Brina,[2] Alessandra Scagliola,[2] Deborah M. Simpson,[5] Robert J. Beynon,[5] Farhat Khanim,[6] John Ankers,[1] Mark A. Sarzynski,[7] Sujoy Ghosh,[7] Addolorata Pisconti,[1] Jan Rozman,[8] Martin Hrabe de Angelis,[8] Chris Bunce,[6] Claire Stewart,[9] Stuart Egginton,[10] Mark Caddick,[1] Malcolm Jackson,[11] Claude Bouchard,[7] Stefano Biffo,[2,12] and Francesco Falciani[1,13,*]
[1]Institute of Integrative Biology, University of Liverpool, Liverpool L69 7ZB, UK
[2]Molecular Histology and Cell Growth Unit, INGM - Fondazione Istituto Nazionale Genetica Molecolare, 20122 Milan, Italy
[3]Department of Medicine, University of East Anglia, Norwich Research Park, Norwich NR4 7TJ, UK
[4]School of Biosciences and Biotechnology, Universiti Kebangsaan Malaysia, 43600 Bangi, Selangor, Malaysia
[5]Centre for Proteome Research, Institute of Integrative Biology, University of Liverpool, Liverpool L69 7ZB, UK
[6]School of Biosciences, University of Birmingham, Birmingham B15 2TT, UK
[7]Pennington Biomedical Research Center, Baton Rouge, LA 70808, USA
[8]German Mouse Clinic, Institute of Experimental Genetics, Helmholtz Zentrum München, German Research Center for Environmental Health, Ingolstädter Landstr. 1, 85764 Neuherberg, Germany
[9]Sport and Exercise Sciences, Liverpool John Moores University, Liverpool L3 3AF, UK
[10]School of Biomedical Sciences, University of Leeds, Leeds LS2 9JT, UK
[11]Institute of Ageing and Chronic Disease, University of Liverpool, Liverpool L7 8TX, UK
[12]Dipartimento di Bioscienze, Università degli Studi di Milano, 20133 Milan, Italy
[13]Lead Contact
*Correspondence: f.falciani@liverpool.ac.uk
https://doi.org/10.1016/j.celrep.2017.10.040

SUMMARY

Regular endurance training improves muscle oxidative capacity and reduces the risk of age-related disorders. Understanding the molecular networks underlying this phenomenon is crucial. Here, by exploiting the power of computational modeling, we show that endurance training induces profound changes in gene regulatory networks linking signaling and selective control of translation to energy metabolism and tissue remodeling. We discovered that knockdown of the mTOR-independent factor *Eif6*, which we predicted to be a key regulator of this process, affects mitochondrial respiration efficiency, ROS production, and exercise performance. Our work demonstrates the validity of a data-driven approach to understanding muscle homeostasis.

INTRODUCTION

Physical activity or regular exercise is essential for homeostasis of the musculoskeletal system. The benefits of regular exercise are such that physically active individuals have significantly lower mortality rates, regardless of age and lifestyle (Kokkinos, 2012). Exercise is recognized as an effective strategy for the management of chronic conditions such as obesity (Blair and Brodney, 1999), type 2 diabetes (Colberg et al., 2010), and chronic obstructive pulmonary disease (COPD) (Turan et al., 2011).

In the last decade, transcriptomics studies have succeeded in revealing genome-wide transcriptional signatures linked to the endurance training response (Phillips et al., 2013; Timmons et al., 2010; Teran-Garcia et al., 2005; Schmutz et al., 2006). However, most of these studies are also limited to a description of the differentially expressed genes and the functions that they represent. Perhaps the most advanced approach in a recent study is demonstrated by Phillips et al. (2013), who use a correlation-based approach to identify genes linked to gains in muscle mass or age. By utilizing pathway databases representing knowledge-driven gene-to-gene interactions, they identify molecular networks underlying both aging and muscle hypertrophy. While their study highlighted that training induces a molecular reprogramming of the muscle, including showing that the differential expression of mTOR-related genes is linked to muscle hypertrophy, it still relies on existing knowledge for pathway identification.

We reasoned that a data-driven approach may successfully identify truly novel regulatory networks. Encouraged by previous approaches (Turan et al., 2011; Davidsen et al., 2014), we selected one of the most comprehensive endurance training studies in humans, the HERITAGE Family Study (Bouchard et al., 1995), and applied a reverse-engineering strategy to develop a genome-wide network model of muscle homeostasis.

The analysis of the model revealed that co-expression of genes found within core metabolic and translational pathways, such as oxidative phosphorylation and ribosome assembly, is a predominant feature of these networks and that the eukaryotic translation initiation factor *Eif6* (Ceci et al., 2003; Sanvito et al., 1999) is the most connected ribosome associated factor in the network. Analysis of the *Eif6*$^{+/-}$ haploinsufficient mouse

This is an open access article under the CC BY license (http://creativecommons.org/licenses/by/4.0/).

recapitulated the transcriptional signatures predicted by the HERITAGE study. Furthermore, an assessment of mitochondrial functionality and muscle performance in *Eif6* heterozygote mice revealed alterations in electron transport chain dynamics, superoxide generation, and reduced exercise capacity, further supporting the role of this factor in regulating muscle energy homeostasis.

RESULTS

Endurance Training Affects Gene Regulatory Networks in Human Skeletal Muscle

We first identified gene networks whose structure is different between pre- and post-training skeletal muscles. We did this by applying the bioinformatics method DiffCoEx (Tesson et al., 2010) to the HERITAGE study, which includes transcriptome and physiological measurements (Table S1) for 41 human participants at baseline and after 20 weeks of supervised endurance training.

The inferred network (Figure 1) confirmed the hypothesis that endurance training promotes a large-scale rewiring of transcriptional networks. First, we identified differential co-expression networks involving 8,893 unique genes, which the algorithm organized into 25 interconnected gene clusters (network modules) (Figure S1). Of these 8,893 genes, 981 (11%) were detected as differentially expressed in response to training, and only 3 of the 25 modules were enriched with genes responding to training (Figure S1). Network rewiring via differential co-expression therefore involves a larger set of genes and functions than identified by changes in mRNA expression levels alone. Larger modules (more than 10 genes in size) were tested for functional enrichment. Indeed, their functional profile was consistent with skeletal muscle homeostasis and included biological processes related to energy metabolism, muscle fiber contractile elements, and tissue remodeling (Figure 1).

We found that the network reflects several aspects of skeletal muscle homeostasis, including neuromuscular junction signaling, modulation of regulatory factors, and, in turn, the effector functions responsible for the endurance training response. Module 1 (M1) was enriched in cell signaling pathways including genes controlling neuromuscular synaptic transmission. M3, M4, M8, and M10 represented a set of modules characterized by regulatory factors controlling processes such as chromatin modification, transcription, mRNA processing, mRNA transport, and regulation of translation. Finally, M2 and M5–M9 represented three different types of effector functions, namely energy metabolism, tissue remodeling, and muscle fiber structural components.

The linkage between signaling and effector functions was consistent with existing literature. However, we also observed that the correlation patterns between genes in energy metabolism (oxidative phosphorylation, the tricarboxylic acid [TCA] cycle, and glycolysis) and translation were different in pre- and post-training individuals (Figure 1). Interestingly, most of the enriched translation related genes (in M8 and M2) were not ribosomal structural components. Instead, we could identify regulatory factors such as the eukaryotic initiation factors (eIFs) (14/59 translation-related genes) (Figure 1), which are known to control translation of specific proteins in the rate-limiting phase of initiation and have previously been shown to participate in signaling events such as hypoxia (*Eif2a*) (Liu et al., 2006), mTOR-dependent regulation of energy metabolism (*Eif4e*) (Morita et al., 2013), neoplastic transformation (*Eif6*) (Sanvito et al., 1999; Gandin et al., 2008), and lipid metabolism (*Eif6*) (Brina et al., 2015).

Analysis of the Ribosome Assembly Sub-network Identifies *Eif6* as a Potential Regulator of Energy Metabolism in Skeletal Muscles

The co-localization of eIF genes and genes related to energy metabolism and other important functions involved in muscle homeostasis suggests that eIF proteins may have a broad role in the response to endurance training. To further investigate this hypothesis, we analyzed the neighborhood of eIFs in the differential co-expression network. We focused on visualizing the linkage between relevant canonical pathways (Kyoto Encyclopedia of Genes and Genomes [KEGG] pathways) and a set of 20 relevant physiological measurements (Table S1) included in the HERITAGE study (Figure 2A).

Consistent with the previous observation, we found that oxidative phosphorylation, ribosome, spliceosome, and neuroactive ligand receptor interaction were the most connected KEGG pathways among the eIF sub-network (Figure 2B). The network also represented several highly connected physiological measurements (leptin, phosphofructokinase). Interestingly, *Eif6* was the network node with the highest degree (Figure 2B), which prompted us to explore its neighborhood in detail (Figure 2C). We found that its most significant links were with genes involved in oxidative phosphorylation (false discovery rate [FDR] $< 10^{-6}$ for pathway enrichment), followed by an index of systemic inflammation (C-reactive protein, FDR < 0.001) and, as expected, the ribosome KEGG pathway (FDR < 0.001).

Inspection of separate baseline and post-training correlation networks built around *Eif6* revealed an increased connectivity (degree of 4 increased to degree of 11) between *Eif6* and KEGG pathways following endurance training (Figures 2D and 2E).

The Haploinsufficient *Eif6*$^{+/-}$ Mouse Mimics the Predicted *Eif6* Transcriptional Signature

The correlation analysis described above predicts that changes in the activity of *Eif6* may be causally linked to changes in the expression of genes involved in several important pathways, among which oxidative phosphorylation was the strongest candidate (Figure 2C).

We then decided to test this hypothesis by assessing whether modulation of *Eif6* in skeletal muscle of mice would result in the same *Eif6* transcriptional signature that we predicted from the HERITAGE dataset. While human *vastus lateralis* muscle is considered a mixed fiber type (Staron et al., 2000), it is also highly variable (Simoneau and Bouchard, 1989), and different fiber types induce different transcriptional programs (Wang et al., 2004). To account for this and to assess the effect of *Eif6* modulation in conditions of different metabolic pathway activity we analyzed three different skeletal muscles (*soleus*, *gastrocnemius*, and *tibialis anterior*) in wild-type (WT) and mutant *Eif6*$^{+/-}$ mice. These represent a preference for slow-twitch oxidative,

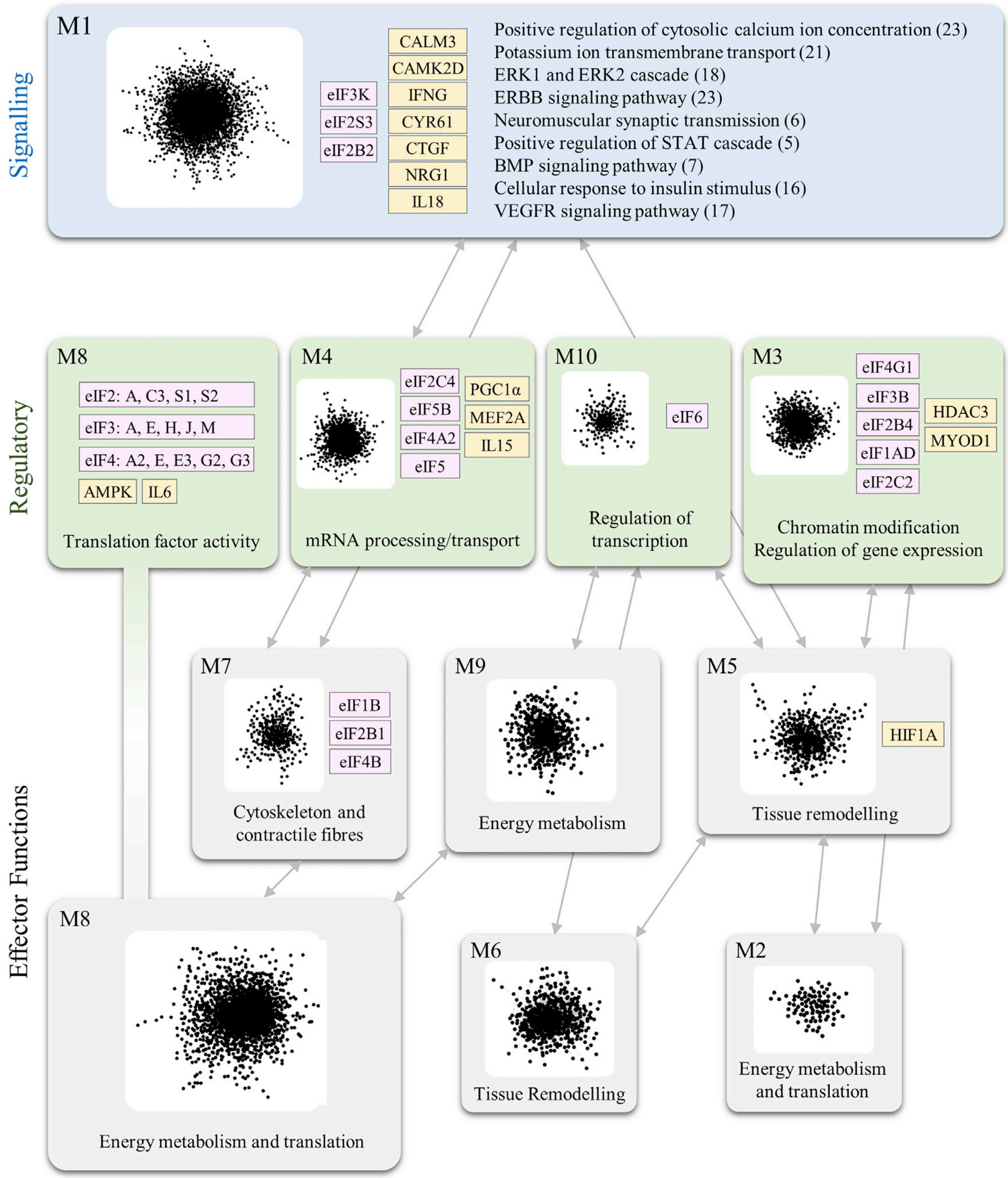

Figure 1. Effects of Endurance Training on Gene Co-expression in Skeletal Muscle

This network represents the 10 largest gene modules identified by the gene co-expression analysis (module size is proportional to the number of genes). Genes within each module showed highly significant alteration in gene-gene pairwise correlation between trained and untrained states (FDR < 1%). Modules connected by arrows showed significant inter-module alterations in correlation between trained and untrained states (FDR < 10%). Each module has been annotated with genes known to play a key role in muscle biology (gold) and in the eukaryotic initiation factors (pink) found within that module. M1 is labeled with enriched gene ontology terms reflecting signaling pathways. Every other module has been summarized using a functional term representative of the enriched gene ontology terms.

mixed fiber type and fast-twitch, glycolytic fibers, respectively. The *Eif6*$^{+/-}$ mouse model shows a 50% reduction in eIF6 expression (Gandin et al., 2008), but this does not affect basal translational rates and does not induce any changes in fiber-type composition (Figure S2A; Table S2) or in the number of mitochondria (measured as mtDNA-to-nuclear DNA [nDNA] ratio) (Figure S2B). Therefore, these animals are a good model for studying the specific role of *Eif6* in skeletal muscles.

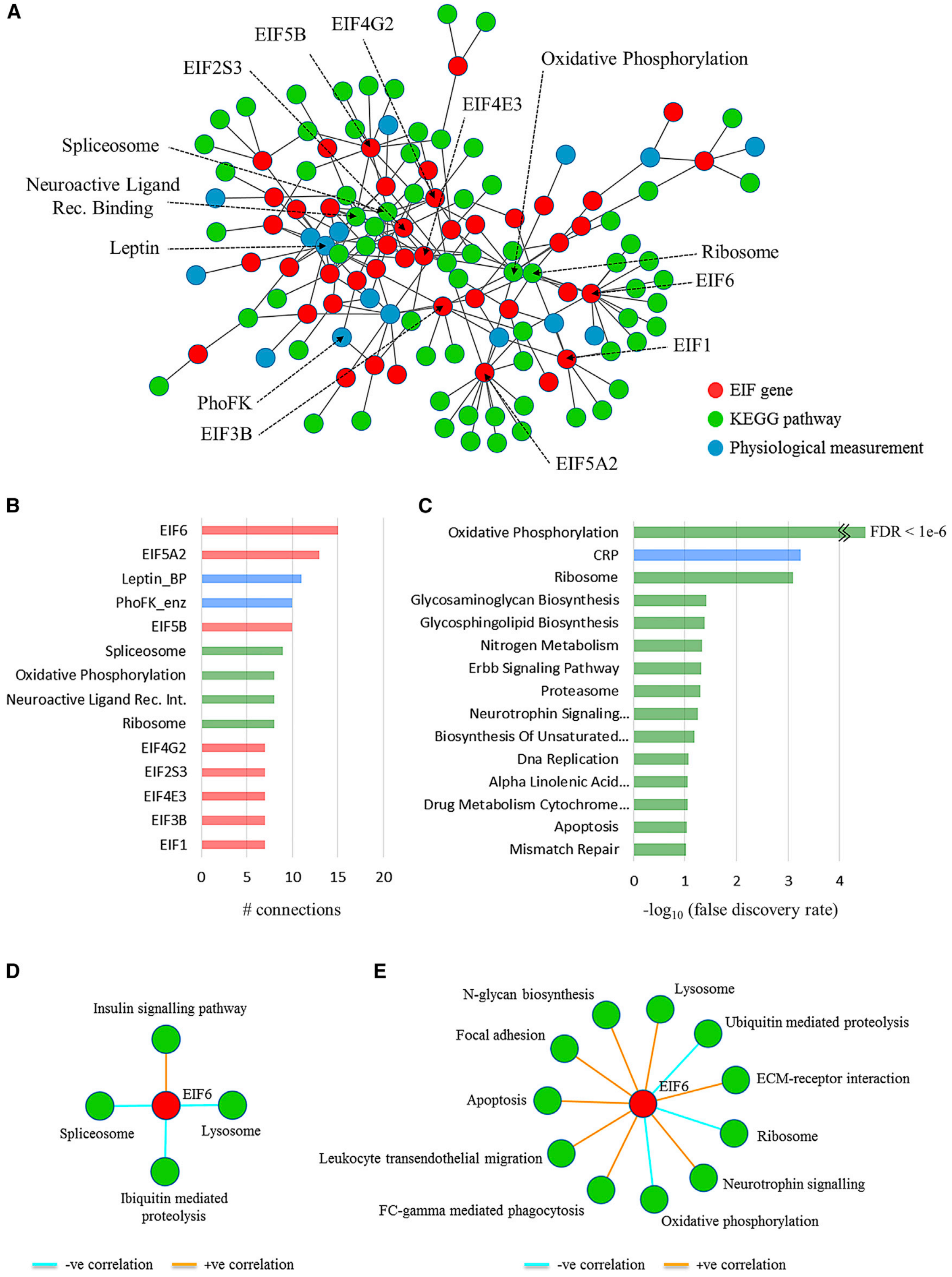

Figure 2. Network-Based Integration of EIF Genes, Physiological Measurements, and Pathway Activity Reveals *Eif6* as the Most Connected Factor

(A) This network represents the integration of multiple analyses. Connections between eIF genes and physiological measurements represent regression analysis using baseline eIF gene expression as a predictor variable (FDR < 10%). Connections between eIF genes and KEGG pathways represent significant enrichment of genes from a KEGG pathway within genes highly differentially co-expressed with the eIF.

(legend continued on next page)

OPEN ACCESS
CellPress

	Gastro	Soleus	Tib. Ant.
Oxidative phosphorylation	+57	+26	-21
Ubiquitin mediated proteolysis	+30	-28	+18
Neurotrophin signaling pathway	-23	-28	-16
Lysosome	-27	-20	
Fc gamma R-mediated phagocytosis	-18		-13
Focal adhesion	-33		
ECM-receptor interaction	-16		
Leukocyte transendothelial migration	-18		
Ribosome	-25	-19	
Insulin signaling pathway	-21	-25	
ErbB signaling pathway	-14	-16	
Endocytosis	-31		+24
Chromatin Modification	+50		+32
Tight junction	-20		
Regulation of actin cytoskeleton	-31		
Inositol phosphate metabolism		+15	
MAPK signaling pathway		-43	
Wnt signaling pathway		-27	
Long-term potentiation		-19	
GnRH signaling pathway		-24	
Adipocytokine signaling pathway		+16	

Up-regulated in *eif6*$^{+/-}$ muscle; Down-regulated in *eif6*$^{+/-}$ muscle — Consistent with human correlation analysis both in terms of presence and direction

Up/Down-regulated in *eif6*$^{+/-}$ muscle — Consistent with human correlation analysis but direction is not consistent

Up/Down-regulated in *eif6*$^{+/-}$ muscle — Non-significant in human correlation analysis

Figure 3. *Eif6* Haploinsufficiency in Mouse Skeletal Muscle Recapitulates the Predictions from the Human Model

The figure shows the KEGG pathways enriched in genes up- or downregulated in the three *Eif6*$^{+/-}$ skeletal muscle types compared to WT of the same muscle type (FDR < 10%). The number and the direction of change (positive, upregulated; negative, downregulated) of the enriched genes are indicated. Pathways colored red and green represent those matching the human *Eif6* correlation analysis (shown in Figure 3D and 3E) of consistency between direction of correlation and change in expression. Pathways highlighted in yellow represent those terms matching the human correlation analysis but without consistency between direction of correlation and change in expression.

Indeed, we discovered that the transcriptional states of WT and *Eif6*$^{+/-}$ mice were different (genes differentially expressed in the *gastrocnemius*: 2,651 upregulated, 829 downregulated; soleus: 2,482 upregulated, 1,846 downregulated; *tibialis anterior* (TA): 1,361 upregulated, 958 downregulated; FDR < 10%) and that the profile of the *gastrocnemius* muscle mirrored quite accurately the predicted *Eif6* signature both in functional profile and in direction of change. More precisely, we found 20 KEGG pathways significantly enriched within the differentially expressed genes from at least 1 muscle type (Figure 3). *Gastrocnemius* muscle had the largest overlap between pathways identified in both mouse and human analysis, with 8/14 (57%) KEGG pathways altered in a direction that was consistent with the correlation analysis using the human data (Figure 3). For these reasons, we decided to focus on *gastrocnemius* muscle in further analysis. Genes both differentially expressed in the *Eif6*$^{+/-}$ *gastrocnemius* muscle and correlated to the expression of *Eif6* in the HERITAGE dataset were enriched with genes related to energy metabolism, including oxidative phosphorylation complexes (e.g., *Atp5l*, *Ndufs4*), mitochondrial function (e.g., *Sod2*, *Gfm2*), and glucose metabolism (e.g., *Pdhb*) (Figure 3). Enriched functions unrelated to energy metabolism included extracellular matrix (ECM)-receptor interaction (e.g., *Thbs3*, *Tnxb*), focal adhesion (e.g., *Itgb1*), ubiquitin-mediated proteolysis (e.g., *Stub1*, *Tceb2*), ribosome, ErbB signaling (e.g., *Akt2*, *Mapk3*), and regulation of actin cytoskeleton (e.g., *Fgfr1*, *Wasf2*) (Figure 3). Interestingly, a group of genes related to chromatin modification were enriched within genes regulated by *Eif6*, particularly histone deacetylases (e.g., *Sirt6*, *Hdac6*) (Table S3), suggesting activity at both the transcriptional and the post-transcriptional levels.

These results further support the hypothesis that *Eif6* is an important regulator of the transcriptional response to endurance exercise and identify a set of core functions whose transcription is affected by *Eif6* haploinsufficiency.

Eif6 Inactivation Induces Complex Alterations in the Mitochondria Proteome

To assess the functional consequences of *Eif6* haploinsufficiency, we used nanoflow liquid chromatography coupled to tandem mass spectrometry to characterize the mitochondrial proteome from *gastrocnemius* muscle of WT and *Eif6*$^{+/-}$ mice (Figure 4A). This revealed 120 differentially expressed proteins (< 10% FDR) (Figure 4B; Table S7) including factors involved in oxidative phosphorylation and in diverse mitochondrial processes (Figures 4C and 4D). Components of the oxidative phosphorylation pathway, mitochondrial ribosome, and mitochondrial

(B) The total number of connections (degree) of the top 14 most highly connected components of the network (degree > 6).
(C) Significance of enrichment of KEGG pathways and phenotypic measurements connected to *Eif6* in the network.
(D and E) Network visualization of KEGG pathways enriched (FDR < 10%) within genes significantly correlated (FDR < 5%) with *Eif6* expression in untrained (n = 2,177 genes) (D) and trained (n = 2,022 genes) (E) individuals separately.

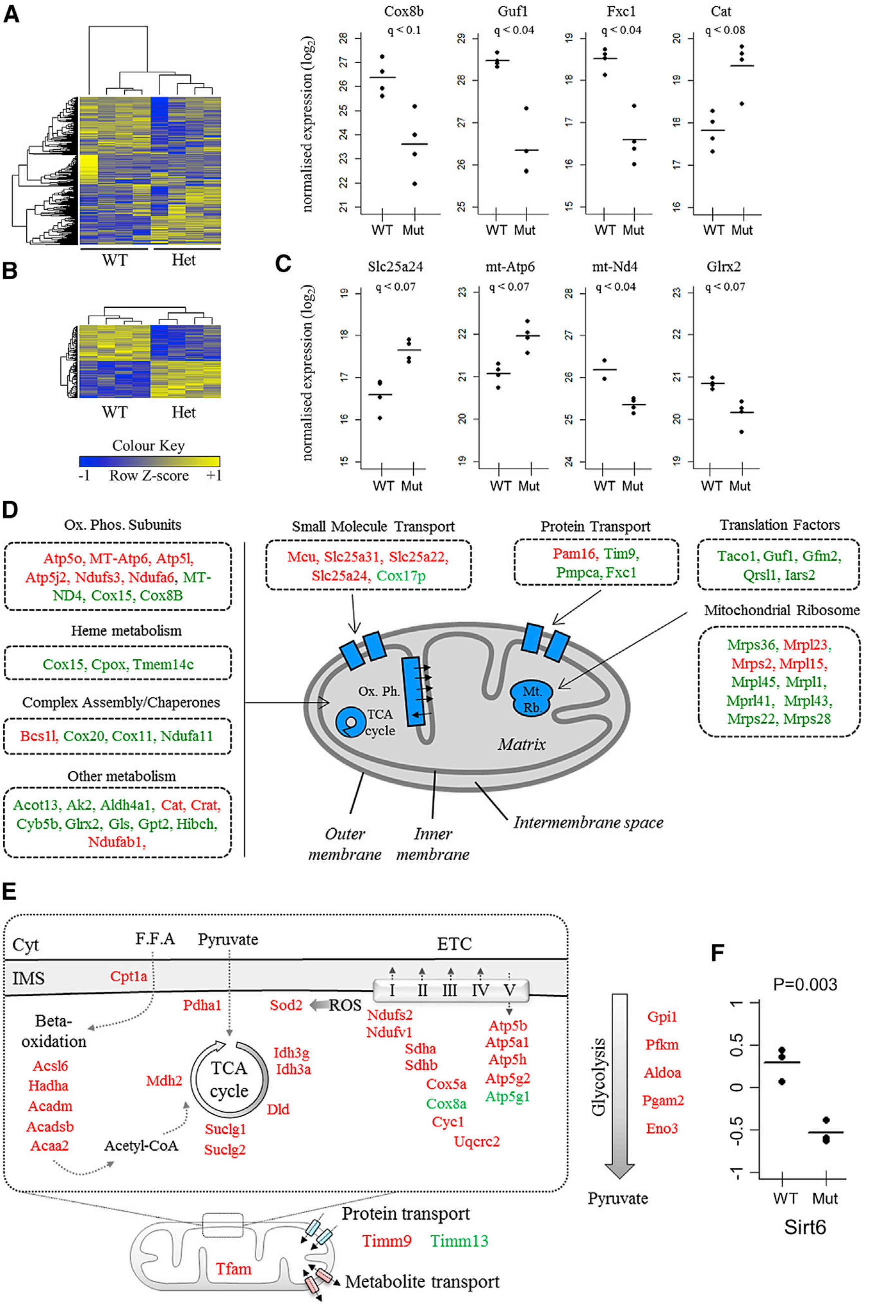

Figure 4. Functional Genomics Profiling of *Eif6*$^{+/-}$ Muscle Reveals Substantial Mitochondrial Reprogramming

(A and B) Representation of the proteomics data using a heatmap. Row data are standardized for visualization purposes. Shown are all proteins for which a measurement was obtained for all 8 samples (918 proteins) (A). Shown are the differentially expressed proteins with a FDR < 10% (120 proteins) (B).

(C) Vertical dot plots representing a selection of the differentially expressed mitochondrial factors revealed by the proteomics analysis (FDR < 10%).

(D) Diagrammatic representation of proteomics data showing differentially expressed mitochondrial proteins (green, downregulated; red, upregulated; FDR < 10%).

(E) Diagrammatic representation of polysomal mRNA analysis of *Eif6*$^{+/-}$ and WT skeletal muscle showing altered mRNA loading of mitochondria-related genes; green, downregulated; red, upregulated; absolute $\log_2$-fold change > 2, $p < 0.05$.

(F) Ratio of polysomal versus total *Sirt6* mRNA in WT and *Eif6*$^{+/-}$ muscle, $p = 0.003$.

inner membrane (such as calcium channels) were both up- and downregulated (Figure 4D). Notable upregulated proteins included small-molecule transporters such as the calcium uniporter MCU, the phosphate carrier SCMC1, the cytoprotective enzyme catalase (CAT), and the translocation factors PAM16 and PAM9. Conversely, downregulated proteins included translocation factors TIM9, TIMM10B, and mitochondrial-processing peptidase subunit alpha; mitochondrial translation factors TACO1, GUF1, RRF2mt, and Glu-AdT subunit A; the oxidoreductase GLRX2; and mitochondrial protein complex assembly chaperones COX20, COX11, and NDUFA11.

In summary, we show that *Eif6* knockdown results in profound changes in mitochondrial protein composition, likely affecting the control of energy metabolism *in vivo*.

Eif6 Regulates the Translation of a Specific Set of Transcripts Encoding for Energy Metabolism-Related Proteins

Having observed changes at both mRNA and protein levels and given the known role of eIF6 in translation initiation, we tested whether *Eif6* haploinsufficiency leads to preferential translation of a subset of mRNAs in mouse *gastrocnemius* muscle using polysomal microarray analysis (Figures 4E and S3). We found that 346 unique mRNAs were significantly enriched in ribosomes from *Eif6*$^{+/-}$ or WT mice ($p < 0.05$ and >2-fold) (Table S8). These were dominated by genes related to metabolism and mitochondrial function such as oxidative phosphorylation, acetyl-coenzyme A (CoA) metabolism, TCA cycle, glycolysis, fatty acid metabolism, and pyruvate metabolism (Figure 4E). Further analysis revealed that a key epigenetic regulatory factor linked to metabolic homeostasis, *Sirt6* (Zhong et al., 2010), that was also correlated to and regulated by *Eif6* in human and mouse muscle, respectively, was differentially translated (Figure 4F).

Highly translated mRNAs may be among the most abundant proteins in the cell. Consequently, we may expect that, despite the influence of mitochondrial protein transport and degradation, differences in the mitochondrial proteome may at least partially

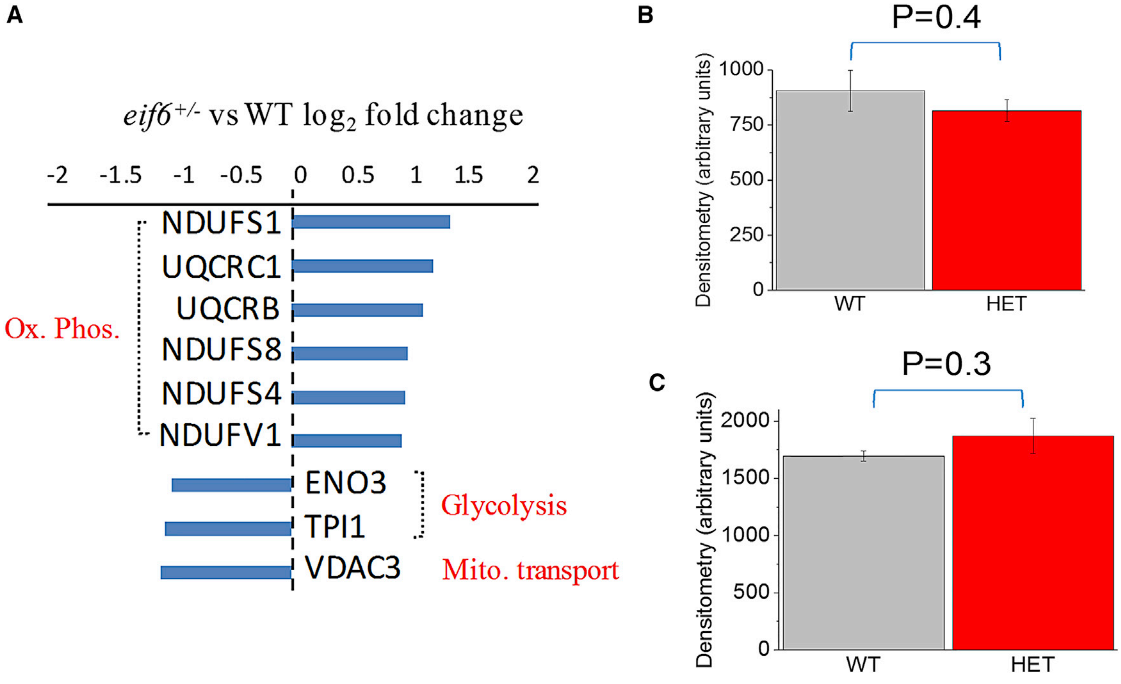

Figure 5. *Eif6* Haploinsufficiency Induces Alterations in Protein Acetylation

(A) Differential protein acetylation in whole-skeletal-muscle lysates determined by MS ($p < 0.05$, absolute $\log_2$-fold change > 1).

(B and C) Protein expression of NDUFS4 (B) and ENO3 (C) determined by western blotting in wild-type (WT) and *Eif6*$^{+/-}$ (HET) skeletal muscle. Data are represented as mean ± SEM.

match the results of the polysome profiling analysis. We therefore tested whether differentially expressed proteins in the mitochondria were also differentially translated. We discovered that 41 proteins (~30% of the total number of differentially expressed proteins in the mitochondria) were enriched in the polysomal fraction of highly translated mRNAs (Table S4). This percentage is significantly higher than expected by random chance (FDR < 10^{-3}).

These results reveal that alterations in abundance of transcripts observed in *Eif6*$^{+/-}$ muscle are accompanied by complex changes at the mRNA loading and protein level and suggest that these could be accompanied by changes in epigenetic status.

Acetylation Status of Metabolic Enzymes Is Altered in *Eif6*$^{+/-}$ Muscle

Several genes involved in the control of protein acetylation were differentially regulated at the mRNA level (including *Sirt1*, *Sirt6*, *Hdac1*, *Hdac6*, *Hdac7*, and *Hdac9*) and differentially translated (including *Sirt4*, *Sirt6*) (Table S3). Since changes in protein acetylation is a well-known mechanism to regulate enzymatic activity (Zhao et al., 2010), it is possible that changes in protein acetylation may be one of the mechanisms by which *Eif6* controls muscle physiology. Because of the diversity of genes involved in the control of protein acetylation, we couldn't make any specific hypothesis on the proteins that may be affected or the direction of change.

We therefore tested this hypothesis by using an open-ended approach based on mass spectrometry of muscle tissue protein extracts immunoprecipitated using an anti-lysine antibody. The mass spectrometry analysis on whole-*gastrocnemius*-muscle lysates revealed 19 differentially acetylated proteins ($p < 0.05$) (Table S5) including a coordinate hyper-acetylation of oxidative phosphorylation components, including four complex I and two complex III subunits, and reduced acetylation of two subunits of glycolytic enzymes ENO3 and TPI1 and the mitochondrial metabolite transporter VDAC3 (Figure 5A). Since acetylation can change activity without changing protein levels, we performed a western blot analysis of two of the differentially acetylated proteins (ENO3 and NDUSF4) and showed that no changes in protein levels were detected (Figures 5B, 5C, and S4).

Skeletal Muscle of *Eif6*$^{+/-}$ Mice Is Less Efficient in Utilizing Oxygen for Respiration and Develops High Levels of Reactive Oxygen Species

Our functional genomics analyses suggested a significant alteration in mitochondrial energy metabolism. The nature of the downstream effects was investigated further by measuring oxygen utilization by live isolated muscle fibers from WT and *Eif6*$^{+/-}$ mice, following the addition of ADP as a substrate (Figure 6A). There was a significant decrease in respiratory control ratio (RCR) of *Eif6*$^{+/-}$ fibers compared with control, indicating impaired mitochondrial respiration efficiency in complexes III and IV of the electron transport chain (Figure 6B, $p = 0.02$), together with a trend toward an increase in the ADP:oxygen (P:O) ratio (Figure 6C, $p = 0.07$). These observations are consistent with a comparative reduction in oxygen utilization by the electron transport chain in mitochondria from *Eif6*$^{+/-}$ muscle,

OPEN ACCESS
CellPress

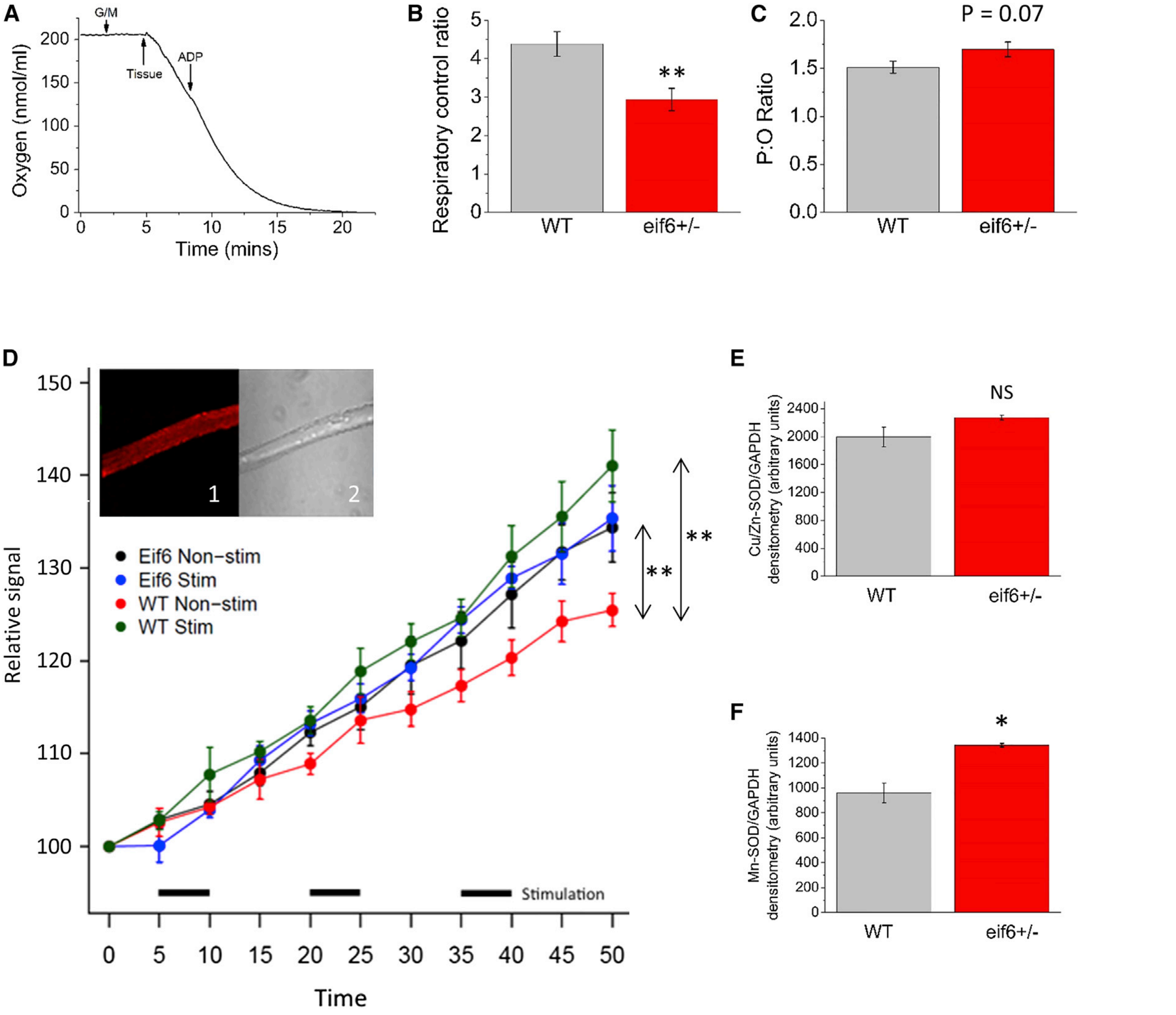

Figure 6. Respiration and ROS Generation in *Eif6* Heterozygote Muscle

(A) Typical oxygen utilization dynamics of muscle tissue before and after addition of ADP to the medium.

(B) Respiratory control ratio (ratio of respiration state 3 to state 4) is significantly lower in *Eif6*$^{+/-}$ (n = 8) muscle compared with WT muscle (n = 8).

(C) P:O ratio is not significantly altered in *Eif6* heterozygous muscle.

(D) Mitosox (405-nm) signal (superoxide abundance) over time in response to electrically stimulated contraction in WT and *Eif6*$^{+/-}$ FDB muscle fibers. Black bars indicate periods of stimulation. Repeated-measures p value: WT versus WT stimulated, p = 0.0016, *Eif6*$^{+/-}$ versus *Eif6*$^{+/-}$ stimulated, p = 0.38, WT versus *Eif6*$^{+/-}$ non-stimulated, $p < 0.01$.

(E and F) SOD1 (E) and SOD2 (F) protein expression measured by western blot in *Eif6*$^{+/-}$ muscle.

Data are represented as mean ± SEM. *$p < 0.05$, **$p < 0.01$.

while a reduced efficiency of respiration suggests an increase in reactive oxygen species (ROS) generation. Electrically stimulated contraction of cultured WT fibers increased superoxide ROS generation as expected ($p < 0.01$), whereas contracting *Eif6*$^{+/-}$ fibers showed no detectable increase in superoxide generation (Figure 6D). However, this was accompanied by a significant increase in baseline generation of superoxide ROS in *Eif6*$^{+/-}$ fibers compared to WT, such that unstimulated *Eif6*$^{+/-}$ fibers were more similar to stimulated WT fibers ($p < 0.01$) (Figure 6D).

Investigating the causes of mitochondrial impairment in haploinsufficient mice, we found that while *Eif6*$^{+/-}$ fibers have no change in copper-zinc superoxide dismutase (CuZn-SOD, SOD1) (Figure 6E), they have significantly higher level of manganese-superoxide dismutase (Mn-SOD, SOD2) compared to WT fibers ($p < 0.04$) (Figures 6F and S5). SOD2 catalyzes the conversion of superoxide generated by "proton leaks" from the electron transport chain into hydrogen peroxide and is essential for cellular health, as it reduces the oxidative damage resulting from ROS production. Hence, the observation of elevated levels of SOD2 in the mitochondria is consistent with the lack of detection of increased levels of superoxide and ROS observed following electrically stimulated contractions in *Eif6*$^{+/-}$ muscle fibers.

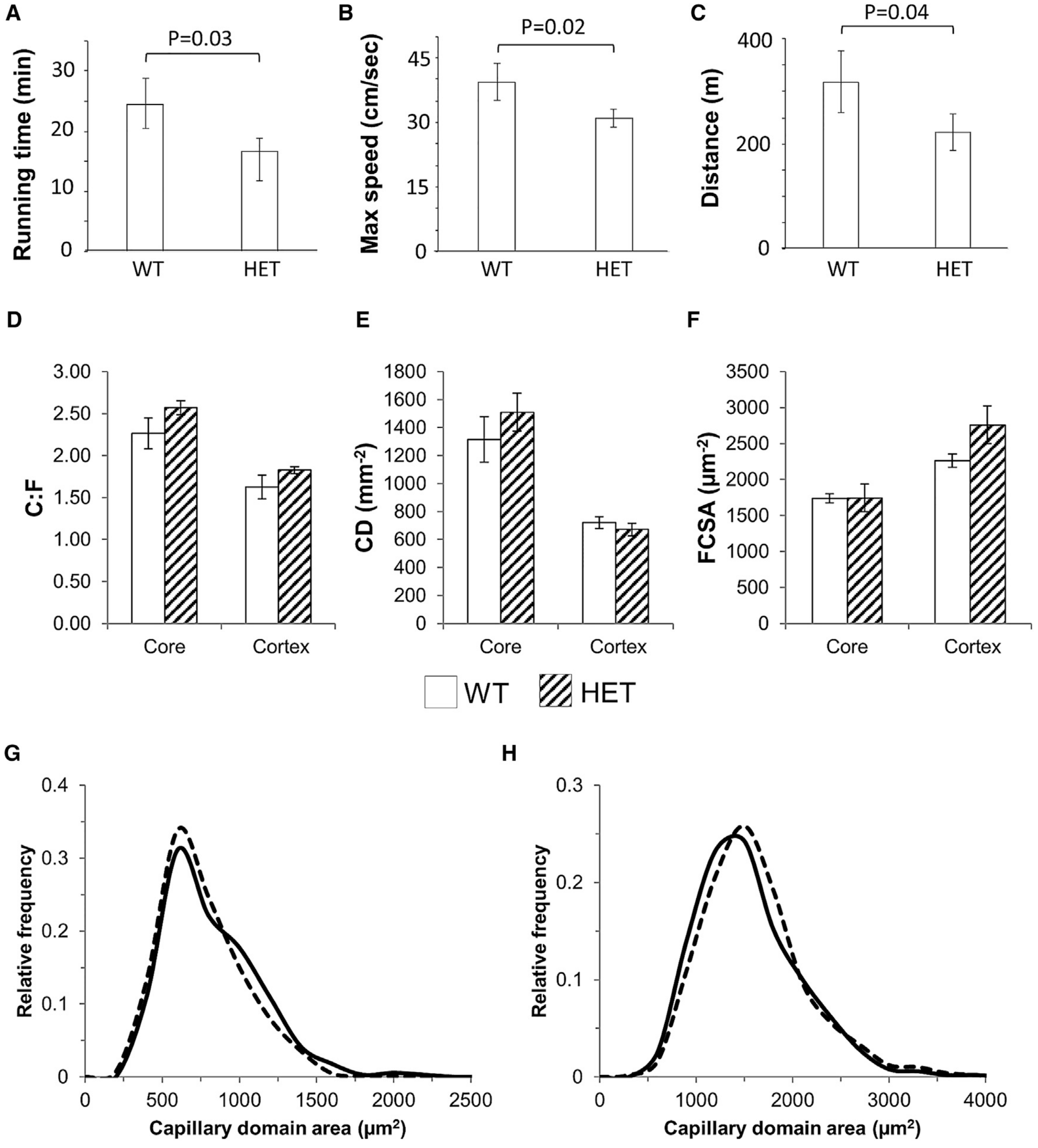

Figure 7. Exercise Capacity of *Eif6* Heterozygote Mice

(A) Running time in minutes to exhaustion of WT and *Eif6*$^{+/-}$ mice.

(B) Maximum speed obtained (cm/s) by WT and *Eif6*$^{+/-}$ mice.

(C) Total distance traveled by WT and *Eif6*$^{+/-}$ mice.

(D–F) Capillary-to-fiber ratio (D), capillary density (E), and fiber-cross sectional area (FCSA) (F) in the oxidative core and glycolytic cortex of WT and *Eif6*$^{+/-}$ skeletal muscle. Data are represented as mean ± SEM.

(G and H) The spatial distribution of capillaries as an index of diffusive limitation for gaseous exchange is shown by the distribution of capillary supply area (domains) in the oxidative core (G) and cortex (H) for WT and *Eif6*$^{+/-}$ skeletal muscle.

Haploinsufficient *Eif6*$^{+/-}$ Mice Perform Less Efficiently than WT Mice in Exercise Tests

The molecular and imaging analysis of muscle fibers isolated from haploinsufficient *Eif6*$^{+/-}$ mice shows that these are less efficient in utilizing oxygen for respiration and are accumulating ROS at a higher rate than WT mice. These observations, together with the prediction of our computational analysis of the HERITAGE gene expression profiling data suggest a causal link between *Eif6* and exercise performance. To test this hypothesis, we performed an exhaustion treadmill test. This demonstrated that indeed *Eif6*$^{+/-}$ mice experience a reduction in exercise performance measured as running time (Figure 7A),

maximum speed (Figure 7B) and distance (Figure 7C). Meanwhile, there was no difference in basal maximal oxygen uptake (VO_2 max), respiratory exchange ratio (RER), or calorie intake monitored by indirect calorimetry in an independent cohort of mice. (Table S6).

Since it is still possible that muscle fiber efficiency may be influenced by changes in capillary density, we measured capillary-to-fiber ratio, capillary density, fiber cross-sectional area and distribution of capillary domain per area in WT and *Eif6*$^{+/-}$ mice post-training. We discovered that none of these parameters are significantly different between WT and heterozygous mice (Figures 7D–7H). This demonstrates that the reduce efficiency of training is not the result of a difference in muscle blood supply but indeed a difference in fiber efficiency supporting the hypothesis of a causal link between *Eif6* and exercise performance.

DISCUSSION

Here, we show that endurance training involves a profound rewiring of gene regulatory networks, a phenomenon that is at least in part dependent on *Eif6*, an mTor-independent translation factor.

Data-Driven Learning of Biological Networks Shows that a Wide Range of Known Homeostasis Pathways Are Rewired following Exercise Training

The network we developed represents a comprehensive view of signaling pathways, regulatory mechanisms, and their connection to effector functions known to be important in muscle plasticity. The signaling component of the model includes pathways that recapitulate known signaling events following the activation of the neuromuscular junction. These include calcium sensors (e.g., CALM3, CAMK2D), calcium-activated potassium channels (e.g., KCNN1, KCNN4), and other voltage-gated ion channels (e.g., KCNA1, KCNH2). The model also captures the connection between the calcium-activated kinase CAMK2D with the transcription factor MEF2A (Zhang et al., 2007), which is known to activate a program of gene expression in skeletal muscle controlling several metabolic and tissue remodeling pathways (Wu et al., 2000), including the structural components of the muscle fiber represented in the MEF2A-connected M7.

The ERBB signaling pathway, including neuregulin (NRG1), is also enriched in the signaling component of the model. The pathway is known to regulate differentiation and metabolic adaptation in response to contractile activity in skeletal muscle (Lebrasseur et al., 2003; Gumà et al., 2010). NRG1 has been shown to induce an oxidative phenotype and improve insulin sensitivity in muscle cells in a similar manner to endurance training (Cantó et al., 2007). Our network reveals a linkage between this pathway and modules representing mRNA processing, muscle fiber constituents and tissue-remodeling factors, supporting the hypothesis that ERBB signaling may control expression of effector function genes in the response to endurance exercise in humans.

The regulatory component of the model includes epigenetic and post-translational modulators (e.g., HDAC3 in M3). Protein deacetylases have been identified as key regulators of skeletal muscle metabolism, controlling mitochondrial function, fatty acid oxidation and ROS production (Gerhart-Hines et al., 2007; Jing et al., 2011; Sun et al., 2011).

We have shown that putative relationships between pathways can be revealed using a data-driven approach to infer and compartmentalize molecular networks in skeletal muscle. While our strategy is effective, it does not allow the inference of true directionality between individual molecules and pathways. Techniques such as dynamic Bayesian networks can overcome this challenge. However, owing to the complexity of large-scale probabilistic models, the sample sizes and computational resources required are currently prohibitive for networks containing large numbers of genes. The number of transcriptomics samples in the HERITAGE study is sufficient to support correlative approaches that produce indirect networks, but not methods that infer directionality.

Eif6, Metabolic Remodeling, and Exercise Performance

The network analysis revealed *Eif6* as the most connected translation factor linked to energy metabolism. Further experimental validation supported the hypothesis that this linkage may be a causal one. Previous studies exploring the relationship between translation and energy metabolism have predominantly focused on the activity of the master regulator and nutrient sensor mTOR. Indeed, one of the most critical factors responsible for metabolic adaptation, PGC1α, has been shown to be downstream of mTOR (Cunningham et al., 2007). Importantly, since *Eif6* can act independently of mTOR (Ceci et al., 2003; Brina et al., 2015) and considering our results, we conclude that this may represent an alternative mTOR-independent pathway for metabolic adaptation.

We have shown that in liver and fat tissue *Eif6* controls lipid synthesis and glycolysis by stimulating translation of an upstream open reading frame (uORF) and G/C-rich-containing mRNAs (Brina et al., 2015). Here, we have confirmed the striking capability of *Eif6* to regulate metabolism in muscle. Similarities and differences with previous studies in liver, fat (Brina et al., 2015), and bone marrow (Ricciardi et al., 2015) have emerged. Briefly, *Eif6* depletion in muscle now confirms that in all insulin-responsive organs, the partial loss of *Eif6* is associated with a signature involved in modulated insulin sensitivity (Brina et al., 2015). In addition, *Eif6* levels seem to affect the general acetylation profile of metabolic proteins. However, two tissue-specific differences emerge: in muscle, and contrary to other tissue, lipid synthesis is not a primary target of *Eif6* regulation in muscle cells.

The alterations in metabolism, including decreased oxygen utilization efficiency and increased ROS production, may be responsible for the observed reduced performance in a treadmill exercise test in the *Eif6*$^{+/-}$ mice. However, treadmill performance is not dictated solely by muscle performance. The effects of *Eif6* reduction in other tissues responsible for respiration and cardiovascular function, such as cardiac and lung, remain to be elucidated. The fact that mutant mice do not display any change in the fiber-type composition and blood supply strongly support the hypothesis that *Eif6* is causally linked to muscle fiber efficiency, both in energy production and in performance training.

Relevance of *Eif6* in Pathogenesis

In conclusion, *Eif6* could have an important role to play in endurance training. *Eif6* is already considered a rate limiting step in

transformation and has been shown to regulate entry to the cell cycle (Gandin et al., 2008). This raises the possibility that *Eif6* may play an important role in other contexts.

Interestingly, our network analysis revealed a correlation between *Eif6* expression and a marker of systemic inflammation, CRP. Alterations in the level of ROS, which we observe in *Eif6*$^{+/-}$ muscle fibers, can upregulate expression of inflammatory genes including CRP (Wei et al., 2008). This raises the possibility that *Eif6* is important in chronic inflammatory diseases. We found evidence from several mouse and human studies that *Eif6* mRNA levels were significantly altered in diverse pathological and physiological conditions. This included diseases such as Alzheimer's disease, which exhibited a significant decrease in *Eif6* expression in neuronal cells (Figure S6A). Alzheimer's disease is known to involve extensive metabolic changes and ROS induced damage (Dumont and Beal, 2011). In mice, *Eif6* expression was significantly upregulated in response to a combination of a high-fat diet and metformin, a drug commonly used to treat diabetes (Setter et al., 2003) (Figure S6B). Skeletal muscle denervation and innervation, which strongly affect mitochondrial function (O'Leary et al., 2012), including ROS production (Muller et al., 2007), also profoundly altered the expression of *Eif6* in mice (Figure S6C), strongly supporting an important role of *Eif6* in muscle plasticity. Together, these support an interesting possibility that *Eif6* may be involved in a wide spectrum of diseases.

EXPERIMENTAL PROCEDURES

HERITAGE Dataset Gene Expression Analysis

The experimental design and exercise training protocol of the HERITAGE Family Study have been described previously (Bouchard et al., 1995). Participants were sedentary at baseline and normotensive. Each participant exercised three times per week for 20 weeks on cycle ergometers controlled by direct heart rate (HR) monitoring. Briefly, participants exercised at the HR associated with 55% of baseline VO_2 max for 30 min per session for the first 2 weeks. The duration and intensity were gradually increased every 2 weeks, until reaching 50 min and 75% of the HR associated with baseline VO_2 max. This level was maintained for the final 6 weeks of training.

Muscle biopsies of *vastus lateralis* were obtained at baseline and post-training using the percutaneous needle biopsy technique. Total RNA was isolated from frozen muscle biopsies preserved in Tissue-Tek using Trizol, and mRNA was amplified with Ambion MessageAmp Premier following the manufacturer's instructions as previously described (Phillips et al., 2013). Affymetrix HG-U133+2 arrays were used to measure global gene expression levels in baseline and in post-training samples. After removing participants for whom either pre- or post-training arrays failed quality control procedures or were not generated, 41 participants remained. The biopsies were performed between 1994 and 1997. The Affymetrix microarrays we performed in 2012 and all samples were profiled at the same time. No sign of bias or batch effects were detected using cluster analysis, principal component analysis, and other data exploration approaches. Genes differentially expressed in response to training were identified using a paired-design two-class significance analysis of microarrays (SAM) (Tusher et al., 2001) with a FDR threshold of 5%.

Differential Co-expression Networks

To identify genes that showed alterations in correlation patterns (differential co-expression) between trained and untrained subjects, we applied a slightly modified pipeline based on the DiffCoEx procedure (Tesson et al., 2010). DiffCoEx is designed to identify correlation pattern changes using the weighted gene co-expression network analysis (WGCNA) (Langfelder and Horvath, 2008) framework. Briefly, two datasets were defined (pre and post-training), followed by gene cluster identification based on the topological overlap or similarity of differential co-expression between the datasets (Langfelder et al., 2008). We then applied a filter to each gene cluster to remove non-significant changes in gene-gene correlation. A resampling procedure was used to generate two datasets of randomly permuted data. This was then used to calculate a null distribution of co-expression changes, which, in turn, was used to calculate a p value for each gene-gene interaction. Differential co-expression changes with an FDR > 1% were then removed. The standard DiffCoEx resampling procedure was then performed using the filtered clusters. This involved constructing 1,000 sets of random clusters of the same distribution of sizes to build a null distribution of module-module dispersion statistics (a measure of the module-module differential co-expression). Module-module connections with an FDR < 10% were selected as edges within the network. All modules were significant for within-module changes in co-expression.

Data Integration

The HERITAGE gene expression data were used to infer a network-integrating eIF gene expression profiles, KEGG pathways, and physiological measurements (Table S1). Genes differentially correlated to eIF genes between trained and untrained individuals were linked to KEGG pathways using a gene set enrichment analysis (GSEA) approach (Subramanian et al., 2005). Over-represented KEGG pathways (FDR < 10%) were visualized in the network as an edge between the eIF gene and the KEGG pathway.

EIF genes were linked to physiological measurements using linear regression. A simple model of the effect of training on the physiological variable as a function of pre-training gene expression levels was used to generate p values for each relationship. Significant relationships (FDR < 10%) were visualized in the same manner as KEGG pathways.

Gene Ontology Analysis

The R library clusterProfiler (Yu et al., 2012) was used to test for functional enrichment of gene ontology and KEGG pathway terms within gene lists. A term was considered significantly enriched at a FDR less than 10%.

Animal Studies

All animal work was conducted according to relevant national and international guidelines and approved by the University of Birmingham, UK, Medical School and University of Liverpool, UK, Medical Services Unit ethics committee and by the responsible authority of the Regierung von Oberbayern, Germany. Young (8- to 16-week old) male *Eif6*$^{+/-}$ or *Eif6*$^{+/+}$ C57BL6 littermates were sacrificed by cervical dislocation, and skeletal muscle was immediately removed, cleaned of any excess fat or connective tissue, and either flash frozen in liquid nitrogen or placed in the relevant medium for immediate use. Tissue placed in storage was held at -80°C until further use.

Statistical Analysis

We used unpaired Student's t tests for two comparison groups unless stated otherwise, where N represents the number of replicates in each group. Where multiple tests were carried out, p values were corrected using the FDR of Benjamini and Hochberg (1995).

Indirect Calorimetry

To evaluate energy metabolism under baseline conditions, a 21-hr indirect calorimetry trial was conducted in single caged mice at the age of 12 weeks having free access to food and water (32-cage PhenoMaster with activity and drinking/feeding monitoring, TSE Systems, Bad Homburg, Germany). Data were collected in 20-min intervals, resulting in a total of 63 time points per 21 hr. The following variables were assessed: oxygen consumption (VO_2), carbon dioxide production (VCO_2), the respiratory exchange ratio (VCO_2/VO_2), food consumption, locomotor activity (distance traveled in cm / 20 min) and rearing behavior (counts/20 min), body weight, and rectal body temperature. All VO_2 data were used to calculate mean oxygen consumption, and lowest and highest single readings were identified as minimum and maximum VO_2 under baseline conditions. Oxygen consumption data were analyzed using linear regression modeling, including body mass as a covariate to normalize for body mass differences.

Treadmill Test

Two groups of mice (WT, n = 9; Eif6 het, n = 8; age- and sex-matched) were subjected to an exhaustion treadmill test. Each mouse was placed on the belt of a 6-lane motorized treadmill supplied with shocker plates. The treadmill was run at an inclination of +5°. The speed was initially 15 cm/s and then increased by 2 cm/s every 2 min using a sight electric stimulation of 0.3 mA. The test was stopped when the mouse remained on the shocker plate for more than 5 s without attempting to re-engage the treadmill, and the time to exhaustion was determined.

Immunochemistry

Cross-sections (10–12 μm) were cut from the mid-belly of the quadriceps, *gastrocnemius*, and *soleus* muscles on a Bright OTF5000 cryostat (Bright Instrument, England) at −22°C, air-dried, and stored at −80°C prior to further processing. Following thawing at room temperature and to distinguish type 1, 2A, and 2B/X muscle fibers, cross-sections were stained for ATPase after pre-incubation at pH 7.0 as previously described (Tunell and Hart, 1977). From a region of the cross-section containing more than 1 fiber type, an average of 270 and 210 fibers were counted for each WT and *Eif6*$^{+/-}$, respectively. Data are expressed as mean ± SD for n = 4 WT and n = 4 *Eif6*$^{+/-}$ animals.

Capillaries were labeled with fluorescently tagged lectin I (GSL I, Vector Labs). Digitized images of stained sections were used to determine the x and y coordinates for muscle fiber boundaries and the associated capillary coordinates; in-house software was used to calculate the capillary-to-fiber ratio (C:F), capillary density (CD, mm^2), and mean fiber cross-sectional area (FCSA, μm^2). The area of tissue supplied by individual capillaries (the domain of influence) was calculated as a tessellation of non-overlapping polygons, representing the area of tissue closer to one capillary than any other. Under conditions of maximal flow, assuming supply capacity to be similar for all capillaries, the domain size will be inversely proportional to the metabolic demand. The distribution of capillary domain area provides an index of the heterogeneity of capillary supply. The log-normal distribution is best expressed as the standard deviation of the log-transformed area (logSD) (Al-Shammari et al., 2014).

Western Blotting

For whole-muscle western blot analysis, approximately 50 mg *gastrocnemius* muscle was ground to a powder using a mortar and pestle in the presence of liquid nitrogen. Thereafter, the powder was resuspended in an amount of RIPA buffer (Sigma), homogenized for 60 s, and then centrifuged at 7,000 × *g* for 5 min. The supernatant was collected and protein concentration determined using the BradfordUltra method.

Samples were diluted in Laemmli buffer to yield between 20–60 μg of protein per sample that were separated by a 12% SDS-PAGE with proteins subsequently being transferred to nitrocellulose and thereafter probed against Cu/Zn SOD (SOD1) (Enzo), MnSOD (SOD2) (Abcam), NDUFS4 (Abcam), ENO3 (Abcam), and anti-eIF6 (produced in-house).

qPCR

Mitochondrial number was estimated using a real-time PCR-based method. Briefly, total DNA was isolated from a muscle sample (2–5 mg) using the DNeasy Kit (QIAGEN, USA). Nuclear DNA (nuclear DNA) was quantified using primers for glyceraldehyde 3-phosphate dehydrogenase (*Gapdh*); forward 5′CCCACTAACATCAAATGGGG3′ and reverse 5′TCTCCATGGTGGTGAAGACA3′ (amplicon 76). mtDNA was quantified using primers for the mitochondrial encoded cytochrome b; forward 5′CCACTTCATCTTACCATTTATTATCGC3′ and reverse 5′TTTTATCTGCATCTGAGTTTGATCCTGT3′ (amplicon 110). Separate reactions for *Gapdh* and cytochrome b were set up using approximately 12.5 ng of total DNA using the SensiMix Sybr Green Master Mix (Bioline, UK). Real-time PCR was performed using a Bio-Rad iCycler with an iCycler iQ multicolor real-time PCR detection system (Bio-Rad, USA) over 26 cycles and a 62°C annealing temperature. The ratio of nuclear DNA:mtDNA was calculated from the cycle threshold (CT) values and compared between WT and *Eif6*$^{+/-}$ mice.

Microarray Analysis of *Eif6*$^{+/-}$ Muscle

Frozen *soleus*, *gastrocnemius*, and *tibialis anterior* muscle was homogenized in RLT buffer (QIAGEN) with β-mercaptoethanol (1% v/v) using a Precellys-24 tissue homogenizer system (Precellys, UK). Each tissue lysate was centrifuged at 13,000 rpm for 10 min, and the supernatant was removed for RNA extraction. RNA was isolated using QIAGEN RNeasy columns following the manufacturer's instructions. Sample purity was assessed by measuring absorbance at 260 and 280 nm using a NanoDrop spectrophotometer, and all absorbance ratios were between 1.8 and 2.0. Cy3-labeled cRNA was generated using the Agilent Low-Input QuickAmp Kit following the manufacturer's instructions. 600 ng of labeled cRNA was hybridized to Agilent Sureprint G3 Mouse whole-genome microarrays, which were then washed and scanned in an Agilent SureScan microarray scanner. Microarray data were $\log_2$ transformed and normalized using quantile normalization. Differentially expressed genes were detected using SAM with a 5% FDR cutoff.

Polysome Purification and Polysomal mRNA analysis

The polysome analysis followed a modified protocol from Kubica et al. (2005). For a full description of the protocol, see the Supplemental Experimental Procedures. Briefly, 180 μg of muscle lysate was layered on a 15%–50% linear sucrose gradient and centrifuged at 37,000 rpm for 170 min. The sucrose gradient was fractionated and UV absorption at 260 nm was recorded. RNA was precipitated by adding 2 volumes of 100% ethanol, redissolved in RNase-free water, and treated with phenol-chloroform. Sucrose fractions containing ≥2 polysomes were pooled for subsequent analysis. Total RNA was extracted from muscle using the methods of Caddick et al. (2006). The mRNA was then processed for microarray analysis as described above. Statistically significant genes were selected using an absolute $\log_2$-fold change threshold greater than 1 and $p < 0.05$.

Mitochondrial Proteomics

For a full description of the protocol, see the Supplemental Experimental Procedures. Briefly, mitochondria were isolated from fresh *gastrocnemius* muscle of WT and *Eif6*$^{+/-}$ mice as previously described (Wittig et al., 2007). Following digestion and liquid chromatography (LC) separation, samples were analyzed on a Quadrupole-Orbitrap instrument in data-dependent positive (ESI+) mode to automatically switch between full-scan mass spectrometry (MS) and MS/MS acquisition. Raw data files were uploaded into Proteome Discoverer (v.1.3) and searched against the mouse UniProt database using the Mascot search engine (v.2.4.1). The threshold for detected proteins was adjusted to a FDR of less than 5% and imported into Progenesis. Differentially expressed proteins were detected using ANOVA, followed by adjustment to a FDR of 10%.

The enrichment of differentially expressed proteins within the polysomal fraction of highly translated mRNAs in *Eif6*$^{+/-}$ muscle was determined using GSEA. Proteins within the "core enriched" fraction of differentially expressed proteins were considered overlapping between the two experiments (Table S4).

Acetylome Analysis

For a full description of the method, see the Supplemental Experimental Procedures. Total protein from *gastrocnemius* muscle was extracted (3 WT and 3 *Eif6*$^{+/-}$ mice) following the method of Lambertucci et al. (2012). Pre-cleaned protein lysates were rotated overnight at 4°C with protein-G-conjugated anti-acetyl lysine antibody. Anti-acetyl lysine immunoprecipitated proteins from WT or *Eif6*$^{+/-}$ mice were labeled using the TMT Sixplex Isobaric Mass Tagging Kit (Thermo Scientific) following the manufacturer's instructions. Samples were analyzed using an Orbitrap Velos ETD mass spectrometer (Thermo Scientific) and Proteome Discoverer (v.1.3) software.

Oxytherm Analysis

Gastrocnemius muscle from WT and *Eif6*$^{+/-}$ mice was dissected and immediately placed in tissue culture media on ice. Oxygen consumption was measured using a Clark-type oxygen electrode (Hansatech, UK). All measurements were completed at 30°C using 20-mg muscle fiber bundles permeabilized using saponin (50 μg/ml) in ROS buffer (pH 7, in millimolars: 1 $MgCl_2$, 10 imidazole, 2 EGTA, 100 KCl, and 10 KH_2PO_4). Muscle bundles were then washed three times to remove saponin. Respiration was initiated in fresh ROS buffer by the addition of 5 μM glutamate/malate (GM) in the absence (state 4) and in the presence (state 3) of 500 μM ADP. Respiratory control ratios

were calculated as state 3 divided by state 4 respiration rates. P:O ratio was calculated as the ratio of the amount of nucleotide added and molecular oxygen consumed during state 3 respiration.

Muscle Fiber Extraction

Single fibers were isolated from the *flexor digitorum brevis* (FDB) muscle of mice as previously described (Pearson et al., 2014). Muscles were incubated for 1.5 hr at 37°C in 0.4% (w/v) sterile type 1 collagenase (EC 3.4.24.3, Sigma Chemical, UK) in minimum essential medium eagle (MEM) containing 2 mM glutamine, 50 IU penicillin, 50 μg/ml streptomycin, and 10% fetal bovine serum (FBS) (Sigma Chemical). The muscles were agitated every 30 min during the digestion period to release single fibers and thereafter were washed three times in MEM containing 10% FBS. Fibers were plated onto pre-cooled 35-mm glass-bottomed cell culture dishes (MatTek, MA, USA) pre-coated with Matrigel (BD Biosciences, Oxford, UK) and were allowed to attach.

ROS Imaging

For a full description of the method, see the Supplemental Experimental Procedures. Briefly, fibers were incubated in 2 mL D-PBS containing 250 nM MitoSox Red or 10 μM DAF-FM DA for 30 min at 37°C. FDB fiber contraction was induced using platinum electrodes using field stimulations at 5–10, 20–25, and 35–40 min over a 50-min protocol. Images were collected at 5-min intervals using a Nikon E-Ti inverted microscope (TI-S-EJOY, Nikon). Data were tested by general linear models with repeated measures (examining stimulation and genotype) or Student's t test as indicated.

DATA AND SOFTWARE AVAILABILITY

The accession number for the gene expression data reported in this study is GEO: GSE47874.

SUPPLEMENTAL INFORMATION

Supplemental Information includes Supplemental Experimental Procedures, six figures, six tables, and two data files and can be found with this article online at https://doi.org/10.1016/j.celrep.2017.10.040.

AUTHOR CONTRIBUTIONS

Conceptualization, K.C. and F.F.; Methodology, K.C.; Formal Analysis, K.C., S.R., T.P., P.K.D., D.M.S., F.K., J.A., C.S., and F.F.; Investigation, K.C., S.R., T.P., I.B., M.B., J.R., D.B., A.S., D.M.S., F.K., J.A., M.A.S., S.G., A.P., C.S., and S.E.; Resources, S.B., D.B., and M.H.d.A.; Writing – Original Draft, K.C., J.A., and F.F.; Writing – Review & Editing, K.C., S.R., T.P., P.K.D., D.M.S., R.J.B., M.A.S., S.J., A.P., S.E., M.C., S.B., and F.F.; Visualization, K.C., S.R., T.P., C.S., and F.F.; Supervision, R.J.B., C. Bunce, C.S., S.E., M.C., M.J., C. Bouchard, S.B., and F.F.

ACKNOWLEDGMENTS

We thank Annarita Miluzio (INGM, Milan) for her invaluable work maintaining the *Eif6* mouse model. This work has been funded by an ERC "Translate" grant (project no. 338999 to S.B.), a BBSRC doctoral training grant (BB/GO18049/1 to K.C.), and the European Commission FP7/2007-2013 Programme SYNERGY-COPD (project no. 270086 to F.F.).

Received: November 17, 2016
Revised: August 16, 2017
Accepted: October 11, 2017
Published: November 7, 2017

REFERENCES

Al-Shammari, A.A., Gaffney, E.A., and Egginton, S. (2014). Modelling capillary oxygen supply capacity in mixed muscles: capillary domains revisited. J. Theor. Biol. *356*, 47–61.

Benjamini, Y., and Hochberg, Y. (1995). Controlling the false discovery rate: a practical and powerful approach to multiple testing. J. R. Stat. Soc. Series B Stat. Methodol *57*, 289–300.

Blair, S.N., and Brodney, S. (1999). Effects of physical inactivity and obesity on morbidity and mortality: current evidence and research issues. Med. Sci. Sports Exerc. *31* (*Suppl 11*), S646–S662.

Bouchard, C., Leon, A.S., Rao, D.C., Skinner, J.S., Wilmore, J.H., and Gagnon, J. (1995). The HERITAGE family study. Aims, design, and measurement protocol. Med. Sci. Sports Exerc. *27*, 721–729.

Brina, D., Miluzio, A., Ricciardi, S., Clarke, K., Davidsen, P.K., Viero, G., Tebaldi, T., Offenhäuser, N., Rozman, J., Rathkolb, B., et al. (2015). eIF6 coordinates insulin sensitivity and lipid metabolism by coupling translation to transcription. Nat. Commun. *6*, 8261.

Caddick, M.X., Jones, M.G., van Tonder, J.M., Le Cordier, H., Narendja, F., Strauss, J., and Morozov, I.Y. (2006). Opposing signals differentially regulate transcript stability in Aspergillus nidulans. Mol. Microbiol. *62*, 509–519.

Cantó, C., Pich, S., Paz, J.C., Sanches, R., Martínez, V., Orpinell, M., Palacín, M., Zorzano, A., and Gumà, A. (2007). Neuregulins increase mitochondrial oxidative capacity and insulin sensitivity in skeletal muscle cells. Diabetes *56*, 2185–2193.

Ceci, M., Gaviraghi, C., Gorrini, C., Sala, L.A., Offenhäuser, N., Marchisio, P.C., and Biffo, S. (2003). Release of eIF6 (p27BBP) from the 60S subunit allows 80S ribosome assembly. Nature *426*, 579–584.

Colberg, S.R., Sigal, R.J., Fernhall, B., Regensteiner, J.G., Blissmer, B.J., Rubin, R.R., Chasan-Taber, L., Albright, A.L., and Braun, B. (2010). Exercise and type 2 diabetes: The American College of Sports Medicine and the American Diabetes Association: joint position statement. Diabetes Care *33*, 2692–2696.

Cunningham, J.T., Rodgers, J.T., Arlow, D.H., Vazquez, F., Mootha, V.K., and Puigserver, P. (2007). mTOR controls mitochondrial oxidative function through a YY1-PGC-1alpha transcriptional complex. Nature *450*, 736–740.

Davidsen, P.K., Herbert, J.M., Antczak, P., Clarke, K., Ferrer, E., Peinado, V.I., Gonzalez, C., Roca, J., Egginton, S., Barberá, J.A., and Falciani, F. (2014). A systems biology approach reveals a link between systemic cytokines and skeletal muscle energy metabolism in a rodent smoking model and human COPD. Genome Med. *6*, 59.

Dumont, M., and Beal, M.F. (2011). Neuroprotective strategies involving ROS in Alzheimer disease. Free Radic. Biol. Med. *51*, 1014–1026.

Gandin, V., Miluzio, A., Barbieri, A.M., Beugnet, A., Kiyokawa, H., Marchisio, P.C., and Biffo, S. (2008). Eukaryotic initiation factor 6 is rate-limiting in translation, growth and transformation. Nature *455*, 684–688.

Gerhart-Hines, Z., Rodgers, J.T., Bare, O., Lerin, C., Kim, S.-H., Mostoslavsky, R., Alt, F.W., Wu, Z., and Puigserver, P. (2007). Metabolic control of muscle mitochondrial function and fatty acid oxidation through SIRT1/PGC-1α. EMBO J. *26*, 1913–1923.

Gumà, A., Martínez-Redondo, V., López-Soldado, I., Cantó, C., and Zorzano, A. (2010). Emerging role of neuregulin as a modulator of muscle metabolism. Am. J. Physiol. Endocrinol. Metab. *298*, E742–E750.

Jing, E., Emanuelli, B., Hirschey, M.D., Boucher, J., Lee, K.Y., Lombard, D., Verdin, E.M., and Kahn, C.R. (2011). Sirtuin-3 (Sirt3) regulates skeletal muscle metabolism and insulin signaling via altered mitochondrial oxidation and reactive oxygen species production. Proc. Natl. Acad. Sci. USA *108*, 14608–14613.

Kokkinos, P. (2012). Physical activity, health benefits, and mortality risk. ISRN Cardiol. *2012*, 718789.

Kubica, N., Bolster, D.R., Farrell, P.A., Kimball, S.R., and Jefferson, L.S. (2005). Resistance exercise increases muscle protein synthesis and translation of eukaryotic initiation factor 2Bepsilon mRNA in a mammalian target of rapamycin-dependent manner. J. Biol. Chem. *280*, 7570–7580.

Lambertucci, A.C., Lambertucci, R.H., Hirabara, S.M., Curi, R., Moriscot, A.S., Alba-Loureiro, T.C., Guimarães-Ferreira, L., Levada-Pires, A.C., Vasconcelos, D.A.A., Sellitti, D.F., and Pithon-Curi, T.C. (2012). Glutamine supplementation stimulates protein-synthetic and inhibits protein-degradative signaling pathways in skeletal muscle of diabetic rats. PLoS ONE *7*, e50390.

Langfelder, P., and Horvath, S. (2008). WGCNA: an R package for weighted correlation network analysis. BMC Bioinformatics *9*, 559.

Langfelder, P., Zhang, B., and Horvath, S. (2008). Defining clusters from a hierarchical cluster tree: the Dynamic Tree Cut package for R. Bioinformatics *24*, 719–720.

Lebrasseur, N.K., Coté, G.M., Miller, T.A., Fielding, R.A., and Sawyer, D.B. (2003). Regulation of neuregulin/ErbB signaling by contractile activity in skeletal muscle. Am. J. Physiol. Cell Physiol. *284*, C1149–C1155.

Liu, L., Cash, T.P., Jones, R.G., Keith, B., Thompson, C.B., and Simon, M.C. (2006). Hypoxia-induced energy stress regulates mRNA translation and cell growth. Mol. Cell *21*, 521–531.

Morita, M., Gravel, S.P., Chénard, V., Sikström, K., Zheng, L., Alain, T., Gandin, V., Avizonis, D., Arguello, M., Zakaria, C., et al. (2013). mTORC1 controls mitochondrial activity and biogenesis through 4E-BP-dependent translational regulation. Cell Metab. *18*, 698–711.

Muller, F.L., Song, W., Jang, Y.C., Liu, Y., Sabia, M., Richardson, A., and Van Remmen, H. (2007). Denervation-induced skeletal muscle atrophy is associated with increased mitochondrial ROS production. Am. J. Physiol. Regul. Integr. Comp. Physiol. *293*, R1159–R1168.

O'Leary, M.F.N., Vainshtein, A., Carter, H.N., Zhang, Y., and Hood, D.A. (2012). Denervation-induced mitochondrial dysfunction and autophagy in skeletal muscle of apoptosis-deficient animals. Am. J. Physiol. Cell Physiol. *303*, C447–C454.

Pearson, T., Kabayo, T., Ng, R., Chamberlain, J., McArdle, A., and Jackson, M.J. (2014). Skeletal muscle contractions induce acute changes in cytosolic superoxide, but slower responses in mitochondrial superoxide and cellular hydrogen peroxide. PLoS ONE *9*, e96378.

Phillips, B.E., Williams, J.P., Gustafsson, T., Bouchard, C., Rankinen, T., Knudsen, S., Smith, K., Timmons, J.A., and Atherton, P.J. (2013). Molecular networks of human muscle adaptation to exercise and age. PLoS Genet. *9*, e1003389.

Ricciardi, S., Miluzio, A., Brina, D., Clarke, K., Bonomo, M., Aiolfi, R., Guidotti, L.G., Falciani, F., and Biffo, S. (2015). Eukaryotic translation initiation factor 6 is a novel regulator of reactive oxygen species-dependent megakaryocyte maturation. J. Thromb. Haemost. *13*, 2108–2118.

Sanvito, F., Piatti, S., Villa, A., Bossi, M., Lucchini, G., Marchisio, P.C., and Biffo, S. (1999). The beta4 integrin interactor p27(BBP/eIF6) is an essential nuclear matrix protein involved in 60S ribosomal subunit assembly. J. Cell Biol. *144*, 823–837.

Schmutz, S., Däpp, C., Wittwer, M., Vogt, M., Hoppeler, H., and Flück, M. (2006). Endurance training modulates the muscular transcriptome response to acute exercise. Pflugers Arch. *451*, 678–687.

Setter, S.M., Iltz, J.L., Thams, J., and Campbell, R.K. (2003). Metformin hydrochloride in the treatment of type 2 diabetes mellitus: a clinical review with a focus on dual therapy. Clin. Ther. *25*, 2991–3026.

Simoneau, J.A., and Bouchard, C. (1989). Human variation in skeletal muscle fiber-type proportion and enzyme activities. Am. J. Physiol. *257*, E567–E572.

Staron, R.S., Hagerman, F.C., Hikida, R.S., Murray, T.F., Hostler, D.P., Crill, M.T., Ragg, K.E., and Toma, K. (2000). Fiber type composition of the vastus lateralis muscle of young men and women. J. Histochem. Cytochem. *48*, 623–629.

Subramanian, A., Tamayo, P., Mootha, V.K., Mukherjee, S., Ebert, B.L., Gillette, M.A., Paulovich, A., Pomeroy, S.L., Golub, T.R., Lander, E.S., and Mesirov, J.P. (2005). Gene set enrichment analysis: a knowledge-based approach for interpreting genome-wide expression profiles. Proc. Natl. Acad. Sci. USA *102*, 15545–15550.

Sun, Z., Singh, N., Mullican, S.E., Everett, L.J., Li, L., Yuan, L., Liu, X., Epstein, J.A., and Lazar, M.A. (2011). Diet-induced lethality due to deletion of the Hdac3 gene in heart and skeletal muscle. J. Biol. Chem. *286*, 33301–33309.

Teran-Garcia, M., Rankinen, T., Koza, R.A., Rao, D.C., and Bouchard, C. (2005). Endurance training-induced changes in insulin sensitivity and gene expression. Am. J. Physiol. Endocrinol. Metab. *288*, E1168–E1178.

Tesson, B.M., Breitling, R., and Jansen, R.C. (2010). DiffCoEx: a simple and sensitive method to find differentially coexpressed gene modules. BMC Bioinformatics *11*, 497.

Timmons, J.A., Knudsen, S., Rankinen, T., Koch, L.G., Sarzynski, M., Jensen, T., Keller, P., Scheele, C., Vollaard, N.B., Nielsen, S., et al. (2010). Using molecular classification to predict gains in maximal aerobic capacity following endurance exercise training in humans. J. Appl. Physiol. *108*, 1487–1496.

Tunell, G.L., and Hart, M.N. (1977). Simultaneous determination of skeletal muscle fiber, types I, IIA, and IIB by histochemistry. Arch. Neurol. *34*, 171–173.

Turan, N., Kalko, S., Stincone, A., Clarke, K., Sabah, A., Howlett, K., Curnow, S.J., Rodriguez, D.A., Cascante, M., O'Neill, L., et al. (2011). A systems biology approach identifies molecular networks defining skeletal muscle abnormalities in chronic obstructive pulmonary disease. PLoS Comput. Biol. *7*, e1002129.

Tusher, V.G., Tibshirani, R., and Chu, G. (2001). Significance analysis of microarrays applied to the ionizing radiation response. Proc. Natl. Acad. Sci. USA *98*, 5116–5121.

Wang, Y.-X., Zhang, C.-L., Yu, R.T., Cho, H.K., Nelson, M.C., Bayuga-Ocampo, C.R., Ham, J., Kang, H., and Evans, R.M. (2004). Regulation of muscle fiber type and running endurance by PPARdelta. PLoS Biol. *2*, e294.

Wei, Y., Chen, K., Whaley-Connell, A.T., Stump, C.S., Ibdah, J.A., and Sowers, J.R. (2008). Skeletal muscle insulin resistance: role of inflammatory cytokines and reactive oxygen species. Am. J. Physiol. Regul. Integr. Comp. Physiol. *294*, R673–R680.

Wittig, I., Carrozzo, R., Santorelli, F.M., and Schägger, H. (2007). Functional assays in high-resolution clear native gels to quantify mitochondrial complexes in human biopsies and cell lines. Electrophoresis *28*, 3811–3820.

Wu, H., Naya, F.J., McKinsey, T.A., Mercer, B., Shelton, J.M., Chin, E.R., Simard, A.R., Michel, R.N., Bassel-Duby, R., Olson, E.N., and Williams, R.S. (2000). MEF2 responds to multiple calcium-regulated signals in the control of skeletal muscle fiber type. EMBO J. *19*, 1963–1973.

Yu, G., Wang, L.-G., Han, Y., and He, Q.-Y. (2012). clusterProfiler: an R package for comparing biological themes among gene clusters. OMICS *16*, 284–287.

Zhang, T., Kohlhaas, M., Backs, J., Mishra, S., Phillips, W., Dybkova, N., Chang, S., Ling, H., Bers, D.M., Maier, L.S., et al. (2007). CaMKIIdelta isoforms differentially affect calcium handling but similarly regulate HDAC/MEF2 transcriptional responses. J. Biol. Chem. *282*, 35078–35087.

Zhao, S., Xu, W., Jiang, W., Yu, W., Lin, Y., and Zhang, T. (2010). Regulation of cellular metabolism by protein lysine acetylation. Science *327*, 1000–1004.

Zhong, L., D'Urso, A., Toiber, D., Sebastian, C., Henry, R.E., Vadysirisack, D.D., Guimaraes, A., Marinelli, B., Wikstrom, J.D., Nir, T., et al. (2010). The histone deacetylase Sirt6 regulates glucose homeostasis via Hif1alpha. Cell *140*, 280–293.

Cell Reports, Volume *21*

Supplemental Information

The Role of *Eif6* in Skeletal Muscle Homeostasis Revealed by Endurance Training Co-expression Networks

Kim Clarke, Sara Ricciardi, Tim Pearson, Izwan Bharudin, Peter K. Davidsen, Michela Bonomo, Daniela Brina, Alessandra Scagliola, Deborah M. Simpson, Robert J. Beynon, Farhat Khanim, John Ankers, Mark A. Sarzynski, Sujoy Ghosh, Addolorata Pisconti, Jan Rozman, Martin Hrabe de Angelis, Chris Bunce, Claire Stewart, Stuart Egginton, Mark Caddick, Malcolm Jackson, Claude Bouchard, Stefano Biffo, and Francesco Falciani

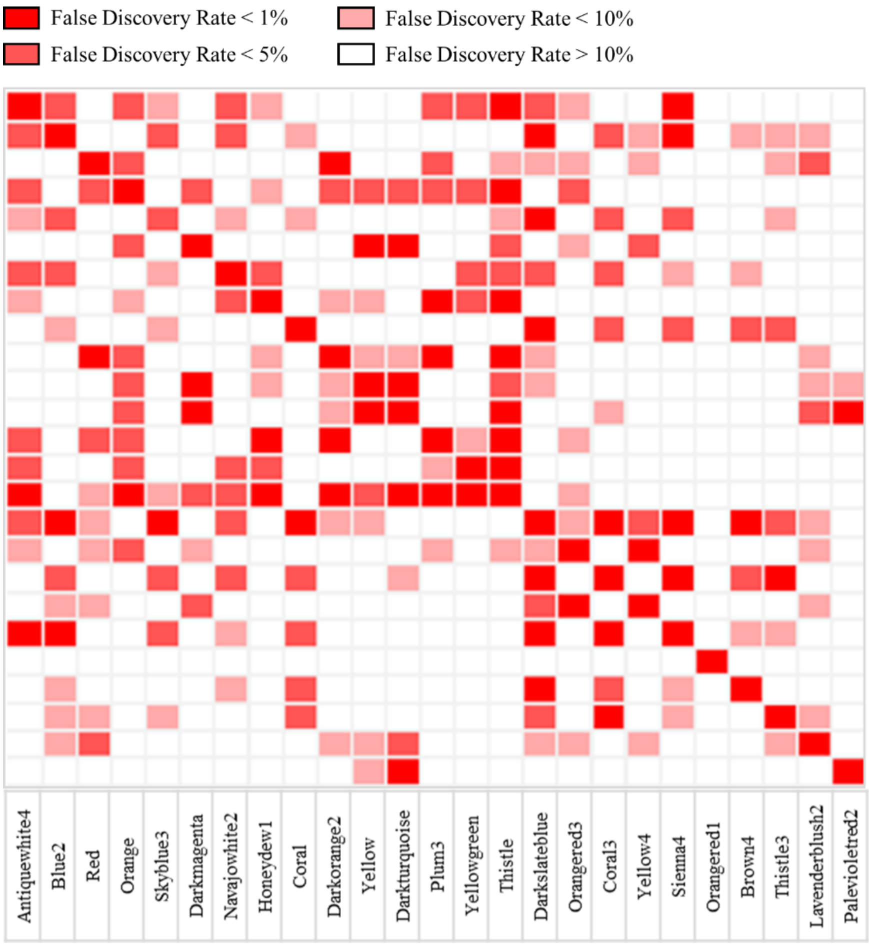

Module Label (DiffCoEx)	Module Size	Module Label (Fig 1)	Enrichment of D.E.G (endurancetraining)
Antiquewhite4	2989	M1	
Blue2	302	M7	
Red	545	M6	
Orange	424	M5	
Skyblue3	1955	M8	
Darkmagenta	660	M3	↓
Navajowhite2	898	M4	
Honeydew1	31		
Coral	447	M9	↓
Darkorange2	175	M10	↓
Yellow	101	M2	
Darkturquoise	68		
Plum3	18		
Yellowgreen	40		
Thistle	18		
Darkslateblue	40		
Orangered3	22		
Coral3	13		
Yellow4	28		
Sienna4	19		
Orangered1	7		
Brown4	42		
Thistle3	21		
Lavenderblush2	16		
Palevioletred2	17		

Fig S1 – Results of the DiffCoEx module-to-module resampling procedure. Related to Figure 1.
The DiffCoEx procedure includes a resampling procedure to ascertain the significance of module-wise changes in co-expression between two conditions, assessed using the dispersion statistic. This heatmap represents the results of that resampling procedure, represented as a false discovery rate for each pairwise module-module test, as well as the module sizes (number of genes), module labels and enrichment of genes differentially expressed in response to training.

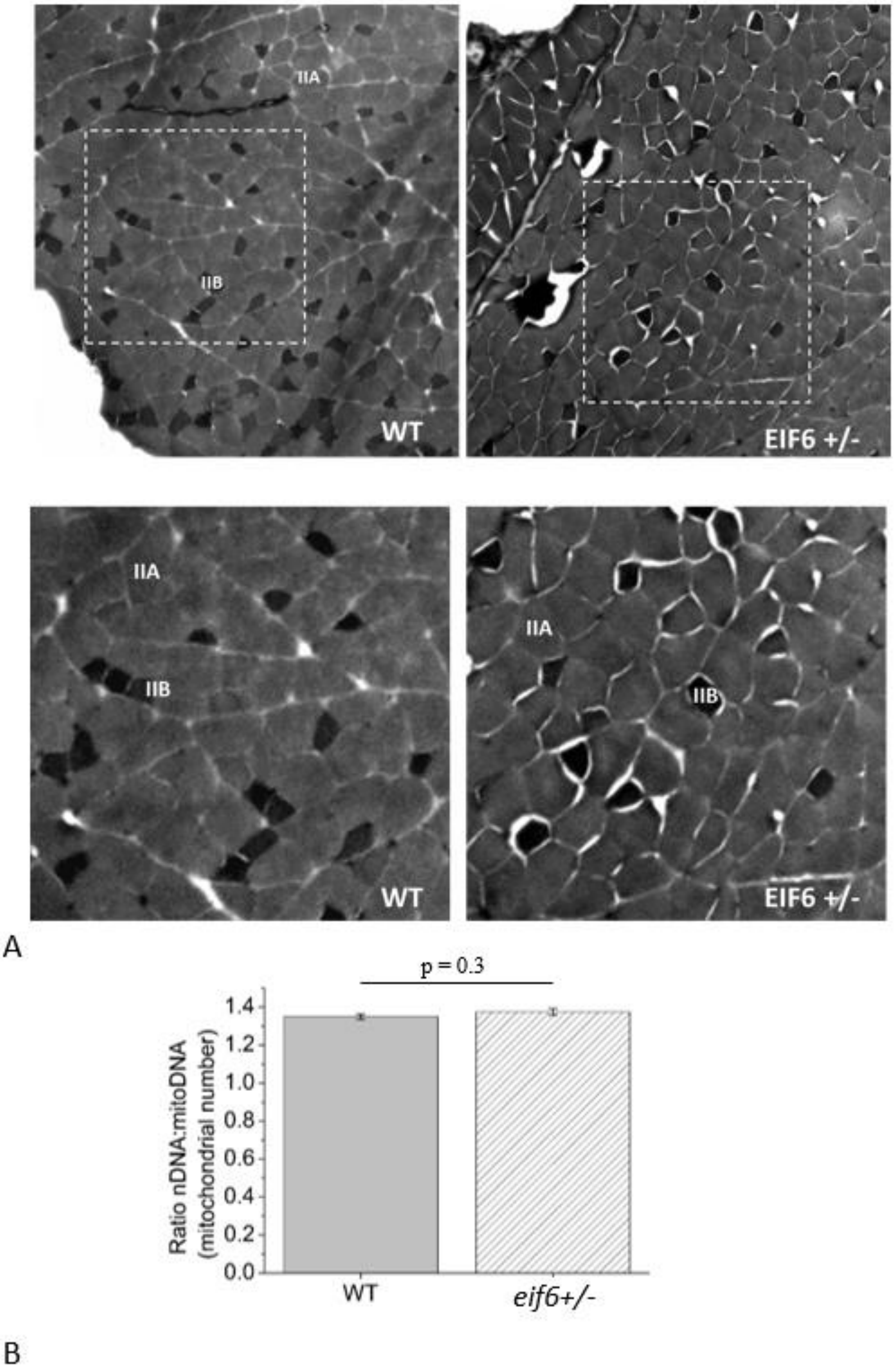

Fig S2 – Fibre type staining and mtDNA/nDNA ratio in *eif6*$^{+/-}$ skeletal muscle. Related to Figure 3.
(A) Representative images of type IIA and IIB fibre type staining in skeletal muscle sections from wild-type and *eif6*$^{+/-}$ mice. (B) Ratio of mitochondrial to nuclear DNA (cytB/Gapdh) in wild-type and haploinsufficient skeletal muscle. Data shown is mean +/- SEM. P-value calculated using the Students T-test. Related to Figure 4.

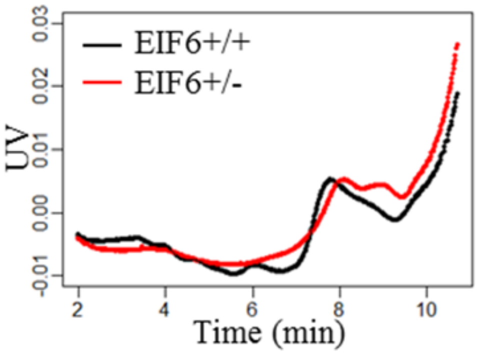

Fig S3 - Representative polysome trace from eIF6 heterozygote and wild type skeletal muscle. Relates to Figure 4.

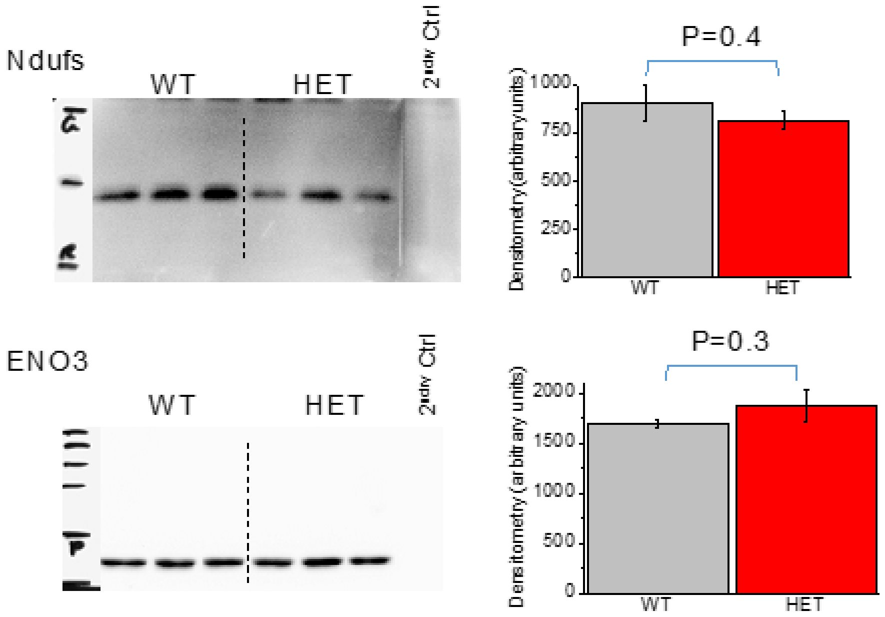

Fig S4 - Western blot analysis of NDUFS4 and ENO3 protein in wild type and *eif6*$^{+/-}$ skeletal muscle. Relates to Figure 5.

Barplots represent mean signal +/- SEM. P-values were calculated using the Students T-test.

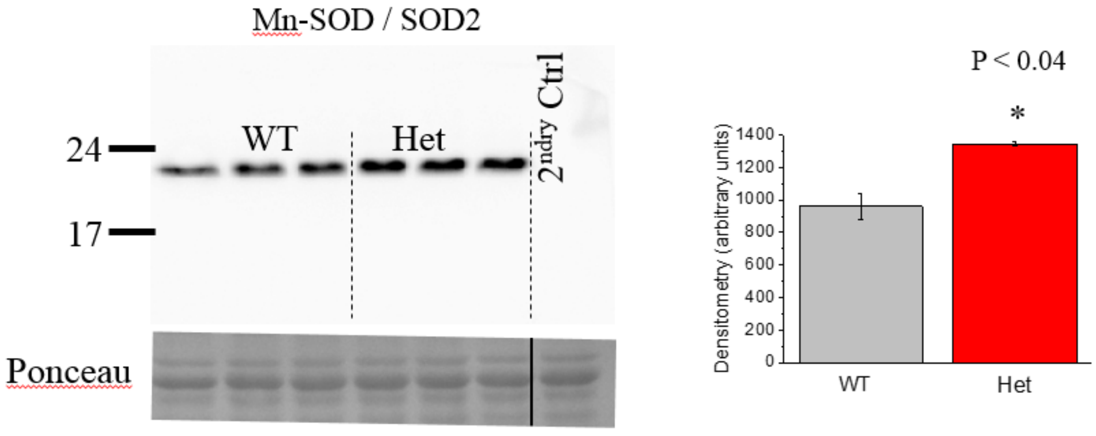

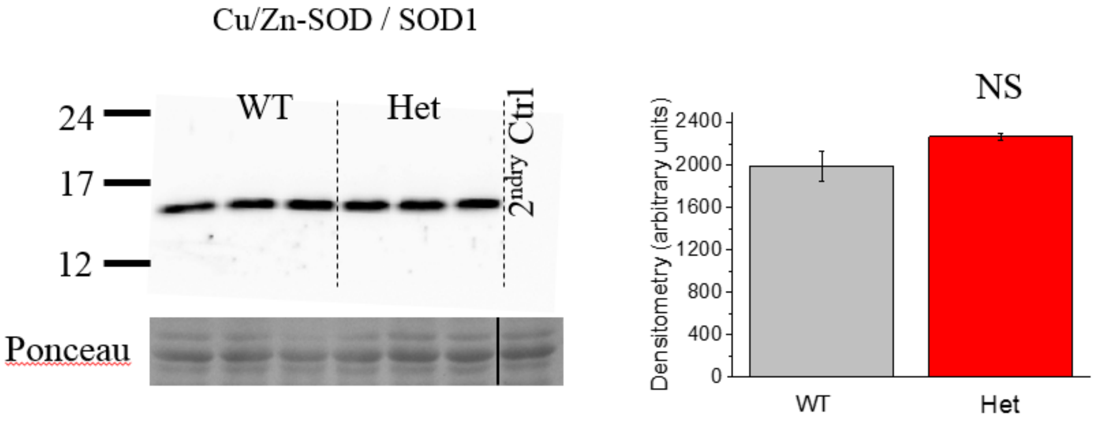

Fig S5 – Western blot analysis of SOD1 and SOD2 protein in wild type and *eif6*$^{+/-}$ skeletal muscle. Relates to Figure 6.

Barplots represent mean signal +/- SEM. P-values were calculated using the Students T-test.

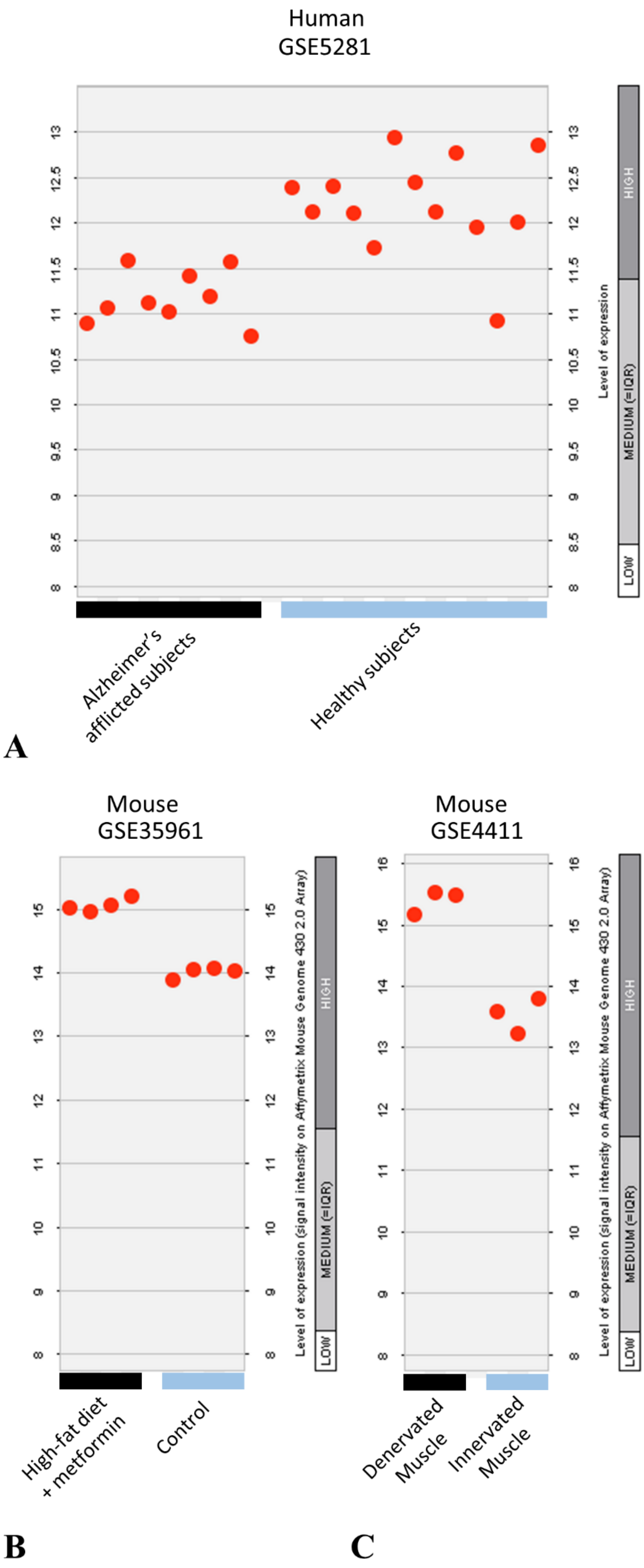
Human
GSE5281
Alzheimer's afflicted subjects
Healthy subjects
Level of expression
HIGH
MEDIUM (=IQR)
LOW
A
Mouse
GSE35961
High-fat diet + metformin
Control
Level of expression (signal intensity on Affymetrix Mouse Genome 430 2.0 Array)
B
Mouse
GSE4411
Denervated Muscle
Innervated Muscle
C

Fig S6 - Differential expression of eIF6 in existing datasets from GeneVestigator, relates to Figure 2.
(A) Cortical layer III pyramidal neurons of the posterior cingulate of clinically and neuropathologically classified late-onset Alzheimer´s disease afflicted individuals or neurologically normal individuals (healthy subjects). Log_2 ratio -1.04. $P < 0.001$
(B) Liver tissue samples obtained from C57BL/6 8 weeks old mice fed with high fat (HF) diet which was methionine- and choline-deficient (MCD) and contained 0.1% metformin (37.5mg/kg) vs regular chow. Log_2 ratio 1.06. $P < 0.001$
(C) Denervated vs innervated tibialis anterior muscles derived from runx f/f control mice. Log_2 Ratio 1.85. $P < 0.001$

Table S1 – Physiological and biochemical measurements accompanying the HERITAGE gene expression cohort, related to Figure 2

Measurement	Description	Units	Mean Pre-training	Standard Deviation Pre-training	Mean Post-training	Standard Deviation Post-training	Trained vs untrained t-test P value
AIRg	Acute insulin response to glucose	pmol/L	680.94	513.59	613.50	386.41	4.87E-01
CK_enz	Creatine kinase	U/g	396.25	69.42	422.25	65.59	1.83E-03
COX_enz	Cyclooxygenase	U/g	7.00	1.63	10.11	2.25	3.72E-11
CPT_enz	Carnitine palmitoyltransferase	U/g	0.12	0.02	0.16	0.02	2.88E-16
CRP	C-reactive protein	mg/L	0.17	0.22	0.23	0.25	4.31E-02
CS_enz	Citrate synthase	U/g	11.51	2.18	17.25	3.55	9.33E-18
fastGlu_BP	Fasting blood glucose	mmol/L	5.10	0.56	5.16	0.58	2.78E-01
fastIns_BP	Fasting blood insulin		62.59	32.73	59.18	25.04	4.06E-01
GAPDH_enz	Glyceraldehyde 3-phosphate dehydrogenase	U/g	429.05	100.21	431.23	85.00	1.35E-01
HADH_enz	3-hydroxyacyl-CoA dehydrogenase	U/g	16.57	2.97	20.95	3.85	4.22E-13
HDL.chol_BP	HDL cholesterol	mmol/L	1.06	0.24	1.15	0.25	3.10E-04
HK_enz	Hexokinase	U/g	2.59	0.50	3.08	0.59	6.39E-07
LDL.chol_BP	LDL cholesterol	mmol/L	2.83	0.81	2.91	0.83	2.90E-01
Leptin_BP	Blood leptin	ng/mL	11.04	8.41	10.09	9.65	2.72E-01
lipoprot_lipase_IM	Lipoprotein lipase (intramuscular)	umol FFA/g/h	0.41	0.17	0.42	0.18	8.60E-01
lipoprot_lipase_released	Lipoprotein lipase	umol FFA/g/h	0.48	0.24	0.45	0.18	3.53E-01
logFFMI	Logged fat free mass index	kg/m2	1.28	0.11	1.28	0.10	9.85E-02
logISI	Logged insulin sensitivity index	mU/L/min	0.56	0.47	0.60	0.25	7.58E-01
PHO_enz	Glycogen phosphorylase	U/g	20.97	4.64	22.60	4.51	5.44E-06
PhoFK_enz	Phosphofructokinase	U/g	58.45	13.25	62.56	14.27	1.05E-03
TG_BP	Blood triglycerides	mmol/L	1.32	0.77	1.25	0.64	1.55E-01
UCP2_IM	Mitochondrial uncoupling protein 2	A.U	36.42	7.78	39.49	6.90	1.42E-03
VO2max	Peak oxygen uptake or maximal aerobic capacity	L O2/min	2.73	0.81	3.11	0.86	5.57E-15

Table S2 – Skeletal muscle fibre type counts in *eif6*$^{+/-}$ and wild type mice, relates to Figure 3

Data representive of over 1,700 fibres per muscle type, n=4 mice per group

**WT SOLEUS muscle**	**%TI**	**%TIIA**	**%TIIB**
**Average**	29.60	68.62	1.78
**STD DEV**	4.70	4.31	0.40
**HET SOLEUS muscle**	**%TI**	**%TIIA**	**%TIIB**
**Average**	33.44	64.54	2.03
**STD DEV**	2.40	3.48	1.48
**P-value**	0.2232	0.2252	0.806
**WT QUAD muscle**	**%TI**	**%TIIA**	**%TIIB**
**Average**	0.00	16.45	83.55
**STD DEV**	0.00	4.18	4.18
**HET QUAD muscle**	**%TI**	**%TIIA**	**%TIIB**
**Average**	0.00	16.51	83.49
**STD DEV**	0.00	1.60	1.60
**P-value**	1	0.98	0.98

Table S3 - Histone deacetylase genes linked to Eif6 modulation in human correlation networks, gene expression studies and polysomal loading studies, related to Figure 3

	Positive correlation with Eif6 expression	Negative correlation with Eif6 expression
Eif6 correlation network (human)	SIRT6, HDAC1, HDAC10	none

	Up-regulated	Down-regulated
Gene expression (Gastrocnemius)	Hdac1, Hdac6, Hdac7, Hdac9, Sirt1, Sirt6	Sirt2

	Increased loading in Eif6+/-	Increased loading in WT
Polysome loading	Sirt4, Sirt5	Sirt6

Table S4 - Differentialy expressed proteins enriched within the highly translated mRNAs in Eif6 heterozygote muscle, related to Figure 4

Gene	Gene Name
Ampd1	adenosine monophosphate deaminase 1(Ampd1)
Apoo	apolipoprotein O(Apoo)
Asph	aspartate-beta-hydroxylase(Asph)
Atp2a1	ATPase, Ca++ transporting, cardiac muscle, fast twitch 1(Atp2a1)
Bsg	basigin(Bsg)
Casq1	calsequestrin 1(Casq1)
Cat	catalase(Cat)
Cbr2	carbonyl reductase 2(Cbr2)
Eef1a2	eukaryotic translation elongation factor 1 alpha 2(Eef1a2)
Epdr1	ependymin related protein 1 (zebrafish)(Epdr1)
Gm5451	predicted gene 5451(Gm5451)
Mrps2	mitochondrial ribosomal protein S2(Mrps2)
Obscn	obscurin, cytoskeletal calmodulin and titin-interacting RhoGEF(Obscn)
Rac1	RAS-related C3 botulinum substrate 1(Rac1)
Rpl18a	ribosomal protein L18A(Rpl18a)
Rpl3l	ribosomal protein L3-like(Rpl3l)
Sypl2	synaptophysin-like 2(Sypl2)
Tmem109	transmembrane protein 109(Tmem109)
Tpp1	tripeptidyl peptidase I(Tpp1)
Acot13	acyl-CoA thioesterase 13(Acot13)
Acp6	acid phosphatase 6, lysophosphatidic(Acp6)
Aldh4a1	aldehyde dehydrogenase 4 family, member A1(Aldh4a1)
Coq7	demethyl-Q 7(Coq7)
Cox15	cytochrome c oxidase assembly protein 15(Cox15)
Cpox	coproporphyrinogen oxidase(Cpox)
Dap3	death associated protein 3(Dap3)
Fundc2	FUN14 domain containing 2(Fundc2)
Gfm2	G elongation factor, mitochondrial 2(Gfm2)
Gls	glutaminase(Gls)
Guf1	GUF1 homolog, GTPase(Guf1)
Hibch	3-hydroxyisobutyryl-Coenzyme A hydrolase(Hibch)
Iars2	isoleucine-tRNA synthetase 2, mitochondrial(Iars2)
Mrps22	mitochondrial ribosomal protein S22(Mrps22)
Mylpf	myosin light chain, phosphorylatable, fast skeletal muscle(Mylpf)
Oat	ornithine aminotransferase(Oat)
Ociad2	OCIA domain containing 2(Ociad2)
Pmpca	peptidase (mitochondrial processing) alpha(Pmpca)
Qrsl1	glutaminyl-tRNA synthase (glutamine-hydrolyzing)-like 1(Qrsl1)
Sdhd	succinate dehydrogenase complex, subunit D, integral membrane protein(Sdhd)
Sucla2	succinate-Coenzyme A ligase, ADP-forming, beta subunit(Sucla2)
Tnnt3	troponin T3, skeletal, fast(Tnnt3)

Table S5 – Significant changes in protein acetylation status in eif6+/- skeletal muscle, related to Figure 5

Gene Symbol	Description	Ratio WT/Het	Replicates
Electron transport chain - Complex 1			
NDUFS1	NADH-ubiquinone oxidoreductase 75 kDa subunit, mitochondrial	2.634	2
NDUFS8	NADH dehydrogenase [ubiquinone] iron-sulfur protein 8, mitochondrial	2.036	2
NDUFS4	NADH dehydrogenase [ubiquinone] iron-sulfur protein 4, mitochondrial	2.007	3
NDUFV1	NADH dehydrogenase [ubiquinone] flavoprotein 1, mitochondrial	1.965	2
Electron transport chain - Complex 3			
UQCRB	Cytochrome b-c1 complex subunit 7	2.243	2
UQCRC1	Cytochrome b-c1 complex subunit 1, mitochondrial	2.382	2
TCA Cycle			
MDH2	Malate dehydrogenase, mitochondrial	0.5	2
Pyruvate Metabolism			
LDHA	L-lactate dehydrogenase A chain	0.579	3
Glycolysis			
ENO3	Beta-enolase 3	0.486	3
GAPDH	Glyceraldehyde-3-phosphate dehydrogenase	0.735	2
TPI1	Triosephosphate isomerase	0.464	3
ALDOA	Fructose-bisphosphate aldolase A	0.512	3
Mitochondrial transport			
VDAC3	Voltage-dependent anion-selective channel protein 3	0.452	3
Amino acid metabolism			
GOT1	Aspartate aminotransferase, cytoplasmic	0.388	3
Muscle energy homeostasis			
CKM	Creatine kinase M-type	0.52	2
Other			
PDLIM5	PDZ and LIM domain protein 5 (Fragment)	0.695	2
MYLPF	Myosin regulatory light chain 2, skeletal muscle isoform	0.569	3
PVALB	Parvalbumin alpha	0.403	2
CYCS	Cytochrome c	0.643	3

Table S6 - Indirect calorimetry and basal respiration measurements of wild-type and Eif6 heterozygote mice, related to Figure 7

	Female				Male						
	Control		Mutant		Control		Mutant		Linear model -Sex	Linear Model - Genotype	Linear Model - Sex:Genotype Interaction
Category	Mean	SD	Mean	SD	Mean	SD	Mean	SD	P Value	P Value	P Value
Body Temp [°C]	36.94	0.68	37.1	0.64	36.769	0.37	36.71	0.43	0.16	**0.764**	0.6
Food Intake [g]	3.9	0.4	3.8	0.5	4.1	0.6	3.9	0.2	0.777	**0.476**	0.538
Mean VO2 [ml/(h animal)]	92.6	8.6	94	6	97.9	5.1	98.7	5.5	0.184	**0.382**	0.631
Minimum VO2 [ml/(h animal)]	66.6	5.2	67.1	6.9	69.2	4.5	71.4	1.6	0.004	**0.125**	0.983
Maximum VO2 [ml/(h animal)]	118.2	8.4	120.1	11.9	125.8	6.7	124.1	8.7	0.114	**0.699**	0.352
Mean RER [VCO2/VO2]	0.99	0.03	0.98	0.04	0.99	0.04	0.99	0.04	0.874	**0.779**	0.716

SUPPLEMENTAL METHODS

Correlation Analysis

To calculate the significance of gene-gene correlations a randomly permuted dataset was used to generate a null-distribution of correlation values. This null-distribution was used to calculate P values for each gene-gene correlation value. P values were adjusted for multiple testing using the Benjamini-Hochberg approach (Benjamini and Hochberg, 1995).

Overlap between DiffCoEx modules and genes differentially expressed in response to training

In order to discern the overlap between genes differentially expressed (**File S4**) and genes differentially correlated between pre and post-exercise individuals we tested each network module for enrichment of genes whose transcription is exercise dependant using Gene Set Enrichment Analysis. Genes within three modules were found to significantly overlap with genes differentially expressed after training. Modules M3, M9 and M10 were enriched with genes down-regulated after training (FDR < 10%).

Polysome Purification and Polysomal mRNA analysis

Muscle tissue (~0.18g) was powdered in a mortar and pestle with liquid nitrogen, and resuspended in 1ml resuspension buffer (10 mM Tris (pH 8.0), 250 mM KCl, 10 mM MgCl2, 0.5% Triton X-100, 2 mM dithiothreitol, 100 µg/ml cycloheximide, 100 units/ml SUPERase In™ RNase inhibitor (Ambion), and Protease Inhibitor Cocktail (Sigma)). The mixture was then homogenized using PowerLyzer® 24 Bench Top Bead-Based Homogenizer (MO-Bio). Homogenates were incubated on ice for 5 min, and then 150 µl of Tween-deoxycholate mix (1.34 ml of Tween 20, 0.66g of deoxycholate, 18ml of sterile water) was added per 1ml of resuspension buffer, and the samples were briefly vortexed. Samples were incubated on ice for 15 min and then centrifuged at 10,000 g for 10 min at 4°C. The resulting supernatant was layered on a 15–50% linear sucrose gradient (20 mM Tris (pH 8.0), 250 mM KCl, and 10 mM MgCl2) and centrifuged in a RPS40T rotor at 37,000 rpm for 170 min at 4°C. Following centrifugation, the sucrose gradient was fractionated and UV absorption at 260 nm was recorded using BioLogic LP (Bio-Rad). RNA was precipitated by adding 2 volumes of 100% ethanol, re-dissolved in RNase-free water, and treated with phenol-chloroform. The RNA was precipitated by addition of 10% 3 M sodium acetate (pH 5.2) and 2.5 volumes of 100% ethanol and re-dissolved in RNase-free water. Sucrose fractions containing ≥2 polysomes were pooled for subsequent analysis. Total RNA was extracted from muscle using the methods of Caddick et al. (Caddick et al., 2006). The mRNA was then processed for microarray analysis as described above. Statistically significant genes were selected using an absolute $\log_2$ fold change threshold greater than 1 and $p<0.05$.

Mitochondrial Proteomics

Mitochondria were isolated from fresh gastrocnemius muscle of WT and *eif6*$^{+/-}$ mice as previously described (Wittig et al., 2007).

*Sample digestion***:** 50µl of 25mM ammonium bicarbonate (ambic) was added to each mitochondrial pellet. 2µl of a 1%(w/v) Rapigest solution in 25mM ambic was added giving a final concentration of ~0.04%(w/v). For digestion, a volume of sample equivalent to 50µg of protein was made up to 80µl with 25mM ambic and 5µl of 1%(w/v) Rapigest added and the samples heated at 80°C for 10min. Samples were reduced by the addition of 5µl of DTT (9.2mg/mL in 25mM ambic) and heated at 60°C for 10min. Samples were cooled and 5µl of iodoacetamide (33mg/ml in 25mM ambic) was added and samples incubated at room temp for 30min in the dark. Trypsin (Sigma: Porcine trypsin sequencing grade) (1µg) was added and the sample was incubated at 37°C overnight.

The digests were acidified by the addition of 1µl of TFA and incubated at 37°C for 45min. Samples were then centrifuged at 17,000 x g for 30min and supernatants transferred to 0.5mL low-bind tubes. Samples were centrifuged for a further 30min and 10µl transferred to total recovery vials for LC-MS analysis. Pre and post-acidification digest (10µl) were analysed by SDS-PAGE to check for the absence of protein and hence complete digestion.

LC Separation- All peptide separations were carried out using an Ultimate 3000 nano system (Dionex/Thermo Fisher Scientific). For each analysis the sample was loaded onto a trap column (Acclaim PepMap 100, 2cm x 75mm inner diameter, C_{18}, 3mm, 100Å) at 5µl/min with an aqueous solution containing 0.1%(v/v) TFA and 2%(v/v) acetonitrile. After 3min, the trap column was set on-line with an analytical column (Easy-Spray PepMap® RSLC 50cm x 75mm inner diameter, C_{18}, 2mm, 100Å) (Dionex). Peptide elution was performed by applying a mixture of solvents A and B. Solvent A was HPLC grade water with 0.1%(v/v) formic acid, and solvent B was HPLC grade acetonitrile 80%(v/v) with 0.1%(v/v) formic. Separations were performed by applying a linear

gradient of 3.8% to 50% solvent B over 95 min at 300nL/min followed by a washing step (5min at 99% solvent B) and an equilibration step (15 min at 3.8% solvent B). 2µl of each sample was injected.

Analyses on a Quadrupole-Orbitrap instrument- The Q Exactive instrument was operated in data dependent positive (ESI+) mode to automatically switch between full scan MS and MS/MS acquisition. Survey full scan MS spectra (*m/z* 300-2000) were acquired in the Orbitrap with 70,000 resolution (*m/z* 200) after accumulation of ions to $1x10^6$ target value based on predictive automatic gain control (AGC) values from the previous full scan. Dynamic exclusion was set to 20s. The 10 most intense multiply charged ions ($z \geq 2$) were sequentially isolated and fragmented in the octopole collision cell by higher energy collisional dissociation (HCD) with a fixed injection time of 100ms and 35,000 resolution. Typical mass spectrometric conditions were as follows: spray voltage, 1.9kV, no sheath or auxiliary gas flow; heated capillary temperature, 275°C; normalised HCD collision energy 30%. The MS/MS ion selection threshold was set to 1×10^4 counts. A 2Da isolation width was set.

Database search and Protein identification- Raw data files were uploaded into Proteome Discoverer 1.3 and searched against the mouse UniProt database using the Mascot search engine (version 2.4.1). A precursor ion tolerance of 10ppm and a fragment ion tolerance of 0.01Da were used with carbamidomethyl cysteine set as a fixed modification and oxidation of methionine as a variable modification. The false discovery rate (FDR) against a decoy database was 1-5%. The data set was analysed using Progenesis 4.1 LCMS label-free quantification software. The raw profile data was uploaded into the software selecting ESI-FTICR. The top 5 MS/MS spectra were exported from the Progenesis software as a Mascot generic file (mgf) and used for peptide identification using the above settings.

Acetylome Analysis

Total protein from gastrocnemius muscle was extracted from muscle tissue from 3 WT and 3 *eif6*$^{+/-}$ mice according to (Lambertucci et al., 2012). Briefly, muscle tissue was homogenized in extraction buffer (100 mM Tris, pH 7.5; 10 mM EDTA; 100 mM NaF; 10 mM sodium pyrophosphate; 10 mM sodium orthovanadate; 2 mM phenylmethanesulfonyl fluoride; and 0.01 mg/mL aprotinin) at 4°C for 30 sec. After homogenization, Triton X-100 was added to a final concentration of 1%, the samples were incubated for 30 min at 4°C and were centrifuged at 13,000× *g* for 20 min at 4°C. The total protein content was determined using bovine serum albumin as the standard.

Anti-acetyl lysine immunoprecipitations- Anti-acetyl lysine antibody (Abcam) or rabbit IgG control antibody (150mg) were dialysed overnight into coupling buffer (100mM $NaHCO_3$, 500mM NaCl, pH8.5) before conjugating to cyanogen bromide-activated Protein G sepharose (Sigma-Aldrich) according to manufacturer's instructions. 5mg of protein lysate from each sample was made up to 400µl in lysis buffer and the samples pre-cleared using 50µl washed Protein G-Sepharose (Sigma-Aldrich). After rotation for 1 hour at 4°C, samples were centrifuged for 5 minutes at 4000rpm and the supernatant transferred to sterile eppendorfs. Pre-cleared protein lysates were rotated overnight at 4°C with Protein G conjugated anti-acetyl lysine antibody. Samples were centrifuged for 10 minutes at 4000rpm 4°C and the unbound fraction (supernatant) frozen at -20˚C. The pellet was washed in 200µl PBS three times, resuspended in 30µl elution buffer (8M urea, 20mM Tris pH 7.5, 100mM NaCl) and incubated for 30 minutes at room temperature with rotation. The samples were then gently centrifuged and the supernatant collected. The elution step was repeated three times and on the third and final incubation the samples were incubated at 55˚C with gentle agitation for 30 minutes before pooling all elution fractions and storing at -20˚C.

Tandem Mass Tagging- Anti-acetyl lysine immunoprecipitated proteins from wt or eIF6 knock down mice were trypsin digested and then labelled using TMT sixplex Isobaric Mass Tagging Kit (Thermo Scientific) according to manufacturer's instructions. Briefly, proteins were precipitated by the addition of 90µl 100mM triethyl ammonium bicarbonate (TEAB) dissolution buffer, the volume adjusted to 200µl with ultrapure water, 10µl 200mM tris(2-carboxyethyl)phosphine (TCEP) reducing reagent added and the mixture incubated at 55˚C for 1 hour. 10µl 375mM iodoacetamide (in TEAB) was added to each sample and incubated for 30 minutes in the dark. 1320µl of acetone pre-chilled to -20˚C was added to each sample and proteins precipitated overnight at -20˚C. Samples were centrifuged at 8000g and the pellets of precipitated protein air dried before resuspending in 100µl 100mM TEAB and 2.5µg trypsin protease. Proteins were digested overnight at 37°C. 41µl of treatment-specific TMT label reagents were incubated with samples for 2 hours at room temperature. The following labels were used for each treatment arm: WT#3-#4-#5 were labelled with 126, 127, 128 and *eif6*$^{+/-}$ #4-#5-#6 were labelled with 129, 130 and 131. The labeling reaction was quenched by incubating the samples with 8µl 5% hydroxylamine for 15 minutes.

Mass spectrometry- All 6 samples were pooled, dried down to 500µl using a Savant SPD111V SpeedVac Concentrator (Thermo Scientific) at 45°C and made up to 1ml with mobile phase A solution (10mM KH_2PO_4, pH3 with phosphoric acid then add 20% CH_3CN). Samples were fractionated by strong cation exchange high

performance liquid chromatography (SXC-HPLC) using an Ettan LC HPLC machine (Amersham Pharmacia Biotech) and a 100x2.1mm polysulfoethyl aspartamide SCX column with UV detection at 214nm. Elution at a flow of 1 ml/min was performed with a linear gradient between mobile phase A and mobile phase B (10mM KH_2PO_4, 500mM KCl, pH3 with phosphoric acid then add 20% CH_3CN) over a 90 minute run. 16 fractions were collected, dried completely using a SpeedVac (as above) before resuspending in 0.1% trifluoroacetic acid and desalted using C_{18} ZipTips according to manufacturer's instructions (Millipore). Desalted samples were dried completely, resuspended in 10µl 0.1% formic acid and analysed using an Orbitrap Velos ETD mass spectrometer (Thermo Scientific) and Thermo Proteome Discoverer 1.3 software. Each fraction was run in triplicate and peptides identified using Swissprot. The raw data files were processed and quantified using Proteome Discoverer software v1. (Thermo Scientific) and searched against the UniProt/SwissProt Human database release version 57.3 (20326 entries) using the SEQUEST HT algorithm. Peptide precursor mass tolerance was set at 10 ppm, and MS/MS tolerance was set at 0.6 Da. Search criteria included the TMT N-Term and lysine as static modifications, carbamidomethylation of cysteine, oxidation of methionine and deamidation of Asn and Gln and lysine as fixed modifications. Searches were performed with full tryptic digestion and a maximum of 3 missed cleavage sites was allowed. The reverse database search option was enabled and all peptide data was filtered to satisfy false discovery rate (FDR) of 5%. The Proteome Discoverer software generates a reverse "decoy" database from the same protein database and any peptides passing the initial filtering parameters that were derived from this decoy database are defined as false positive identifications. The minimum cross-correlation factor (Xcorr) filter was readjusted for each individual charge state separately to optimally meet the predetermined target FDR of 5% based on the number of random false positive matches from the reverse decoy database. Thus each data set has its own passing parameters. Quantitation was performed using a peak integration window tolerance of 0.05 Da with the integration method set as the most confident centroid. Protein ratios represent the median of the raw measured peptide ratios for each protein. Each protein included in our study was identified from at least 2 peptides with high/medium confidence. Proteins recorded as uncharacterized by the software were returned with a gene ID.

ROS imaging

A Nikon E-Ti inverted microscope with a motorised stage (TI-S-EJOY, Nikon) for a 35mm petri dish was used. The C1 confocal (Nikon Instruments Europe BV, Surrey, UK) comprised a diode (UV) 405nm and argon laser with 488nm excitation. Acquisition software was EZC1 V.3.9 (12 bit). MitoSox Red was excited sequentially at 405nm using a diode laser and 488nm using an argon laser, each passing through a main dichroic and secondary beam splitter with the emission collected through a 605/15 filter to a detector. DAF-FM was excited at 488nm and emission recorded between 515/30nm, bright field images were acquired using the 488nm laser to a CCD. The objective was a PlanApo VC x60A/1.2NA/0.27 mm working distance water immersion. Pinhole size was 150 µm with a 1.68 µsec pixel dwell time in all cases. Regions of interest for determination of fluorescence/area were selected and quantified as previously described (Pearson et al., 2014). All experiments were performed at approximately 28°C. Images were captured every 5 minutes of the 30 minute protocol.

FDB fibre contraction was induced using platinum electrodes using field stimulations with a stimulation at 5-10 and 20-25 minutes over a 30 minute protocol. The stimulation comprised a bipolar pulse of 2ms duration for 0.5 sec repeated every 5 secs at 50Hz. Fibres were loaded by incubation in 2ml D-PBS containing 250 nM MitoSox Red or 10µM DAF-FM DA for 30 minutes at 37 °C in a tissue culture incubator. Cells were then washed and maintained in 2ml MEM-solution during the experimental protocol. DAF-FM DA readily diffuses into cells and within the cytoplasm releases DAF-FM by the action of intracellular esterases. DAF-FM is essentially non fluorescent until it is nitrosylated by products of oxidation of NO, resulting in DAF-FM triazole that exhibits about a 160-fold greater fluorescence efficiency (Kojima et al., 1999).

References

Benjamini, Y., Hochberg, Y., 1995. Controlling the false discovery rate: a practical and powerful approach to multiple testing. J. R. Stat. Soc. Ser. B Methodol. 57, 289–300. doi:10.2307/2346101

Caddick, M.X., Jones, M.G., Van Tonder, J.M., Le Cordier, H., Narendja, F., Strauss, J., Morozov, I.Y., 2006. Opposing signals differentially regulate transcript stability in Aspergillus nidulans. Mol. Microbiol. 62, 509–519. doi:10.1111/j.1365-2958.2006.05383.x

Kojima, H., Urano, Y., Kikuchi, K., Higuchi, T., Hirata, Y., Nagano, T., 1999. Fluorescent indicators for imaging nitric oxide production. Angew. Chemie - Int. Ed. 38, 3209–3212. doi:10.1002/(SICI)1521-3773(19991102)38:21<3209::AID-ANIE3209>3.0.CO;2-6

Lambertucci, A.C., Lambertucci, R.H., Hirabara, S.M., Curi, R., Moriscot, A.S., Alba-Loureiro, T.C., Guimarães-Ferreira, L., Levada-Pires, A.C., Vasconcelos, D.A.A., Sellitti, D.F., Pithon-Curi, T.C., 2012. Glutamine Supplementation Stimulates Protein-Synthetic and Inhibits Protein-Degradative Signaling Pathways in Skeletal Muscle of Diabetic Rats. PLoS One 7. doi:10.1371/journal.pone.0050390

Pearson, T., Kabayo, T., Ng, R., Chamberlain, J., McArdle, A., Jackson, M.J., 2014. Skeletal muscle contractions induce acute changes in cytosolic superoxide, but slower responses in mitochondrial superoxide and cellular hydrogen peroxide. PLoS One 9. doi:10.1371/journal.pone.0096378

Wittig, I., Carrozzo, R., Santorelli, F.M., Schägger, H., 2007. Functional assays in high-resolution clear native gels to quantify mitochondrial complexes in human biopsies and cell lines. Electrophoresis 28, 3811–20. doi:10.1002/elps.200700367
